# Substrate-Guided Development of HDAC11-Selective Inhibitors Featuring α‑Amino Amide Zinc-Binding Groups

**DOI:** 10.1021/acsomega.5c08195

**Published:** 2025-10-14

**Authors:** Sebastian Hilscher, Marat Meleshin, Fady Baselious, Cyril Barinka, Wolfgang Sippl, Mike Schutkowski, Cordelia Schiene-Fischer

**Affiliations:** † Department of Enzymology, Charles Tanford Protein Center, Institute of Biochemistry and Biotechnology, 9176 Martin-Luther-University Halle-Wittenberg, 06120 Halle (Saale), Germany; ‡ Department of Medicinal Chemistry, Institute of Pharmacy, 9176 Martin-Luther-University Halle-Wittenberg, 06120 Halle (Saale), Germany; § Institute of Biotechnology of the Czech Academy of Sciences, BIOCEV, Prumyslova 595, 252 50 Vestec, Czech Republic

## Abstract

Histone deacetylases (HDACs) play a pivotal role in various biological pathways and represent interesting drug targets. Therefore, HDAC inhibitors (HDACi) with high isoform selectivity and a zinc-binding group different from hydroxamic acid, because of its low metabolic stability, are required. HDAC11, as a highly potent defatty-acylase, differs from other HDACs in its substrate preference. Starting from this finding, we developed specific inhibitors for HDAC11 based on a peptide containing a fatty-acylated lysine side chain as the selectivity tail. The introduction of different heteroatoms at the fatty acyl residue was used to generate potent zinc-binding groups in combination with the scissile amide bond, as well as to suppress substrate properties of the resulting compounds. Further optimization resulted in a highly potent and selective HDAC11 inhibitor **31**, which exhibits low nanomolar inhibition against HDAC11 without targeting other HDACs and is active in cells. The data presented here may help expand the range of zinc-binding groups utilized in HDAC inhibitors. Furthermore, the concept of the selectivity tail was demonstrated to facilitate straightforward access to selective defatty-acylase inhibitors.

## Graphical abstract

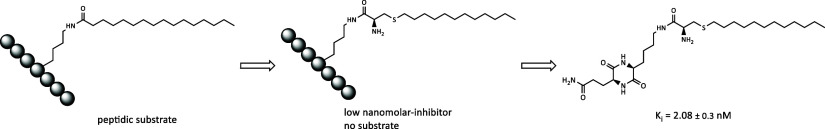

## Introduction

Acetylation of the lysine side-chain nitrogen is one of the most common post-translational modifications of proteins.^1^ The removal of acetyl residues by deacetylation is catalyzed by histone deacetylases (HDACs). Control of the acetylation status of histones by HDACs influences chromatin structure and gene expression, and by this, a multitude of different cellular processes.^2–4^ Deacetylation of nonhistone substrates by HDACs further contributes to the complexity of the acetylation/deacetylation-based regulatory network.

The human HDAC family consists of 18 members, which form 4 different classes based on structural similarities and the enzymatic mechanism.^5^ HDACs of classes I (HDAC1, 2, 3, and 8), IIa (HDAC4, 5, 7, 9, and 10), IIb (HDAC6 and 10), and IV (HDAC11) are Zn^2+^-dependent enzymes, whereas class III HDACs, also referred to as sirtuins (SIRT1–7), require NAD^+^ as the cosubstrate.^6,7^ Several HDACs, namely, SIRT1–3, SIRT5, SIRT6, HDAC8, and HDAC11, have also been found to remove long-chain acyl residues from lysine side chains.^8,9^ Especially, the most recently identified member of the HDAC family HDAC11 was found to be the most proficient defatty-acylase but exhibited minimal deacetylase activity.^8,10–12^ Physiologically, HDAC11 has been shown to act as a deacetylase of histone H3 K9, K14, and K27 and of nonhistone proteins like transcription factors and the cell cycle regulator Cdc25A. The *in vivo* removal of fatty acyl lysine modifications by HDAC11 has been described for serine hydroxymethyl transferase 2 (SHMT2),^4^ a protein involved in type I interferon signaling and for gravin-α, a scaffolding protein influencing β-adrenergic receptor signaling.^2^ HDAC11 plays a role in the maintenance of muscle fiber type balance, the mitochondrial lipid oxidation, and the regulation of the expression of interleukin-10 and p53,^13–16^ and it is involved in the regulation of metabolism and energy homeostasis.^17,18^ Because of its connection to obesity and other metabolic disorders like diabetes and metabolic dysfunction-associated steatotic liver disease (MASLD),^19^ as well as its dysregulation in cancer,^20–22^ HDAC11 is considered as a potential therapeutic target.^23,24^

The development of inhibitors of the deacetylase activity of HDACs of the classes I, II, and IV (HDACi) is mostly based on the use of a zinc-binding group (ZBG) that chelates the catalytic Zn^2+^ ion, supplemented by a cap group, which interacts with the rim at the entrance of the active site of the enzyme and is connected by a linker, mimicking the lysine side chain (Figure 1A). By this approach, often inhibitors that target several or all Zn^2+^-dependent HDACs are obtained. Due to their lack of isozyme specificity, they are referred to as *pan*-HDACi. To date, five *pan*-HDACi are US-FDA-approved drugs for cancer therapy. Four of them, namely, vorinostat (SAHA),^25^ belinostat (PXD-101),^26^ panobinostat (LBH-589),^27^ and givinostat (ITF2357),^28^ contain a hydroxamic acid moiety, the most common ZBG in HDACi (Figure 1B). Unfortunately, small molecules containing hydroxamic acid moieties have been demonstrated to have mutagenic and genotoxic potential, probably caused by highly reactive isocyanates formed by Lossen rearrangement.^29–31^ Furthermore, recent data revealed that hydroxamic-acid-based HDACi *in vivo* caused pronounced off-target effects.^32^ Thus, a variety of other ZBGs, such as benzamides, carboxylic acids, or hydrazides, are under evaluation for their suitability as warheads in HDAC inhibition.^33–36^

**Figure 1:**
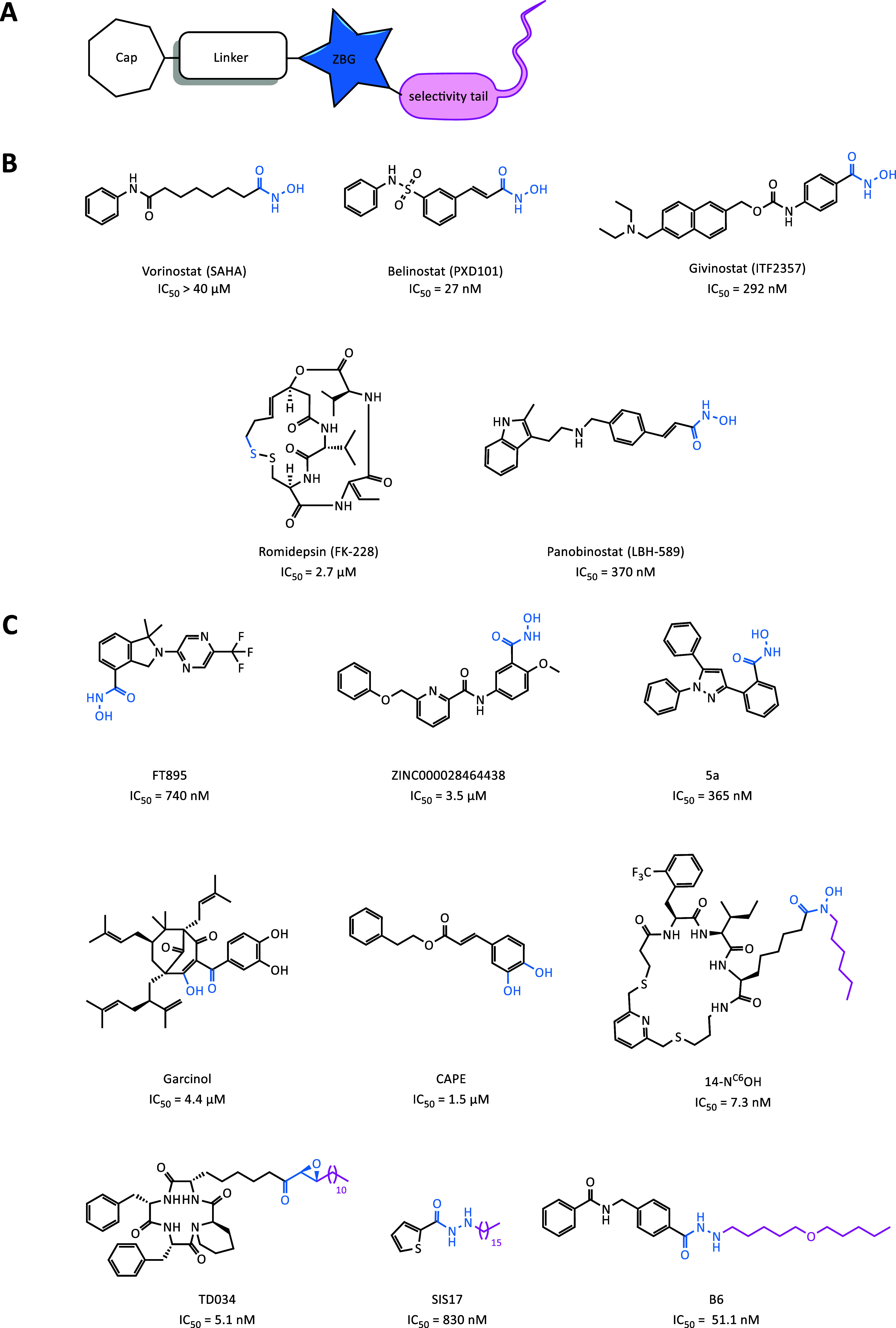
(A) Schematic representation of the structural components of HDAC11-selective inhibitors. The general cap-linker-ZBG pharmacophore is supplemented by a fatty-acyl tail to enhance the HDAC11 binding and selectivity. (B) Structures of current US-FDA-approved *pan*-HDACi.^25–28,44^ The ZBGs are highlighted in blue. IC_50_ values are as reported for HDAC11.^39,45–48^ (C) Structures of selective HDAC11 inhibitors with various ZBGs (blue), with the selectivity tails in pink. The IC_50_ values are as reported for HDAC11.^19,38,39,41–43,49–51^

In contrast to *pan*-HDACi, inhibitors selectively targeted to one distinct HDAC isoform would allow us not only to evaluate the individual role of one specific HDAC in biological processes but also to develop drugs against diseases associated with the activity of a distinct HDAC. Furthermore, toxic side effects associated with *pan*-HDACi may be omitted.^37^

Due to the unique connection of HDAC11 with several pathophysiological conditions, HDAC11-selective inhibitors are highly desirable and envisioned to have unique therapeutic potential, for example, for treatment of cancer and metabolic diseases. Some potent and selective small-molecule HDAC11 inhibitors have already been reported, using not only a hydroxamic acid group as ZBGs like FT895, ZINC000028464438, and 5a but also an alkylated hydrazide group like SIS17 and B6 and an α, ß-epoxiketone like TD034 (Figure 1B).^19,38–42^ In the HDAC11-specific inhibitors TD034, SIS17, and B6, the ZBG is fused to a selectivity tail formed by a hydrophobic alkyl chain, which enhances interactions with HDAC11 and thereby induces selectivity of binding. Garcinol is a natural compound found to inhibit HDAC11 specifically using a ß-diketone as the potential ZBG.^43^

Taken together, there is a continued need for HDACi using alternative functional groups to target the catalytic Zn^2+^ ion and to allow the incorporation of additional groups mediating isoform selectivity. Therefore, the aim of this study was to develop potent and HDAC11-selective inhibitors featuring alternative ZBGs. The result of our research is the substrate-derived HDAC11-selective inhibitor **31**, which utilizes an α-amino amide structure as the ZBG combined with a selectivity tail and shows a *K_i_* value in the single-digit nanomolar range.

## Results and Discussion

### Synthesis and Inhibitory Potential of Acyl Peptide Derivatives

The aim of our study was the generation of selective and potent inhibitors based on the finding that the myristoylated 12-mer peptide H3K9­(Myr) (KQTARK­(Myr)­STGGWW) serves as a substrate of HDAC11 with a *K*_M_ value of 17.3 μM.^4,39^ We started with an N-terminally acetylated and C-terminally amidated 7-mer H3K9 peptide (Ac-TARKSTG-NH_2_) and generated derivatives with a C16 acyl chain linked to the lysine. The C16 acyl chain was selected as a selectivity tail because it was shown that HDAC11 deacylation activity was inhibited to a greater extent by free palmitic acid than by myristic or stearic acid.^10^ The acyl chain was modified at the C2 position adjacent to the scissile amide bond by introducing small moieties with free electron pairs to potentially enable them to form a ZBG in combination with the amide bond. Furthermore, the steric demand and the strength of certain incorporated Lewis bases were modified by substituting the heteroatom in order to enhance the interaction with the catalytic Zn^2+^ ion (Scheme 1A).

**Scheme 1:**
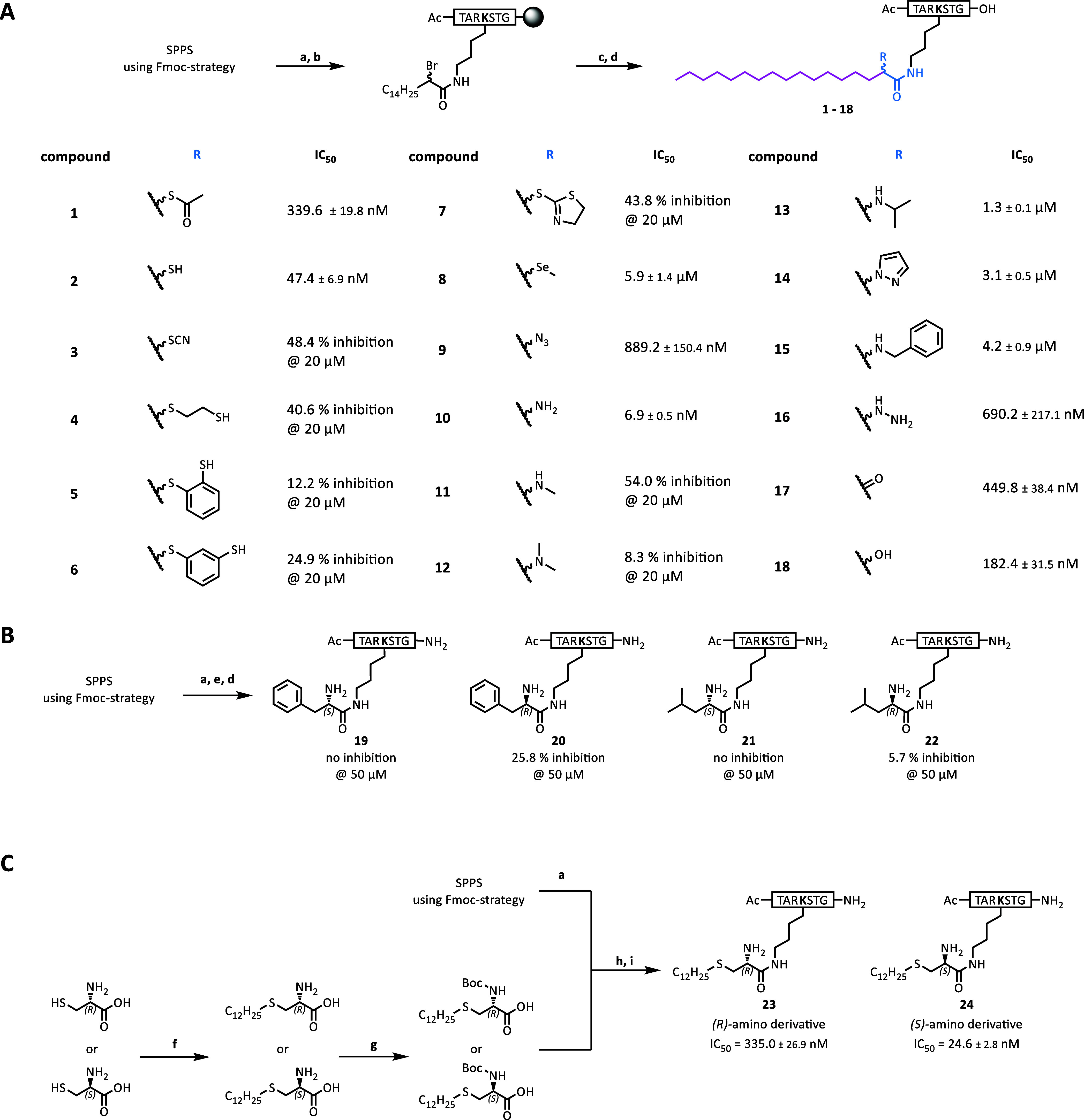
Synthesis and Inhibitory Potency of Compounds **1–24** ^a^

a (A) Compounds **1–18**. R represents moieties substituting one hydrogen in position C2 of the acyl chain. (B) Compounds **19–22**. (C) Compounds **23** and **24**. IC_50_ values of the resulting compounds are given. For weak inhibitors, the percent inhibition at an inhibitor concentration at 20 or 50 μM is given. All measurements were performed in triplicate. (a) Cleavage of the nosyl-protecting group using thiophenol, DBU in dry DMF, (b) 2-bromopalmitic acid, Oxyma/DIC in dry DMF, (c) introduction of *R*, (d) global deprotection using cleavage cocktails (supplementary material), (e) Boc-protected amino acid, HBTU, DIPEA in dry DMF, (f) 1,1,3,3,-tetramethylguanidine, 1-iododecane in dry MeOH/DMF, (g) Boc_2_O, DIPEA in DMF/DCM, (h) EDC/Oxyma in dry DCM/DMF, and (i) global deprotection using TFA, TIPS, 1-dodecylthiol, and water (90:2.5:2.5:5 (v/v)).

The peptide derivatives were synthesized using Fmoc-based solid-phase peptide synthesis (Scheme 1A). Fmoc-l-lysine was protected in the side chain with a nosyl group. The deprotected lysine side chain was acylated on the resin by using racemic 2-bromohexadecanoic acid and Oxyma/DIC as coupling reagents. Compounds bearing different substituents at the acyl C2 position were obtained by nucleophilic substitution of the bromine atom, followed, if necessary, by hydrolysis (**2**) or reductive amidation (**13** and **15**). Compound **17** was generated by transamination of **10**,^52^ and **18** was obtained by reduction of **17**. By this, we obtained compounds comprising (i) a long acyl moiety, designed to exhibit selectivity for HDAC11 over all other Zn^2+^-dependent HDACs; (ii) a ZBG situated in close proximity to the active site Zn^2+^ ion; and (iii) a peptidic backbone as the cap group for substrate-like binding to the HDAC11 active site.

In order to analyze the inhibitory efficacy of the compounds against HDAC11, they were subjected to an HPLC-based endpoint assay using the trifluoroacetylated peptide Abz-SRGGK­(TFA)­FFRR-NH_2_ as the substrate.^53^ Compounds that showed significant inhibition of detrifluoroacetylation were subjected to an IC_50_ determination against HDAC11, using the internally quenched TNFα-derived fatty-acylated peptide Ac-EALPK­(11-Abz-Aun)­KY­(3-NO_2_)­GG-NH_2_, with an aminoundecanoic acid (Aun) function modified with aminobenzoic acid (Abz) as the substrate in a continuous fluorescence-based assay.^45^

The compounds with the α-amino amide (**10**) and the α-mercaptoamide motif (**2**) as the ZBG exhibited the strongest inhibition of HDAC11, with IC_50_ values of 7 and 47 nM, respectively (Scheme 1A). It is noteworthy that our attempts to alter the nature of the ZBG present in **10** or **2**, whether through the addition of a π-system (**5**, **6**, and **14**), increasing the steric demand (**3**–**8** and **13**–**15**) or enhancing the basicity of the amino group (**11**–**14**), resulted in a reduction in inhibitory potency (Scheme 1A). This suggests the optimal size and spatial arrangement of the α-amino amide and α-mercaptoamide in the active site. However, all tested structures were still able to inhibit HDAC11, indicating that its active site is spacious and flexible enough to accommodate a variety of different groups.

### Scissile Amide Bond Resistance to Cleavage in Inhibitory Peptides

Because acylated H3K9 peptides are known to be substrates of HDAC11,^4,39^ it had to be analyzed if the presence of a substitution in close proximity to the hydrolyzable amide bond is sufficient to suppress the deacylation of the compound by HDAC11, thereby transforming a substrate into an inhibitor. Accordingly, 50 μM of the most promising compounds, specifically **2**, **10**, **17**, and **18**, were incubated in the presence of 10 or 100 nM of HDAC11 at 37 °C for 24 h, followed by UPLC–MS analysis. It was observed that HDAC11 accepted **17** and **18** as substrates but was not able to hydrolyze **2** and **10**, which were completely stable in aqueous solution at 37 °C for 24 h. Consequently, compounds **17** and **18** were excluded from further analyses.

### Stereochemical Requirements for Inhibition

Based on the finding that an amino group in the α-position of the fatty acid moiety exerts a strong inhibitory potency against HDAC11, we wanted to analyze the influence of the stereochemistry of the amino group on inhibition. For this approach, it was not possible to use the starting material 2-bromopalmitic acid because it was purchased as a racemate. Therefore, we modified our approach for initial analysis by replacing the palmitic acid derivative with enantiomerically pure α-amino acids with hydrophobic side chains. This allowed for the straightforward synthesis of stereochemically defined α-amino amides, in which the amino acid side chains mimic the hydrophobic acyl residue. Thus, **19**–**22** were synthesized by introducing either a d- or l-phenylalanine or a d- or l-leucine residue (Scheme 1B). It was observed that HDAC11 was inhibited by the d-amino acid derivatives, whereas the l-amino acid derivatives did not appear to exert any inhibitory effect on the activity of HDAC11 at 50 μM (Scheme 1B). Similarly, for HDAC8 inhibition by an α-amino ketone, the *R* chirality was described to be essential.^54,55^

Compounds combining a fatty acid-like selectivity tail with a defined stereochemistry of the α-amino amide ZBG were synthesized using d- or l-cysteine as the starting material (Scheme 1C). A selective *S*-alkylation of free d- or l-cysteine was performed using 1,1,3,3-tetramethylguanidine and 1-iodododecane to obtain the respective 2-amino-4-thia palmitic acid derivative.^56^ This was followed by the introduction of a Boc-protecting group at the free amino group to synthesize the stereochemically pure building block. The (*S*)- or (*R*)-2-(Boc-amino)-4-thia palmitic acid building block, respectively, was coupled to the lysine side chain of the 7-mer H3K9 peptide on a solid support. Both compounds were able to inhibit the defatty acylation activity of HDAC11 with IC_50_ values in the nanomolar range. The compound with the amino group in the S configuration (**24**) was found to be approximately 13 times more potent than the *R* analogue (**23**) (Scheme 1C).

### Synthesis and Inhibitory Potential of Nonpeptidic α-Amino Amide Derivatives

Next, we asked if the presence of the peptide as the inhibitor cap structure is required for efficient binding if a potent ZBG is used. To this end, the 7-mer H3K9 peptide was completely omitted. In order to retain the inhibitor linker moiety, pentylamine as lysine side chain mimic was coupled to the (*S*)-2-(Boc-amino)-4-thia palmitic acid building block, yielding compound **28**. Simultaneously, the relatively straightforward synthesis of the building block allowed us to synthesize the 2-amino-4-thia C10 to C14 acyl pentylamine derivatives **25**–**27** to examine the impact of the different lengths of the selectivity tail on inhibition (**25**–**28**; Figure 2A). Among these compounds, **28** showed the most potent inhibition against HDAC11 with an IC_50_ value in the low-nanomolar range, despite the absence of any possibilities of hydrogen bonds, except for the α-amino amide structure. The only slightly reduced IC_50_ value of **28** compared to that of **24** indicates only a minor contribution of the peptide moiety to affinity. Shortening of the acyl chain resulted in a considerable loss of inhibitory potency, as indicated by the increased IC_50_ values for **25**–**27** (Figure 2B).

**Figure 2:**
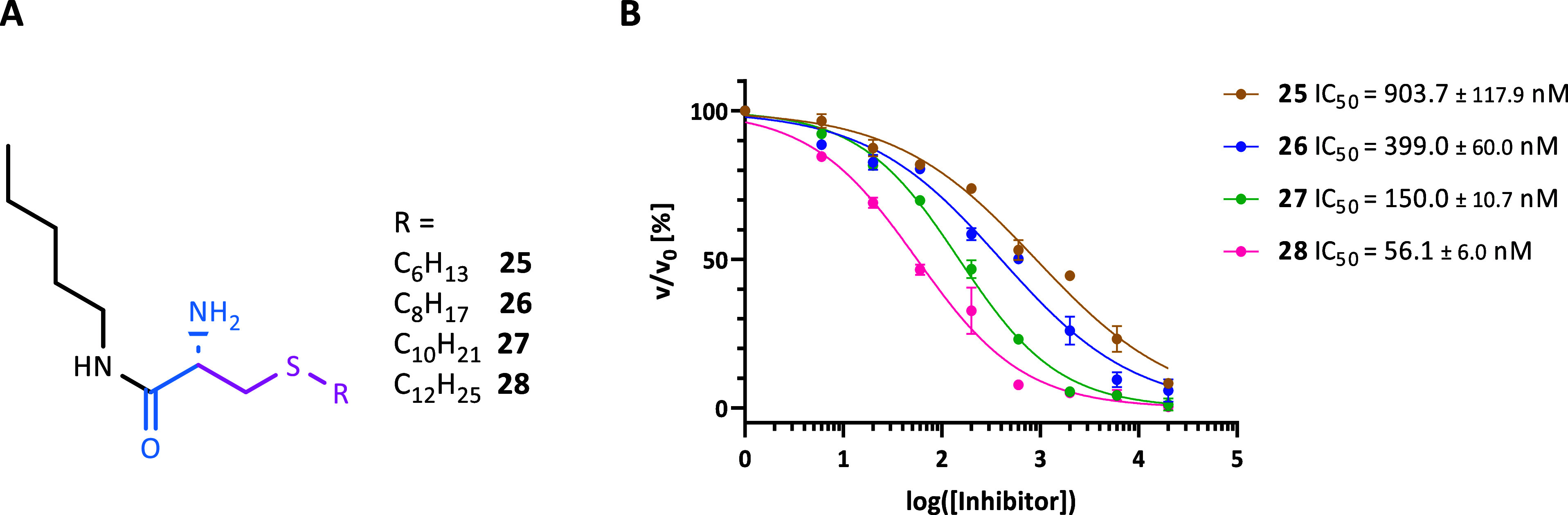
(A) Structures of acylated pentylamine derivatives **25**–**28**. (B) Dose–response curves for **25**–**28** against HDAC11 and the resulting IC_50_ values. All measurements were performed in triplicate.

Also, in the case of HDAC11 inhibition by free fatty acids, preferential recognition of longer alkyl chains and an affinity decrease with decreasing acyl group length were already observed.^10^ Similarly, the importance of the alkyl chain length for HDAC11 inhibition was demonstrated for the SIS compounds, where a hydrazide ZBG is combined with alkyl chains of different lengths.^39^

Taken together, the finding that **28** showed potent inhibition against HDAC11 indicates that the presence of an effective ZBG in combination with a palmitoyl-like residue suffices for high-affinity binding to HDAC11.

### Synthesis and Evaluation of Optimized α-Amino Amide Derivatives

In order to re-establish the possibility of stronger interactions, such as π–π interactions or hydrogen bonds with the enzyme, three different cap/linker groups with different functionalities were introduced, yielding compounds **29**–**31** (Scheme 2). The synthesis of **29** was started with the coupling of propargylamine to the (*S*)-2-(Boc-amino)-4-thia palmitic acid building block. Subsequently, the methyl azide was synthesized *in situ* prior to the click reaction by the addition of sodium azide and methyl iodide, together with copper­(II)­sulfate, ascorbic acid, and sodium carbonate. The synthesis of **30** was performed by coupling isoindoline to the (*S*)-2-(Boc-amino)-4-thia palmitic acid building block. For the synthesis of **31**, the diketopiperazine (DKP) cap group had to be prepared by in-solution cyclization of the dipeptide Fmoc-Lys­(Fmoc)-Gln-OMe. Fmoc-cleavage was performed using 20% piperidine in DMF. Subsequently, the DKP was coupled to FMP resin utilizing the free lysine side chain and NaBH­(OAc)_3_. The coupling of the (*S*)-2-(Boc-amino)-4-thia palmitic acid building block on the solid support was followed by global deprotection using a cleavage cocktail in TFA at room temperature (Scheme 2).

**Scheme 2:**
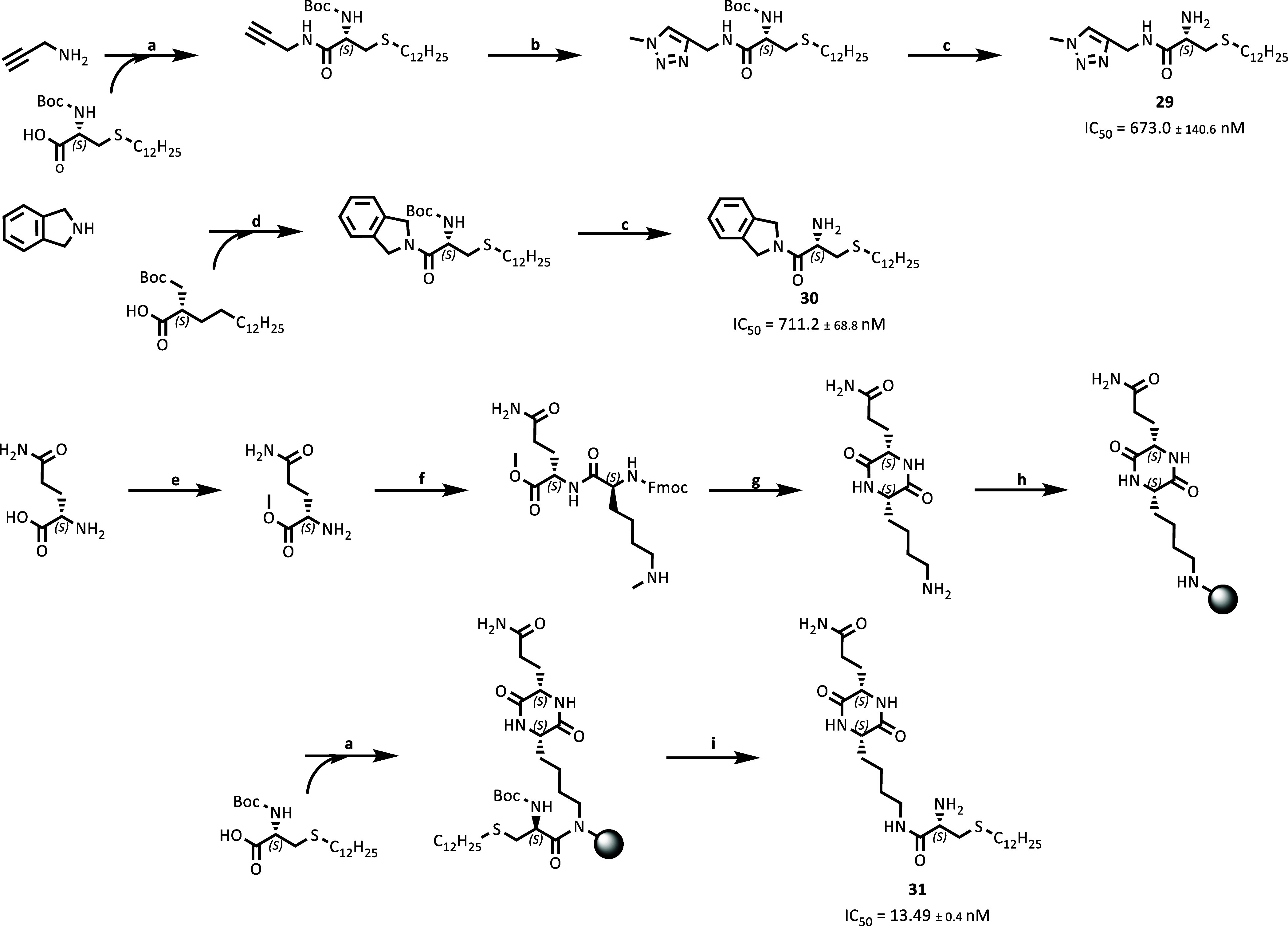
Synthesis of Compounds **29**–**31** ^a^

a (a) EDC/Oxyma in dry DMF, (b) CuSO_4_, ascorbic acid, Na_2_CO_3_, MeI, NaN_3_ in H_2_O/DMF (1:4 (v/v)), (c) TFA, (d) HATU/DIPEA in dry DMF, (e) SOCl_2_ in dry MeOH, (f) Fmoc-l-Lys­(Fmoc)–OH, Oxyma/EDC in dry DMF, (g) 20% (v/v) piperidine in DMF, (h) NaBH­(OAc)_3_, collidine, FMP resin, dry DMF, (i) global deprotection using TFA, TIPS, 1-dodecylthiol, and water (90:2.5:2.5:5 (v/v)). IC_50_ values against HDAC11 are shown. All measurements were performed in triplicate.

The heterocycles introduced to yield **29** and **30** resulted in a reduced inhibitory potency with IC_50_ values increased more than 10-fold compared to **28**. In contrast, the incorporation of the DKP cap group, which can be considered to mimic a peptide backbone,^57^ enabling the formation of hydrogen bonds in close proximity to the active site of HDAC11, increased the inhibitory potency compared to **28**. The IC_50_ value of **31** was determined to be 13.5 nM. The *K_i_* value of **31** was determined by fitting the data to the Morrison equation for tight binding to be 2.2 nM (Figure 3A). Compound **31** was confirmed to be a reversible inhibitor of HDAC11 using a jump dilution protocol, in which a preincubated complex of HDAC11 and **31** was diluted 100-fold, and recovery of the enzymatic activity of HDAC11 activity was obtained (Figure 3B).

**Figure 3:**
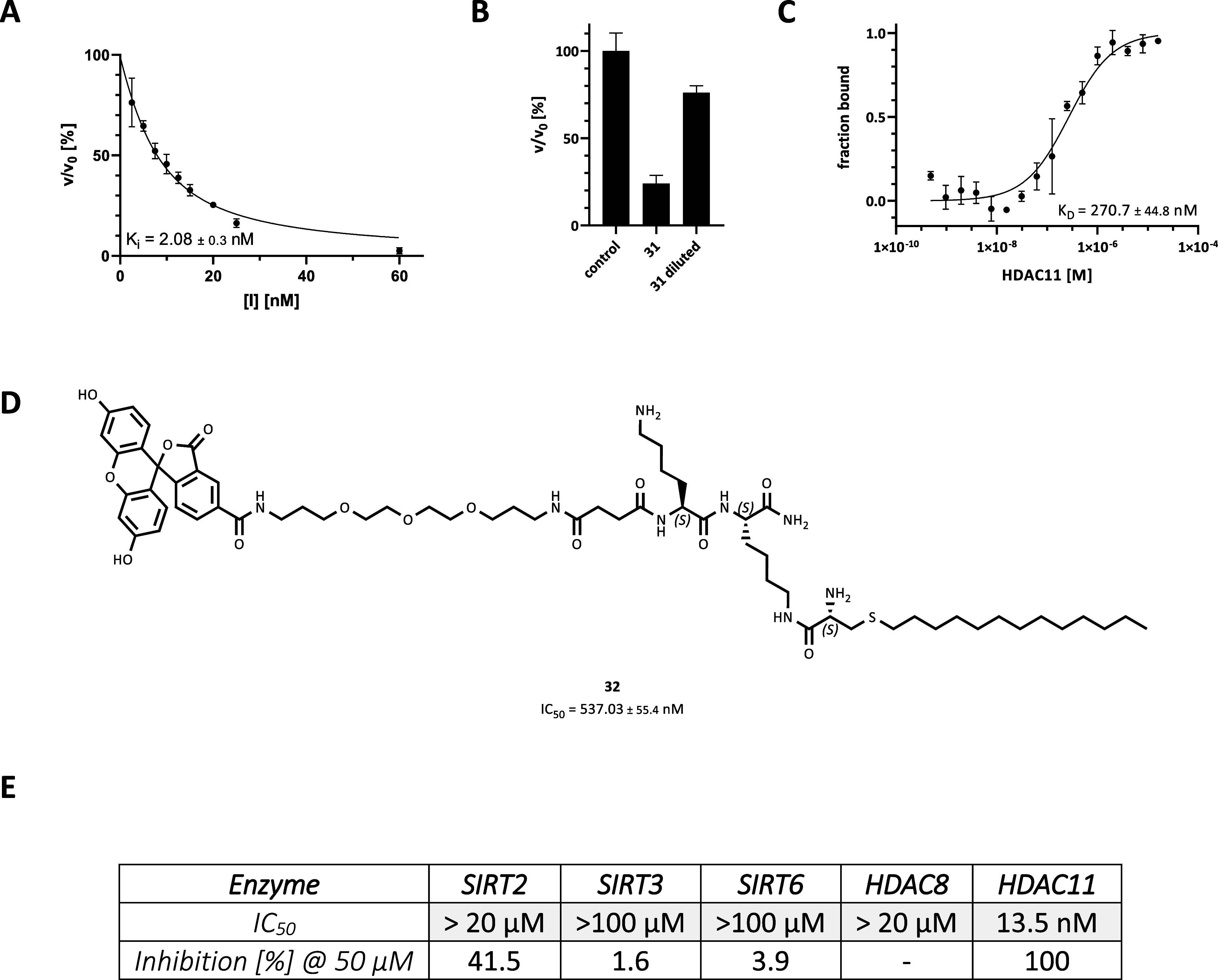
(A) *K_i_* value was determined for **31** using 5 nM HDAC11 and 20 μM of substrate Ac-EALPK­(11-Abz-Aun)­KY­(3-NO_2_)­GG-NH_2_.^45^ (B) Reversible inhibition of HDAC11 activity after jump dilution (HDAC11 = 5 nM, **31** = 270 nM, substrate = 20 μM (Ac-EALPK­(11-Abz-Aun)­KY­(3-NO_2_)­GG-NH_2_). (C) Determination of the *K*_D_ value using fluorescently labeled peptide derivative **32** (20 nM) and varying concentrations of HDAC11. (D) Structure of **32** combining fluorescein and the (*S*)-2-(amino)-4-thia palmitoyl moiety via a TTDS linker and a lysine dipeptide to analyze its binding to HDAC11 by MST. (E) Comparison of the inhibitory potency of **31** on defatty-acylases. All measurements were performed in triplicate.

Interestingly, an α-amino amide ZBG, in combination with a fatty acid-like selectivity tail, is sufficient to yield inhibitor potencies in the nanomolar range (**25**–**28**). Introduction of a cap/linker group can improve affinity (**31**) or reduce affinity (**29**, **30**); however, even very large substituents in the cap position do not prevent inhibition. This was shown by the analysis of **32**, in which a lysine dipeptide, a TTDS linker unit, and fluorescein were coupled to the (*S*)-2-(Boc-amino)-4-thia palmitic acid unit, which resulted in an IC_50_ value of 537 nM (Figure 3D). The fluorophore incorporated in compound **32** allowed us to analyze the binding of a fatty acyl α-amino amide to HDAC11 by microscale thermophoresis (MST) measurements. The MST-derived binding curve revealed a *K*_D_ value of 270 nM (Figure 3C).

Based on these data, **31** forms a highly potent inhibitor for HDAC11. This raises the question of the selectivity of **31** toward HDAC11 in comparison to HDAC8, another Zn^2+^-dependent defatty-acylase, and to NAD^+^-dependent class III defatty-acylases. As examples, SIRT2, SIRT3, and SIRT6 containing a structurally verified fatty acyl lysine binding pocket were chosen.^8,9,58^ Isozymes incapable of deacylating fatty acids were omitted in the analysis because their enzymatic properties imply the lack of an extended internal cavity in the active site to harbor the selectivity tail of **31**. SIRT2, 3, and 6, as well as HDAC8, exhibited minimal susceptibility to inhibition by **31** (Figure 3E). SIRT2 showed inhibition of about 41% in the presence of 50 μM **31**. Based on these findings, it can be concluded that **31** exhibits a high degree of selectivity for HDAC11, with a selectivity ratio >10,000 for HDAC11 compared to other defatty-acylases. Compound stability tests in human serum and HEK293 cell lysate indicated the presence of at least 20% of compound **31** after 6 h. Additionally, compound **31** showed uptake by HEK293 cells (Supporting Information H).

### Predicted Binding Mode of Compound **31**

The binding mode of compound **31** was studied by molecular docking using an AlphaFold2 model of HDAC11 previously validated and utilized to study the binding mode of HDAC11 inhibitors, as well as to discover novel and selective inhibitors^41,42,59^ (Supporting Information G). In the obtained docking pose, the long alkyl chain of **31** was suitably accommodated in the foot pocket of HDAC11 along loop 3 and loop 7 while forming hydrophobic interactions with Phe37, Trp42, Val45, Val137, Phe141, Phe152, and Cys153. (Figure 4A). One NH group of the DKP capping group formed a hydrogen bond with Glu94. **31** showed a bidentate chelation of the Zn^2+^ ion through the amino and carbonyl groups of the α-amino amide moiety. An α-amino amide-based inhibitor of HDAC8 with nanomolar affinities has already been described (Figure 4B).^54,55^

**Figure 4:**
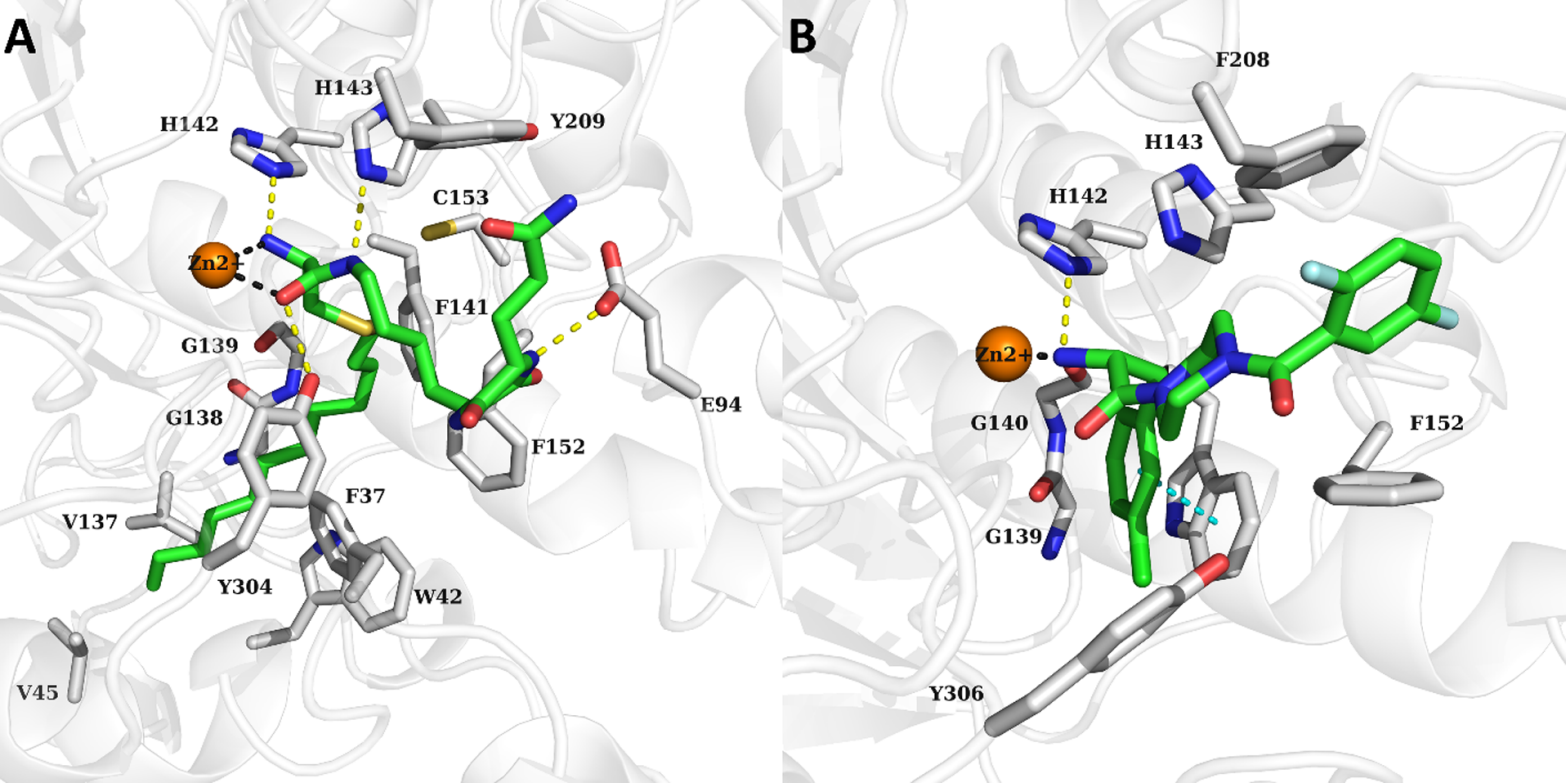
(A) Docking pose of compound **31** in the optimized HDAC11 AlphaFold2 model. The protein is demonstrated as a white ribbon. (B) Binding mode of an α-amino amide-based inhibitor cocrystallized with HDAC8 (PDB ID: 3SFF).^54^ The binding site residues are displayed as gray sticks, the ligand as green sticks, and the Zn^2+^ ion as orange sphere. The coordination bonds to the zinc ion are displayed as gray dashed lines, the hydrogen bonds as yellow dashed lines, and the π–π interactions as cyan colored dashed lines.

### Effect of Compound **31** on the Global Fatty-Acylation Level in Cells

Next, we analyzed how treatment of living cells with **31** influences the protein lysine fatty-acylation level by a click chemistry-based metabolic labeling method.^60^ First, HEK293 cells grown in the presence or absence of the specific HDAC11 inhibitor **31** were metabolically labeled with the alkynyl-analogue of myristic acid, Alk-14, as a chemical reporter for the specific detection of proteins becoming fatty-acylated in cells. Hydroxylamine was used to remove cysteine fatty acylation. Subsequently, a click chemistry reaction between the alkynyl-labeled proteins and Cy3-azide was performed to allow analysis of the modified proteins by in-gel fluorescence scanning (Figure 5A). Fluorescence analysis of total cell lysates revealed a number of labeled proteins. Coincubation of the HEK293 cells with the HDAC11 inhibitor **31** moderately increased the fluorescence signal, indicating an increase in protein acylation (Figure 5B). Similarly, HDAC11 knockdown was already described to lead to an increased level of fatty-acylated proteins in cells, as indicated by an increase in in-gel fluorescence.^4^ However, other known deacylases, such as the sirtuins SIRT1–3, SIRT5 and SIRT6, as well as HDAC8, may reduce the observable effect by compensatory mechanisms. Interestingly, previous studies showed that treatment with the pan-HDAC inhibitor SAHA led to a decrease in global protein fatty acylation, likely due to a reduced number of free lysine amino groups available for acylation as a result of SAHA-induced hyperacetylation.^61^ Taken this into account, the increase in protein acylation in the presence of **31** concomitantly emphasizes the specificity of the effect of the compound on deacylation, leaving deacetylation uninfluenced.

**Figure 5:**
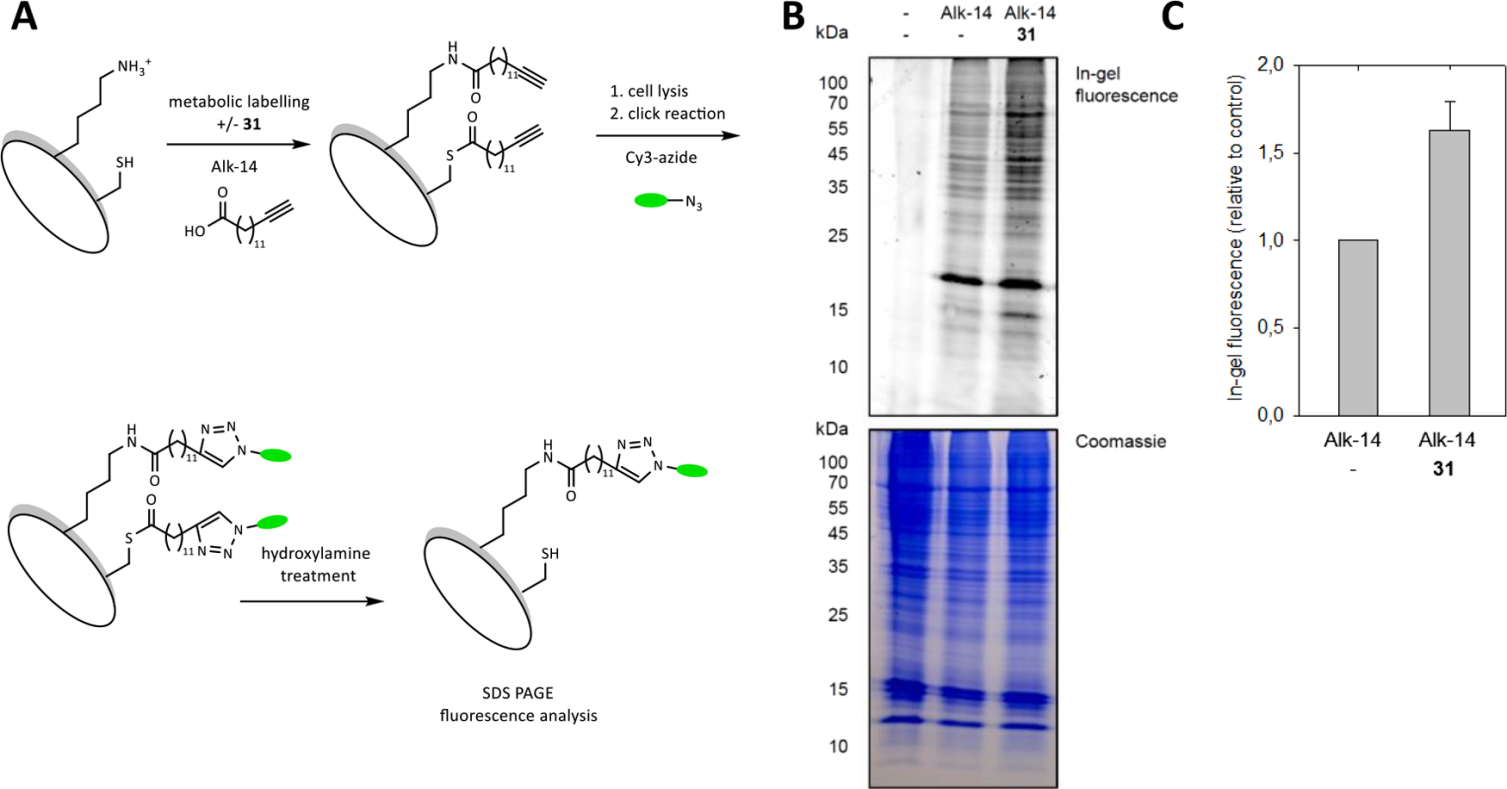
(A) Scheme for metabolic labeling utilizing Alk-14 as a chemical reporter for the specific detection of proteins becoming fatty-acylated in cells, followed by a click chemistry reaction with Cy3-azide as detection tag. (B) Profile of proteins fatty-acylated by the alkynyl-analogue of myristic acid, Alk-14, in HEK293 cells. Fatty-acylated proteins from the mammalian cell line HEK293 were metabolically labeled with DMSO (−) or Alk-14 (25 μM) in the presence or absence of the HDAC11 inhibitor **31** (20 μM) and analyzed using Cy3-azide via click chemistry, followed by in-gel fluorescence scanning. Comparable levels of protein loading were demonstrated by Coomassie blue staining of the gel. (C) Relative intensity of in-gel fluorescence was quantified by ImageQuant software. The experiments were performed in biological triplicate.

## Conclusions

In summary, we have developed a highly potent HDAC11-specific inhibitor by modification of a substrate of HDAC11. Compound **31** inhibits HDAC11 deacylase activity at low nanomolar concentrations *in vitro* *.* Concomitantly, the application of **31** on living cells enhances fatty acylation of proteins, suggesting the inhibition of HDAC11 inside cells. Molecular docking studies suggest interactions of the foot pocket of HDAC11 with the long alkyl chain of **31** as the origin of selectivity.

## Materials and Methods

### Materials

HDACs were expressed and purified as described previously.^10^ All chemicals were purchased from Sigma-Aldrich, unless otherwise indicated. *N*,*N*-dimethylformamide (DMF), piperidine, ethyl­(hydroxyamino)­cyanoacetate (OxymaPure), pentafluorophenol, and Rink amide MBHA were purchased from Iris Biotech. The 9-fluorenylmethoxy-carbonyl-(Fmoc)-protected amino acid derivatives were purchased from Merck.

### Synthesis of the Inhibitors

The peptide derivative Ac-TARK­(Ns)­STG-NH_2_ was synthesized on Rink amide MBHA resin (Novabiochem) using Fmoc-based solid-phase peptide synthesis (SPPS) with an automated microwave peptide synthesizer Liberty Blue (CEM Corporation). Subsequent to the SPPS, the nosyl-protecting group was cleaved, and the lysine side chain was modified at the solid support. The precise procedures for each compound can be found in the Supporting Information.

The diketopiperazine structure was generated by cyclization and modification on solid support. The exact procedures for each compound can be found in the Supporting Information.

UPLC–MS analysis was performed by using a Waters Acquity UPLC–MS system with a Waters Acquity UPLC–MS-BEH C18 column (1.7 μM, 2.1 × 50 mm; 30 Å). Data analysis was performed by using Waters MassLynx software.

### *In Vitro* HDAC Inhibition Assays

Inhibition of the enzymatic activity of HDACs was performed continuously for HDAC8, HDAC11, and SIRT2 and discontinuously for SIRT2, 3, and 6 using adequate substrates as described previously.^41,58,62^ Experimental details are given in the Supporting Information.

### Substrate Assay

The peptides were incubated at 50 μM, together with 10 or 100 nM HDAC11 in HDAC11 buffer for 24 h at 37 °C. The solution was then analyzed using UPLC–MS.

### Microscale Thermophoresis

Microscale thermophoresis (MST) was performed using a Monolith NT.115 (Nanotemper) with standard capillaries (MO-K022). The reaction solution contained a dilution series (1:1) of 16 different concentrations of HDAC11 (0.48 nM to 16 μM) and 20 nM **32** in MST buffer (20 mM MOPS buffer (pH 7.4 adjusted with NaOH), 0.2 mg/mL BSA, and 70 μM TCEP) and 5% (v/v) DMSO. After 5 min of incubation, the samples were loaded into capillaries and measured at 25 °C with a Filter Nano-BLUE at an excitation power of 20% and an MST power of 40%. The resulting data were fitted by MO.Affinity Analysis software (Nanotemper) via MST curves and fluorescence signals.

### Docking

Molecular docking was performed as described previously.^41^ Details are given in the Supporting Information.

### Metabolic Labeling, Copper-Catalyzed Azide–Alkyne Cycloaddition (CuAAC) Reactions, and Fluorescence Detection

Metabolic labeling of HEK293 cells with Alk-14­(13-tetradecynoic acid) in the presence or absence of **31** (20 μM), CuAAC reactions, and detection of the labeled proteins by in-gel fluorescence scanning were performed, as described in the literature.^63^ Experimental details are given in the Supporting Information.

## Supplementary Material



## Notes

Additional experimental details, UPLC–MS data, and dose–response curves for inhibitor measurements (PDF)
