# Supplementary material for: Substrate-Guided Development of HDAC11-Selective Inhibitors Featuring α‑Amino Amide Zinc-Binding Groups
Source: ACS Omega. 2025 Oct 14;10(42):50577–87. doi: 10.1021/acsomega.5c08195 (PMC12573178; doi:10.1021/acsomega.5c08195)
Supplement: Supplementary file 1 [file ao5c08195_si_001.pdf]

Substrate-Guided Development of HDAC11-Selective Inhibitors Featuring α-Amino Amide Zinc-Binding Groups

*Sebastian Hilscher [a,b], Marat Meleshin [a], Fady Baselious [b], Cyril Barinka [c], Wolfgang Sippl [b], Mike Schutkowski [a] , and Cordelia Schiene-Fischer [a]**

[a] Department of Enzymology, Charles Tanford Protein Center, Institute of Biochemistry and Biotechnology, Martin-Luther-University Halle-Wittenberg, 06120 Halle (Saale), Germany

[b] Department of Medicinal Chemistry, Institute of Pharmacy, Martin-Luther-University Halle-Wittenberg, 06120 Halle (Saale), Germany

[c] Institute of Biotechnology of the Czech Academy of Sciences, BIOCEV, Prumyslova 595, 252 50 Vestec, Czech Republic

* Corresponding author:
cordelia.schiene-fischer@biochemtech.uni-halle.de

KEYWORDS

Histone deacetylase 11, zinc-binding groups, HDAC11 inhibitors, HDAC inhibitors, peptide derivatives, cellular activity

A. HDAC and SIRT deacylase activity assays....S3
B. Metabolic labeling, CuAAC reactions and fluorescence detection....S4
C. General synthetic procedures....S5
D. Dose-response curves....S16
E. UPLC-MS spectra of the compounds with UV/VIS trace....S18
F. UPLC-MS Data of the substrate test....S35
G. Docking of **31** and predicted binding mode....S37
H. Stability and permeability of **31** S38

A. HDAC and SIRT deacylase activity assays

Continuous *in vitro* HDAC inhibition assays

The inhibitors were tested using 384-well plates (GreinerONe). After 5 min of incubation of the inhibitors together with the respective enzymes (HDAC8 = 2 nM, HDAC11 = 20 nM, SIRT2 = 1 nM), the reactions were always started by the addition of substrate for the Zn^{2+}-dependent HDAC and by the addition of NAD^+ for SIRT2. The reactions were performed in either HDAC8 buffer (20 mM HEPES, 140 mM NaCl, 10 mM $MgCl_2$, 1 mM tris(2-carboxyethyl)phosphine (TCEP), and 0.2 mg/ml BSA, pH 7.4 adjusted with NaOH), HDAC11 buffer (20 mM HEPES, 70 µM TCEP and 2 mg/ml BSA, pH 7.4 adjusted with NaOH) or SIRT buffer (20 mM Tris, 150 mM NaCl, 5 mM $MgCl_2$ and 2 mg/ml BSA pH 7.8 adjusted with HCl), respectively. The thiotrifluoracetylated substrate Abz-SRGGK(thio-TFA)FFRR-NH_2 (50 µM) was used for HDAC8, the substrate Ac-EALPK(11-Abz-Aun)KY(3-NO_2)GG-NH_2 (20 µM), where 11-Abz-Aun is *N*-(2-aminobenzoyl)-11-aminoundecanoyl, was used for HDAC11 and Ac-EALPKK(Myr)T-C(Fluorescein)-G-NH_2 (40 nM) was used for SIRT2 [1-3].
The fluorescence increase was followed for 1 h with two reads per min using a PerkinElmer Envision 2104 multilabel plate reader. The excitation wavelength for HDAC8 and 11 was 320 ± 8 nm and the emission wavelength 430 ± 8 nm, for SIRT2 an excitation wavelength of 485 ± 14 nm and an emission wavelength of 535 ± 25 nm was used. Positive (enzyme, substrate, DMSO, and buffer) and negative (substrate, DMSO, and Buffer) controls were included in every measurement and were set as 100% and 0%, respectively. The measured values were normalized accordingly. All tests were performed in triplicates. The data were fitted using the GraphPad Prism8 software and kinetic values calculated through non-linear regression analysis.

Discontinuous *in vitro* inhibition assays

Initial analysis of inhibition was performed discontinuously in the presence of 20 µM inhibitor using 20 nM HDAC11 and 50 µM Abz-SRGGK(TFA)FFRR-NH_2 in HDAC11 buffer containing 5% (*v/v*) DMSO at 30°C. Reactions were stopped after 20 min by adding a constant volume of reaction mixture to stop solution (5 % acetonitrile (ACN) (*v/v*) and 1 % trifluoroacetic acid (TFA) (*v/v*) in dd. H_2O). The samples were analysed using HPLC-UV/VIS at λ_{Abs} = 220 nm, λ_{Ex} = 320 nm und λ_{Em} = 420 nm.

All reactions of the sirtuins were performed in SIRT buffer. To investigate the inhibition of the demyristoylation catalysed by SIRT2, 3 and 6 a modified sequence derived from TNFα (15 – 23) was used (Ac-EALPKK(X)Y(3-NO_2)GG-NH_2). All reactions were performed at 37 °C. Each reaction is composed of NAD^+ (500 µM for SIRT2 and 3 and 1 mM for SIRT6), along with a substrate concentration of 5 µM (TNFα-Myr), and 5% (*v/v*) DMSO. The reactions were started by the addition of enzyme (SIRT2: 100 nM, SIRT3: 200 nM, SIRT6: 500 nM). The reactions of SIRT2 and 3 were stopped after 10 min and SIRT6 reactions were stopped after 30 min by adding a constant volume of reaction mixture to stop solution. The samples were analysed using HPLC-UV/VIS at λ_{Abs} = 360 nm.

All discontinuous reactions were analysed using the HPLC system 1100 from Agilent with a quaternary pump, a well-plate autosampler and a variable absorption and fluorescence detector. A Phenomenex Kinetex XB C18 2.6 µm (3.0 mm x 50 mm) column was used. The flow rate was 0.6 ml min^{-1} and the mobile phase (ACN : H_2O) contained 0.1 % (*v/v*) TFA. Positive (enzyme, substrate, DMSO, and buffer) and negative (substrate, DMSO, and buffer) controls were included in every measurement and set as 100% and 0%, respectively. The measured values were normalized accordingly. All tests were done in triplicates.

Jump-dilution experiments

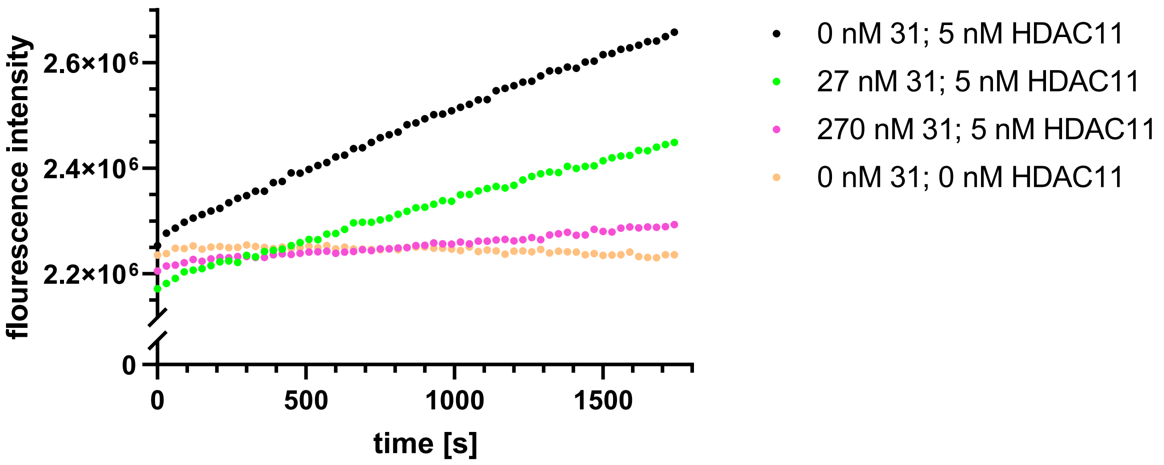

*Figure S **1**: An investigation was conducted into the reversible inhibition of HDAC11 activity by **31**, using a jump dilution experiment. Therefore, a preincubation of HDAC11 at 50 nM and **31** at 270 nM was conducted for 10 minutes. The mixture was diluted 1:100 by the addition of the substrate (Ac-EALPK(11-Abz-Aun)KY(3-NO_2)GG-NH_2) with a final concentration of 20 µM (green). As controls were constituted by a classic positive control (DMSO, enzyme, substrate; black) and a negative control (DMSO, buffer, substrate; orange). In order to demonstrate that HDAC11 is potently inhibited at a concentration that is tenfold greater than the K_i, a mixture comprising 270 nM **31** and 5 nM HDAC11 was also included (pink).*

B. Metabolic labeling, CuAAC reactions and fluorescence detection

HEK293 cells (1 × 10^6) were cultured in 2 ml of DMEM medium supplemented with 10 % FCS. For metabolic labeling cells were incubated with 25 µM of the chemical reporter Alk-14 (13-tetradecynoic acid) for 24 h in the presence or absence of the HDAC11 inhibitor (20 µM) or with DMSO as control. Cells were harvested, washed twice with PBS and lysed in lysis buffer (4 % SDS, 150 mM NaCl, 50 mM triethanolamine, pH 7.4, protease inhibitor cocktail (EDTA-free, Roche)). Insoluble cell debris was removed by centrifugation for 10 min. The supernatants were collected to yield total cell lysates. The protein concentrations were determined by BCA assay.

Analysis of metabolic labeling by the click chemistry approach was performed according to Charron et al, 2008. Briefly, 50 µg of cell lysates with a protein concentration of 1 mg/ml in lysis buffer were incubated with freshly premixed click chemistry mixture containing 100 µM Cy3-azide (Lumiprobe), 1 mM TCEP, 100 µM TBTA and 1 mM $CuSO_4$ at room temperature for 1 h. Hydroxylamine at a final concentration of 0.5 M was added and samples were heated for 7 min at 95°C to remove cysteine fatty acylation [4].

The reactions were stopped by addition of 1 ml ice-cold methanol, placed at -80 °C overnight and centrifuged at 13000g for 10 min at 4 °C to precipitate proteins. The protein pellets were resuspended in 50 µl of Laemmli buffer, heated for 5 min at 95 °C and subjected to 15% SDS-PAGE. The gel was analysed by in-gel fluorescence scanning using a Typhoon Trio+ imager (GE Healthcare). Coomassie staining of the gel was used as loading control. The experiment was performed in triplicate.

C. General synthetic procedures

The peptide derivative Ac-TARK(Ns)STG-NH_2 was synthesized on Rink amide MBHA resin (Novabiochem). Briefly, the peptides were synthesized using Fmoc-based solid-phase peptide synthesis (SPPS) with automated microwave peptide synthesizer Liberty Blue (CEM Corporation). The coupling of amino acids was performed with *N*,*N*′-diisopropylcarbodiimide (DIC)/ethyl cyanohydroxyiminoacetate (Oxyma) at 90 °C for 2 min. Fmoc deprotection was accomplished with 20 % (*v/v*) piperidine solution in *N*,*N*-dimethylformamide (DMF) at 90 °C for 1 min. The final *N*-terminal acetylation was done using a 1:2:7 mixture of acetic anhydride/*N*,*N*-diisopropylethylamine (DIPEA)/DMF (*v/v/v*) for 1 h at room temperature. The nosyl group was selectively removed using thiophenol and 1,8-diazabicyclo[5.4.0]undec-7-ene (DBU) in dry DMF. Following the resin washing step, racemic *2*-bromopalmitic acid was coupled using DIC/Oxyma in dry DMF. Subsequently, the respective ZBG was introduced on solid support. The modification of the cysteine was conducted using an alkyl halogenide and 1,1,3,3-tetramethylguanidine in MeOH/THF.

All washing steps were conducted using 5 x DMF and 4 x dichloromethane (DCM). All peptides were cleaved from the solid support using TFA (95 % *v/v*), unless otherwise stated. Following the removal of TFA *in vacuo*, the crude product was dissolved in water: ACN (1:1, *v/v*). All compounds were purified on the Shimadzu LC System with a Phenomenex Kinetex 5 µm XB-C18 (250 × 21.1 mm, 100 Å) column using different gradients of 0.1% TFA in H_2O (solvent A) and 0.1% TFA in acetonitrile (solvent B) solutions. UPLC-MS analysis of the fractions was performed using Waters Acquity UPLC-MS system (Milford, USA) with a Waters Acquity-UPLC-MS-BEH C18; 1.7 µM (2.1 × 50 mm; 30 Å) column. As the mobile phase, 0.1% formic acid in H_2O (solvent A) and 0.1% formic acid in acetonitrile (ACN; solvent B) solutions were used. A typical gradient from 95:5 (v/v) H_2O/ACN to 5:95 (v/v) H2O/ACN in 6 min was used for most of the runs. Data analysis was performed using Waters MassLynx 4.1 software. Product-containing fractions were combined and lyophilised. All compounds were obtained in a puritiy > 95 %.

Synthesis of the precursor for peptidic inhibitors

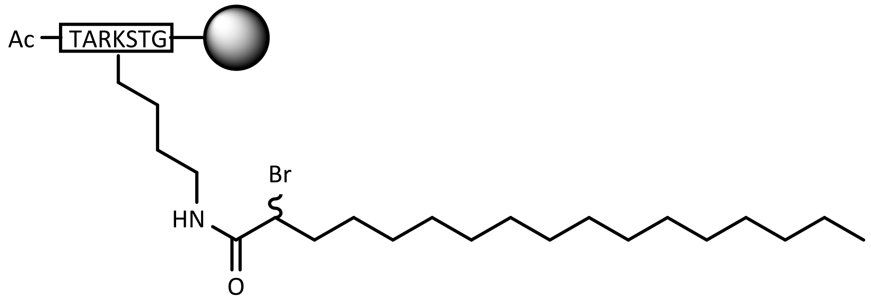

After selectively removing the nosyl protecting group of resin bound Ac-TARK(Ns)STG using 500 µl thiophenol and 700 µl DBU in 4 ml dry DMF, 2-bromopalmitic acid was coupled using 5 eq. together with 5 eq. of Oxyma and 10 eq. of DIC in dry DMF.

Synthesis of 1

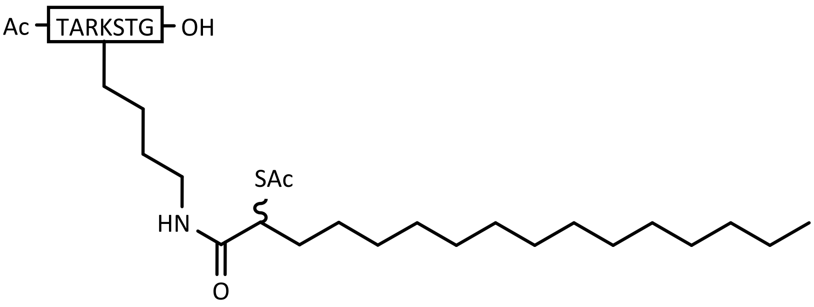

The precursor was shaken overnight together with 10 eq. of potassium thioacetate in dry DMF at room temperature. After washing the resin, the peptide was cleaved from the solid support and purified by HPLC using a gradient of B from 35 to 70 % in 45 min (t_R: 21.8 min).

Synthesis of 2

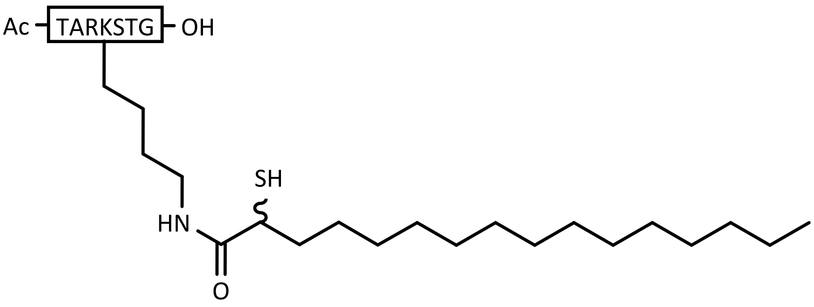

1 on solid support was shaken for a period of 10 minutes at room temperature with a solution of piperidine (20% *v/v*) in DMF. Following the washing of the resin, this process was repeated once. After washing the resin, the peptide was cleaved using a solution containing triisopropylsilane (TIPS) (2.5% *v/v*), 1-dodecanethiol (5% *v/v*), water (2.5% *v/v*) and TFA (90 % *v/v*) and purified by HPLC using a gradient of B from 30 to 65 % in 45 min (t_R: 25.7 min).

Synthesis of 3

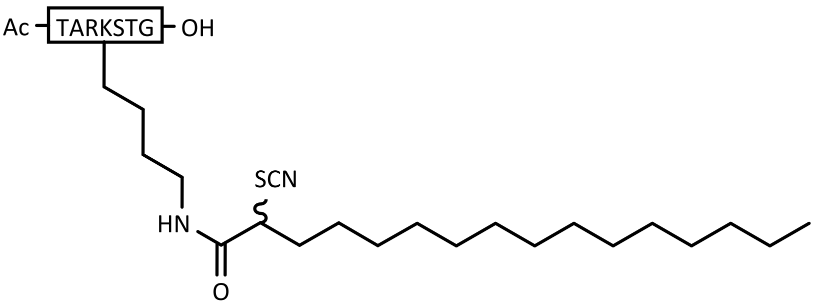

The precursor was cleaved from the resin, and following the removal of TFA, the crude product was dissolved in methanol together with 10 eq. of sodium thiocyanate [5]. Subsequently, the solution was shaken at 60 °C for 16 hours, after which the methanol was removed *in vacuo*. This procedure was repeated twice. Subsequently, methanol was removed *in vacuo*, the crude product was dissolved in a

solution of water and ACN (1:1 *v/v*) and purified by HPLC using a gradient of B from 30 to 60 % in 45 min (t_R: 26.1 min).

Synthesis of 4

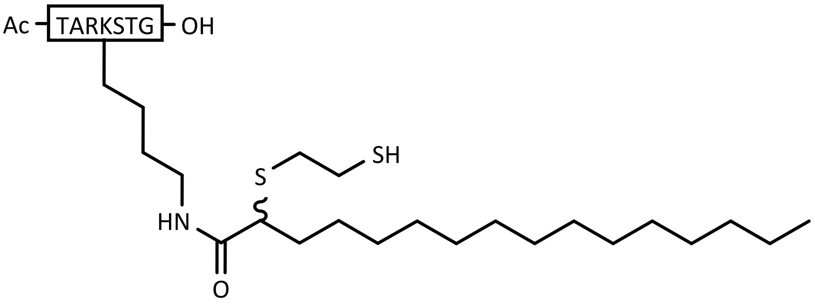

The precursor was cleaved from the resin, and following the removal of TFA, the crude product was dissolved in DMF together with 8 eq. of ethane-1,2-dithiol and 10 eq. of DIPEA. This solution was shaken for a period of two hours at 60 °C. The DMF was removed using an oil pump and the crude product was dissolved in a solution of water and ACN (1:1 *v/v*) together with 20 µl of an aqueous 10 mM TCEP solution, followed by purification by HPLC using a gradient of B from 35 to 70 % in 45 min (t_R: 24.4 min).

Synthesis of 5 and 6

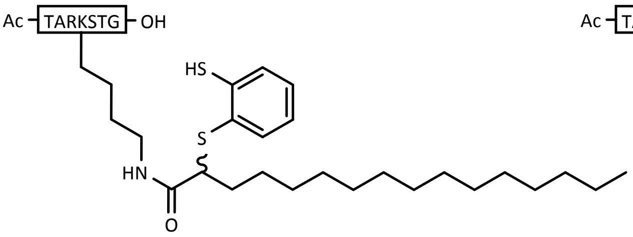

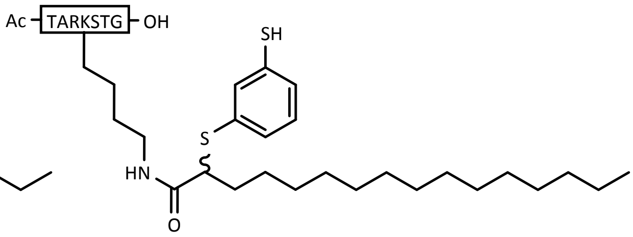

The precursor was cleaved from the resin, and following the removal of TFA, the crude product was dissolved in DMF together with 6 eq. benzene-1,2-dithiol or benzene-1,3-dithiol, respectively, and 10 eq. of DIPEA. This solution was shaken for 90 min at 60 °C. The DMF was removed using an oil pump and the crude product was dissolved in a solution of water and ACN (1:1 *v/v*) together with 10 µl acetic acid and 20 µl of an aqueous 10 mM TCEP solution, followed by purification by HPLC using a gradient of B from 30 to 75 % in 45 min (t_R: 26.8 min).

Synthesis of 7

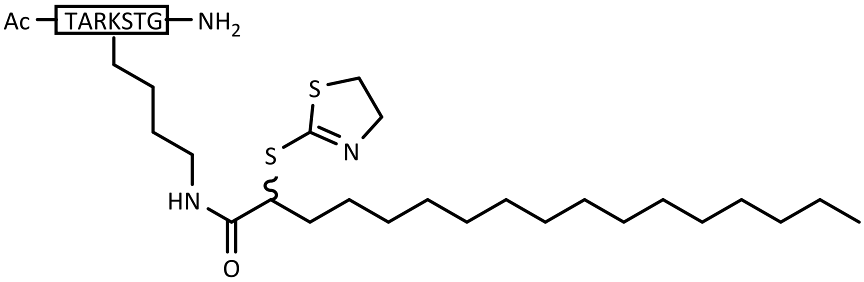

2 eq. of 2-thiazolin-2-thiol were preincubated together with 10 eq. of TCEP in a solution of DMF and water (95 : 5 *v/v*) at room temperature for 30 min. Subsequently, the solution was added to the precursor on solid support, and the reaction was shaken at room temperature overnight. Following the washing steps, the peptide was cleaved from the solid support for 45 min twice and then purified by HPLC using a gradient of B from 30 to 60 % in 45 min (t_R: 24.8 min).

Synthesis of 8

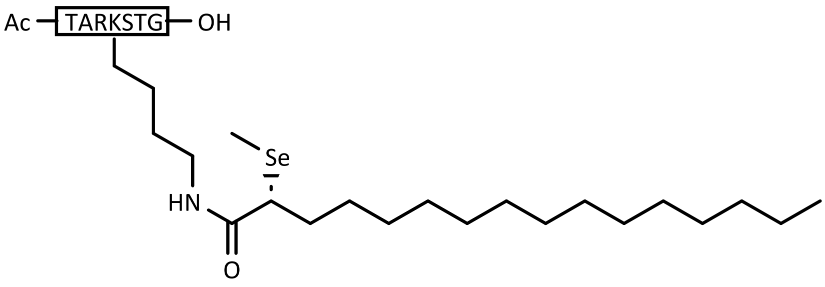

In order to obtain **8**, the 2-bromopalmitic acid was modified prior to coupling it to the lysine side chain [6]. Accordingly, 0.6 eq. of dimethyl diselenide were dissolved in anhydrous ethanol under an argon atmosphere and cooled to 0 °C. While stirring, 1.2 eq. of sodium cyanoborohydride were added twice, until the solution became clear. Subsequently, 1 eq. of 2-bromopalmitic acid was added, and the solution was stirred for 90 minutes at 0 °C. Afterwards, the solvent was removed by vacuum evaporation, and a concentrated citric acid solution was added to the residue and the mixture extracted three times using DCM. Following the removal of the DCM, crude 2-(methylselanyl)palmitic acid was obtained in a yield of 60 %. The modified palmitic acid was then coupled to the free lysine side chain on the resin using 3 eq. of the modified fatty acid, 3 eq. of *O*-(7-azabenzotriazol-1-yl)-*N,N,N',N'*-tetramethyluronium hexafluorophosphate (HATU) and 6 eq. of DIPEA in dry DMF. The reaction was shaken at room temperature for 60 minutes and this procedure was repeated once. After washing the resin, the peptide was cleaved from the solid support and purified by HPLC using a gradient of B from 25 to 60 % in 45 min (t_R: 24.4 min).

Synthesis of 9

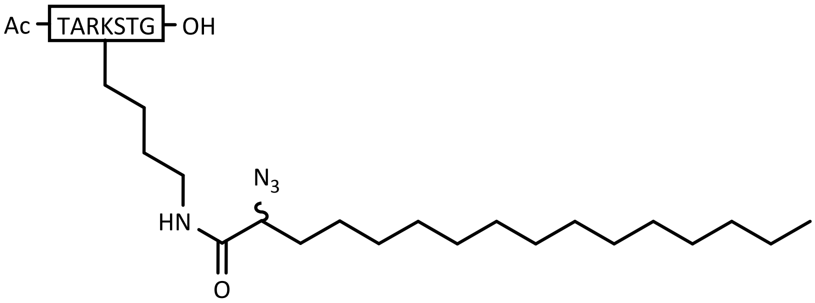

The precursor was shaken overnight together with 3 eq. of sodium azide in dry DMF at room temperature. After washing the resin, the peptide was cleaved from the solid support and purified by HPLC using a gradient of B from 25 to 60 % in 45 min (t_R: 24.7 min).

Synthesis of 10

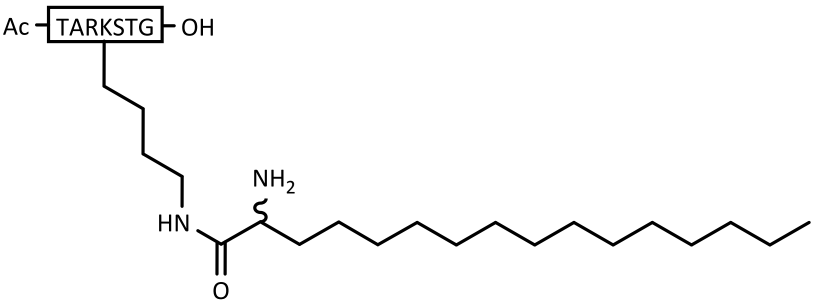

2-aminopalmitic acid was bought from Merck. Fmoc introduction at the 2-aminopalmitic acid was made as described by Koppitz et al. [7]. Afterwards, 3 eq. of the modified fatty acid was coupled to the free lysine side chain on the resin using 3 eq. of HATU and 6 eq. of DIPEA in dry DMF. The reaction was shaken at room temperature for 60 minutes and this procedure was repeated once. After washing the resin, the peptide was cleaved from the solid support and purified by HPLC using a gradient of B from 25 to 60 % in 45 min (t_R: 21.6 min).

Synthesis of 11

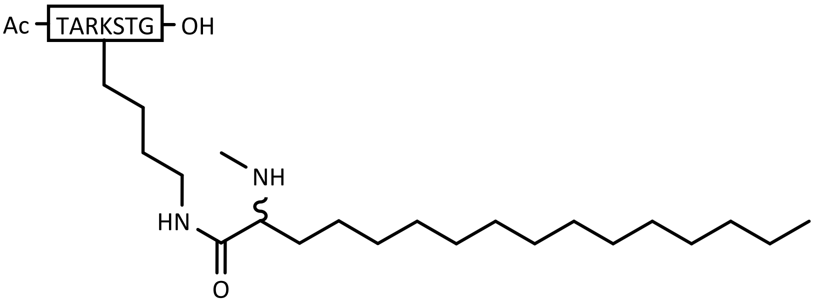

The precursor was shaken overnight together with a solution containing 40 % aqueous methylamine and DMF (1 : 4 *v/v*) at room temperature. After washing the resin, the peptide was cleaved from the solid support and purified by HPLC using a gradient of B from 25 to 60 % in 45 min (t_R: 20.4 min).

Synthesis of 12

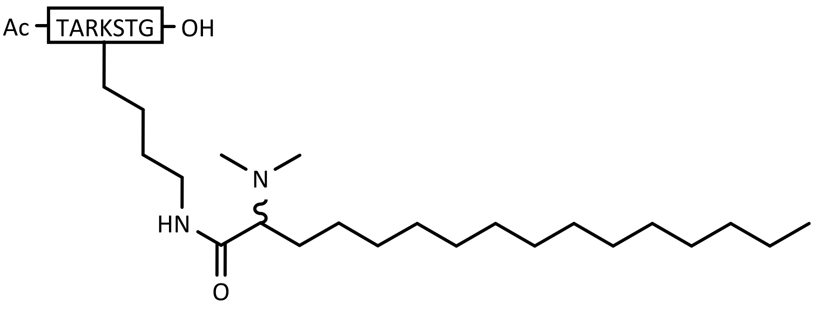

The precursor was shaken overnight together with 10 eq. of dimethylamine and 5 eq. of DIPEA in a solution of water and DMF (1 : 3 *v/v*) at room temperature. This procedure was repeated once, after which the resin was washed and the peptide was cleaved from the solid support and purified by HPLC using a gradient of B from 30 to 65 % in 45 min (t_R: 21.1 min).

Synthesis of 13

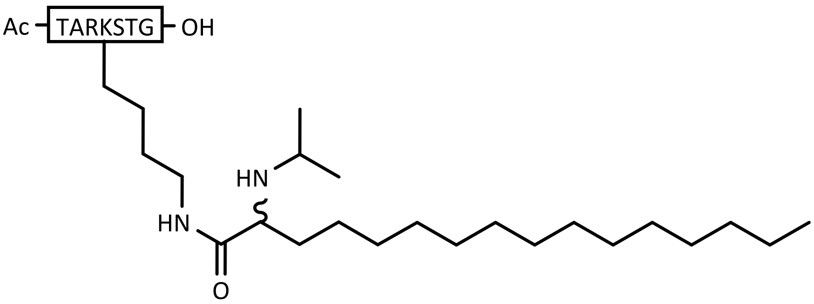

10 on solid support was shaken with a solution of 10 eq. of acetone and 2.5 % (*v/v*) acetic acid in dry DMF for 10 min. Then, a solution of 10 eq. of sodium cyanoborohydride in DMF was added to the resin and it was shaken for 24 hours at room temperature. This procedure was repeated once, after which the resin was washed and the peptide was cleaved from the solid support and purified by HPLC using a gradient of B from 25 to 60 % in 45 min (t_R: 19.3 min).

Synthesis of 14

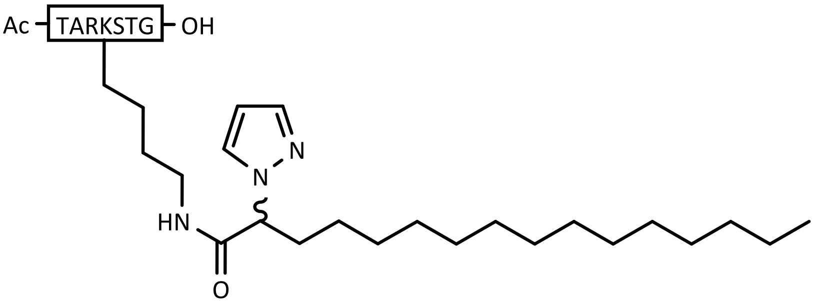

The precursor was shaken overnight together with 10 eq. of pyrazole and 5 eq. of DBU in DMF at 50 °C. This procedure was repeated once, after which the resin was washed and the peptide was cleaved from the solid support and purified by HPLC using a gradient of B from 25 to 60 % in 45 min (t_R: 21.7 min).

Synthesis of 15

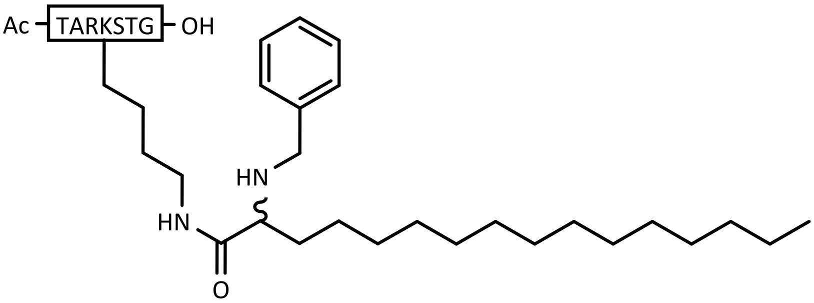

10 on solid support was shaken in a solution of 10 eq. of benzyaldehyde and 2.5 % (*v/v*) acetic acid in dry DMF for 10 min. Then, a solution of 10 eq. of $NaCNBH_3$ in dry DMF was added and the solution was shaken for 24 hours at room temperature. This procedure was repeated once, after which the resin was washed and the peptide was cleaved from the solid support and purified by HPLC using a gradient of B from 25 to 60 % in 45 min (t_R: 27.8 min).

Synthesis of 16

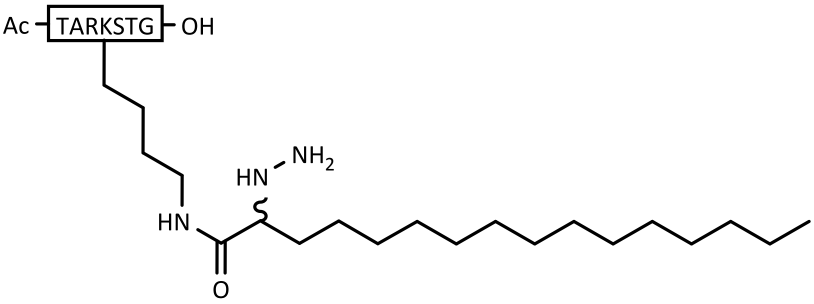

The precursor was shaken for two hours together with a solution containing 5 % (*v/v*) hydrazine monohydrate in *N*-methyl-2-pyrrolidone (NMP) (3 : 5 *v/v*) at 70°C. This procedure was repeated twice, after which the resin was washed and the peptide was cleaved from the solid support and purified by HPLC using a gradient of B from 25 to 60 % in 45 min (t_R: 18.2 min).

Synthesis of 17

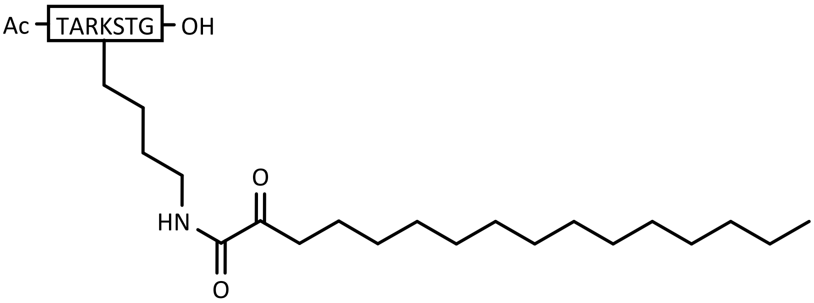

10 was cleaved as usual and the crude product was then redissolved in a solution containing 8 mM $CuSO_4$, 0.5 M glyoxalic acid and 10 % (*v/v*) pyridine in H_2O [8]. After shaking the solution for 60 min at room temperature, the reaction mixture was acidified using TFA and the product was isolated by HPLC using a gradient of B from 25 to 60 % in 45 min (t_R: 19.1 min).

Synthesis of 18

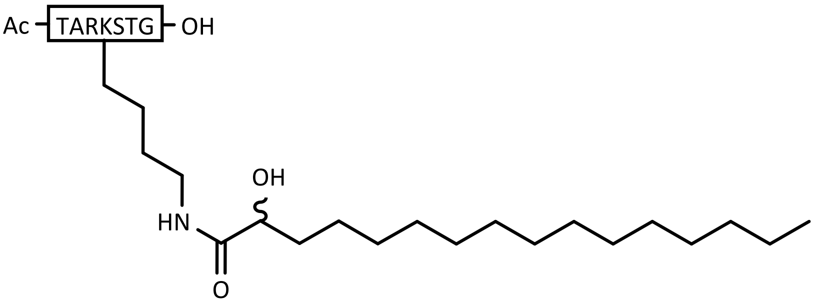

1 eq. of crude **17** was shaken together with 5 eq. $NaBH_4$ in water. After 20 min at room temperature a small volume of TFA was added and the product was isolated by HPLC using a gradient of B from 25 to 60 % in 45 min (t_R: 19.4 min).

Synthesis of 19 – 22

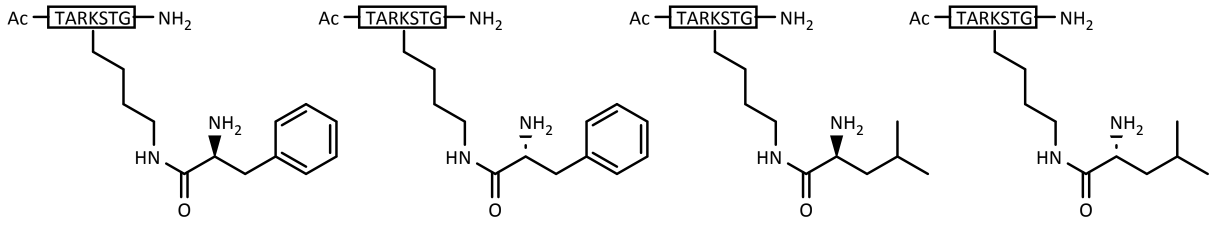

Following the nosyl cleavage, a solution of 8 eq. of DIPEA, 4 eq. of 2-(1H-benzotriazol-1-yl)-1,1,3,3-tetramethyluronium hexafluorophosphate (HBTU) and 4 eq. of the respective Boc-protected amino acid in dry DMF were added to the resin. After shaking for 45 min at room temperature, the resin was washed and the procedure repeated once. Subsequent to this, the resin was washed once more and the peptide was cleaved from the solid support and purified by HPLC using a gradient of B from 5 to 50 % in 45 min (t_R of **19** and **20**: 15.2 min, t_R of **21** and **22**: 12.4 min).

Synthesis of *(S)*-2-(Boc-amino)-4-thiapalmitic acid

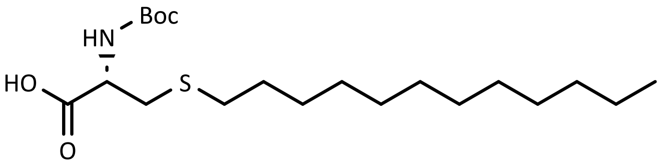

In an N_2-atmosphere, 1 eq. of D-cysteine was dissolved in dry MeOH and, while stirring, a solution containing 3.2 eq. of 1,1,3,3-tetramethylguanidine in dry MeOH was added dropwise. After 5 min, 1 eq. of 1-iodododecane in dry THF was added dropwise. The reaction was then heated up to 50 °C and stirred for three hours. Subsequently, the solution was allowed to cool to room temperature and stirred overnight. Subsequently, the solvents were removed *in vacuo* and the residue was dissolved in ethyl acetate and washed with H_2O. After drying over Na_2SO_4, the organic solvent was evaporated *in vacuo*, and a solution containing 1.3 eq. of di-tert-butyl dicarbonate (Boc_2O) and 3 eq. of DIPEA in dry DMF : DCM (2 : 1 *v/v*) was added to the crude intermediate and stirred for three hours at 40 °C. The solvents were evaporated, followed by a water/ethyl acetate extraction to obtain 2-(Boc-amino)-4-thia palmitic acid in satisfactory purity. It was used without purification for forthcoming reactions.

Synthesis of 23 and 24

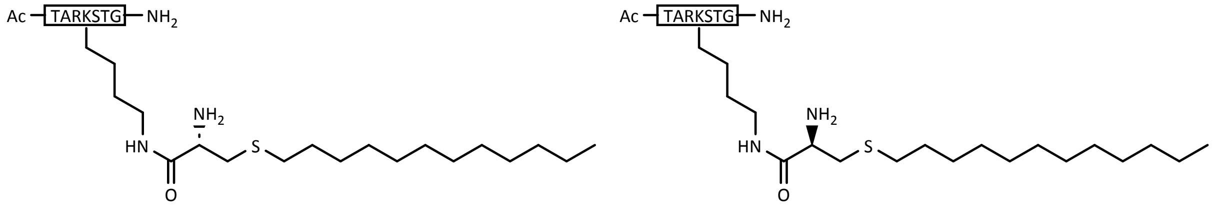

Following the nosyl cleavage, a solution of 4 eq. of 1-ethyl-3-(3-dimethylaminopropyl)carbodiimide (EDC), 2.5 eq. of Oxyma and 2 eq. of *(S)*- or *(R)*-2-(Boc-amino)-4-thiapalmitic acid in dry DMF : DCM (1 : 2 *v/v*) is prepared and added to the peptide on the resin. After shaking for 60 min at room temperature, the resin was washed and the procedure repeated once. Subsequent to this, the resin was washed once more and the peptide was cleaved using a solution containing TIPS (2.5% *v/v*), 1-

dodecanethiol (5% *v/v*), water (2.5% *v/v*) and TFA (90 % *v/v*). Following this, the peptide was purified by HPLC using a gradient of B from 25 to 60 % in 45 min (t_R: 20.2 min).

Synthesis of 25 – 28

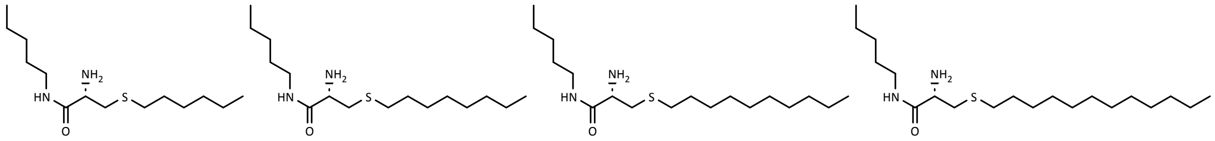

Firstly, the corresponding *(S)*-2-(Boc-amino)-4-thia building block was synthesised utilising 1-iodohexane, 1-iodooctane or 1-bromodecane as described above. Afterwards, 1 eq. of pentylamine was stirred with 2 eq. of the corresponding 2-(Boc-amino)-4-thia building block, 2 eq. of EDC and 4 eq. of Oxyma in dry DMF for two hours at room temperature. Subsequently, a number of volumes of water were added, and the product was extracted using ethyl acetate. Following the removal of the organic solvent *in vacuo*, the crude product was purified by HPLC using a gradient of B from 45 to 95 % in 60 min (t_R **25**: 28.4 min, t_R **26**: 30.8 min, t_R **27**: 33.2 min, t_R **28**: 36.5 min). The product-containing fractions were combined and lyophilised. Following lyophilisation, the product was treated with pure TFA for one hour. The TFA was then evaporated, after which the product was lyophilised once more.

Synthesis of 29

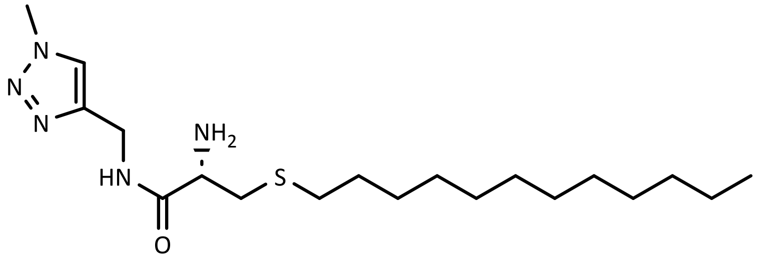

2 eq. of propargylamine were stirred with 1 eq. of *(S)*-2-(Boc-amino)-4-thiapalmitic acid, 1 eq. of EDC and 2 eq. of Oxyma in dry DMF for two hours at room temperature. Subsequently, a number of volumes of water were added, and the product was extracted using ethyl acetate. Following the removal of the organic solvent *in vacuo*, a solution containing 0.4 eq. of $CuSO_4$, 0.8 eq. of ascorbic acid, 3 eq. of Na_2CO_3, in water and DMF (1 : 4 *v/v*) was added. After 5 min of preincubation, a solution containing 1.1 eq. of methyliodide and 1.2 eq. of sodium azide in water and DMF (1 : 4 *v/v*) was added. The reaction was stirred over night at room temperature under a N_2-atmosphere. After the addition of several volumes of water, the product was extracted with ethyl acetate. The organic solvent was then removed under vacuum, and the crude product was purified by HPLC using a gradient of B from 35 to 85 % in 60 min (t_R: 21.8 min). The product-containing fractions were combined and lyophilised. Following lyophilisation, the product was treated with pure TFA for one hour. The TFA was then evaporated, after which the product was lyophilised once more.

Synthesis of 30

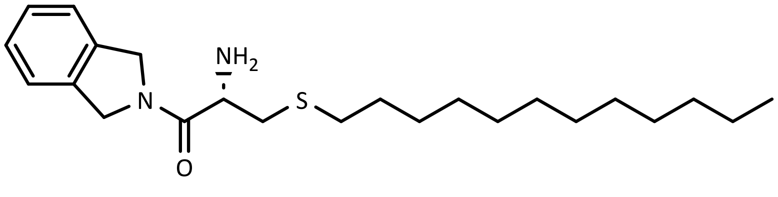

2 eq. of isoindoline was stirred with 1 eq. of 2*(S)*-2-(Boc-amino)-4-thiapalmitic acid, 2 eq. of HATU and 5 eq. of DIPEA in dry DMF for two hours at room temperature. Subsequently, a number of volumes of water were added, and the product was extracted using ethyl acetate. Following the removal of the organic solvent *in vacuo*, the crude product was purified by HPLC using a gradient of B from 45 to 95 % in 60 min (t_R: 25.1 min). The product-containing fractions were combined and lyophilised. Following lyophilisation, the product was treated with pure TFA for one hour. The TFA was then evaporated, after which the product was lyophilised once more.

Synthesis of 31

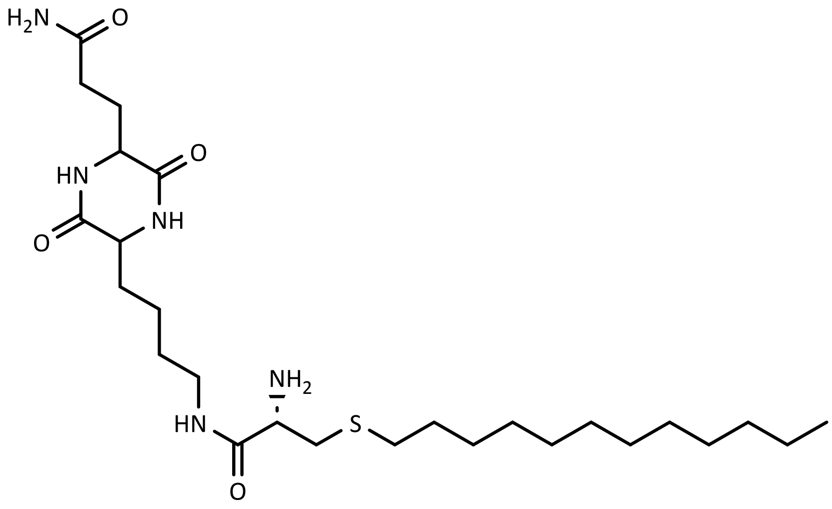

In the first step, H-Gln-OMe was prepared by dissolving glutamine in dry MeOH under a N_2-atmosphere. The reaction was then cooled to 0 °C with stirring, after which 5 eq. of $SOCl_2$ was added dropwise and the reaction was stirred for 2 h at 0 °C, after which the ice bath was removed and the reaction was stirred overnight at room temperature with a $CaCl_2$ tube attached. The next day, MeOH was removed *in vacuo*. To the crude product 1.5 eq. of Fmoc-Lys(Fmoc)-OH, 1.5 eq. of EDC and 3 eq. of Oxyma dissolved in dry DMF were added and stirred for 1 h at room temperature. After the addition of several volumes of water, the product was extracted with DCM. The organic layer was then washed and dried before removal of the organic solvent *in vacuo*. Cyclization was carried out simultaneously to the Fmoc-removal by adding a piperidine solution in DMF (20 % *v/v*) and stirring for 60 min at 40°C. Again, several volumes of water were added and the product was purified by extraction of impurities with petroleum ether. The crude cyclised dipeptide was then attached to FMP resin by reductive amination using 2 eq. of peptide, 2 eq. of $NaBH(OAc)_3$ and 3 eq. of collidine in dry DMF. The reaction was shaken for 24 hours at room temperature. The resin was then washed and 3 eq. of *(S)*-2-(Boc-amino)-4-thiapalmitic acid, 3 eq. of EDC and 6 eq. of Oxyma were added in dry DMF and the reaction was shaken overnight at room temperature. The crude product was then cleaved using a solution containing TIPS (2.5% *v/v*), 1-dodecanethiol (5% *v/v*), water (2.5% *v/v*) and TFA (90 % *v/v*) for two times 2 hours. Following this, the compound was purified by HPLC using a gradient of B from 40 to 90 % in 60 min (t_R: 32.0 min).

Synthesis of 32

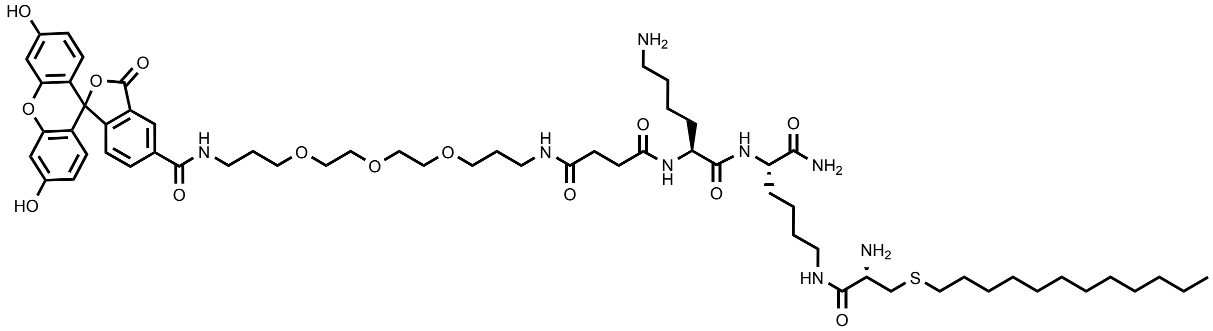

The dipeptide was prepared on Rink amid resin as previously described. However, in place of the final acetylation, a Fmoc-TTDS linker was introduced. Therefore, the nosyl group was removed and 3 eq. of *(S)*-2-(Boc-amino)-4-thiapalmitic acid were coupled using 3 eq. of EDC and 6 eq. of Oxyma in dry DMF. Afterwards, the resin was washed and the Fmoc group was removed using piperidine in DMF (20 % *v/v*). The next step was coupling 2 eq. 5-carboxyfluoresceine diacetate to the linker using together with 2 eq. of HATU and 5 eq. of DIPEA. After complete coupling, the acetyl groups were removed using 5 eq. of KOH in DMF. Subsequent to this, the resin was washed once more and the peptide was cleaved from the solid support and purified by HPLC using a gradient of B from 30 to 85 % in 60 min (t_R: 34.9 min).

D. Dose-response curves

1

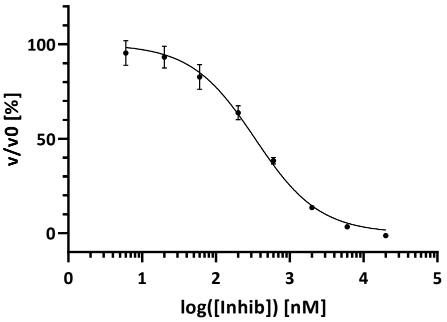

2

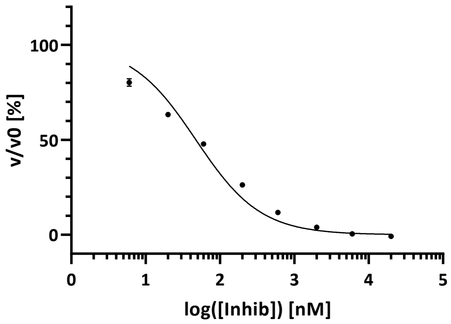

8

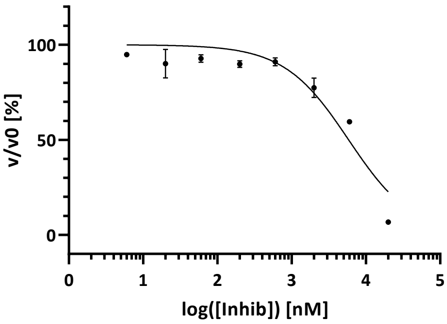

9

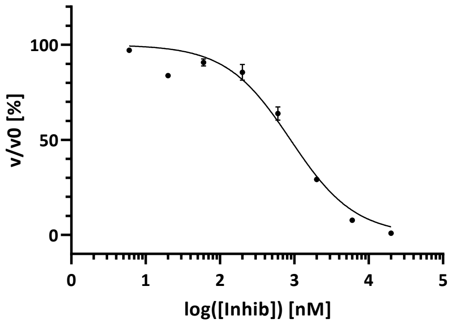

10

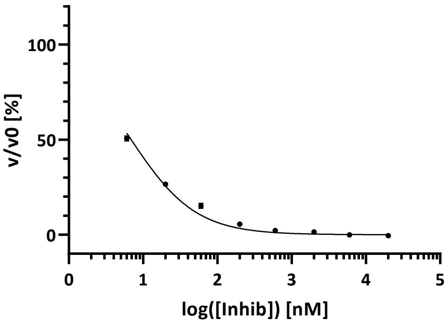

13

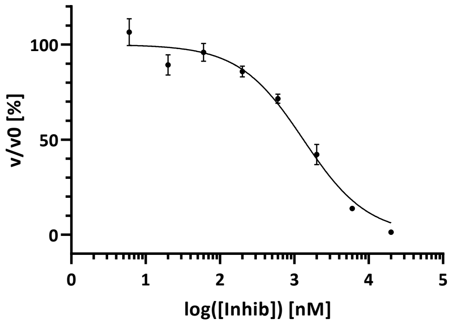

14

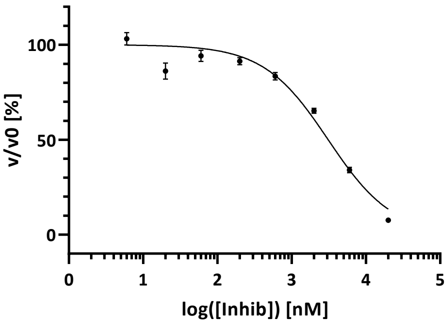

15

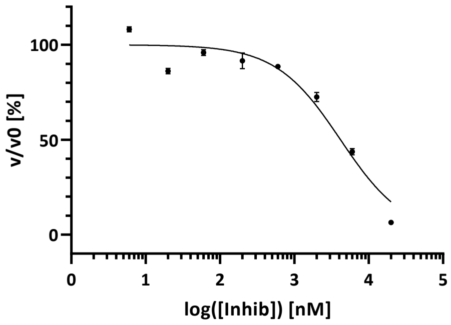

16

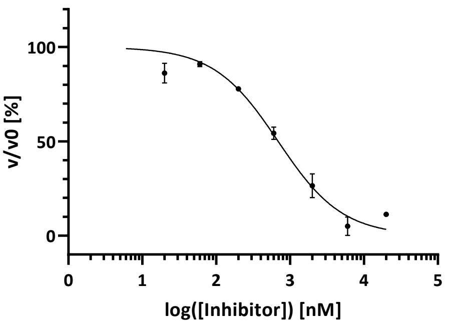

17

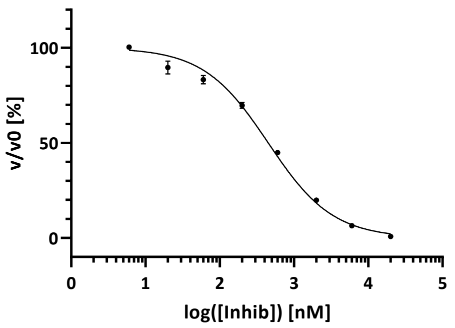

18

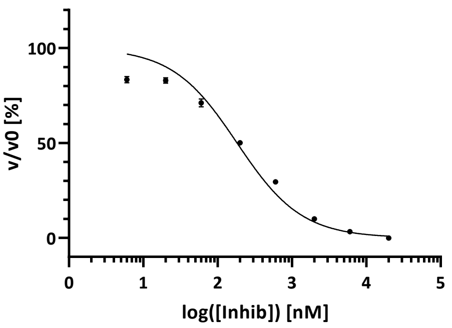

23

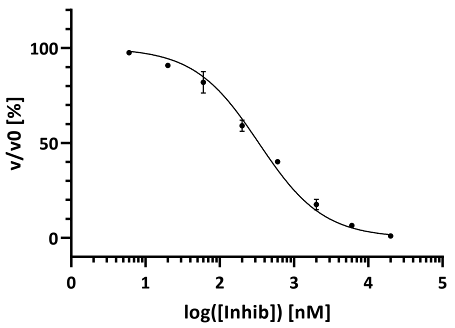

24

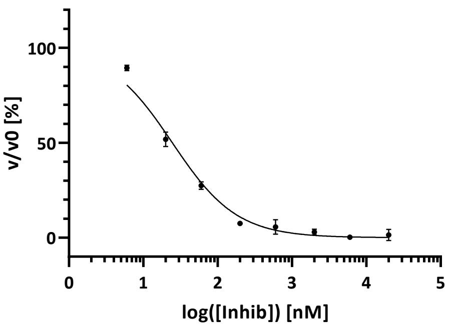

25

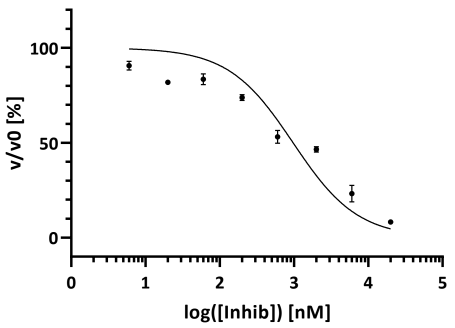

26

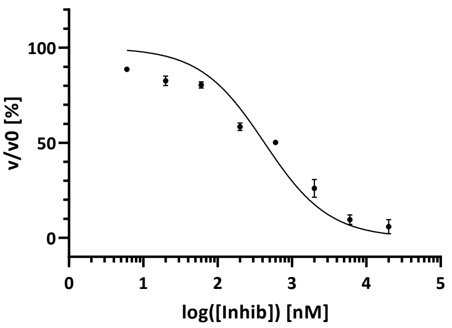

27

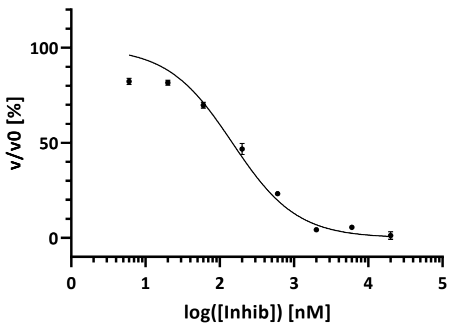

E. UPLC-MS data

Table S1. Calculated and Observed Masses of Synthesized Compounds

compound	Calculated mass (g/mol)	Found m/z of $[M+H]^+$	Found m/z of $[M+2H]^{2+}$
1	1073.62	1074.03	537.67
2	1031.60	1032.02	516.67
3	1056.60	1057.03	529.17
4	1091.61	1091.74	
5	1139.61	1139.24	
6	1139.61	1139.76	
7	1115.62	1116.63	558.86
8	1093.58	1093.94	547.68
9	1040.63	1041.02	521.17
10	1014.64	1015.62	508.36
11	1028.66	1029.12	515.27
12	1042.68	1043.12	522.27
13	1056.69	1057.13	529.27
14	1065.65	1066.23	533.77
15	1104.69	1105.04	553.28
16	1029.65	1030.12	515.77
17	1013.25	1013.62	507.46
18	1015.63	1016.01	508.67
19	907.49	908.61	455.26
20	908.03	908.51	454.86
21	873.5	874.70	437.86
22	874.01	874.60	437.86
23	1031.62	1032.62	516.86
24	1032.31	1032.62	516.86
25	274.21	275.26	
26	302.24	303.36	
27	330.27	331.36	
28	358.30	359.47	
29	383.27	384.36	
30	390.27	391.36	
31	527.77	528.36	
32	1205.52	1205.65	603.47

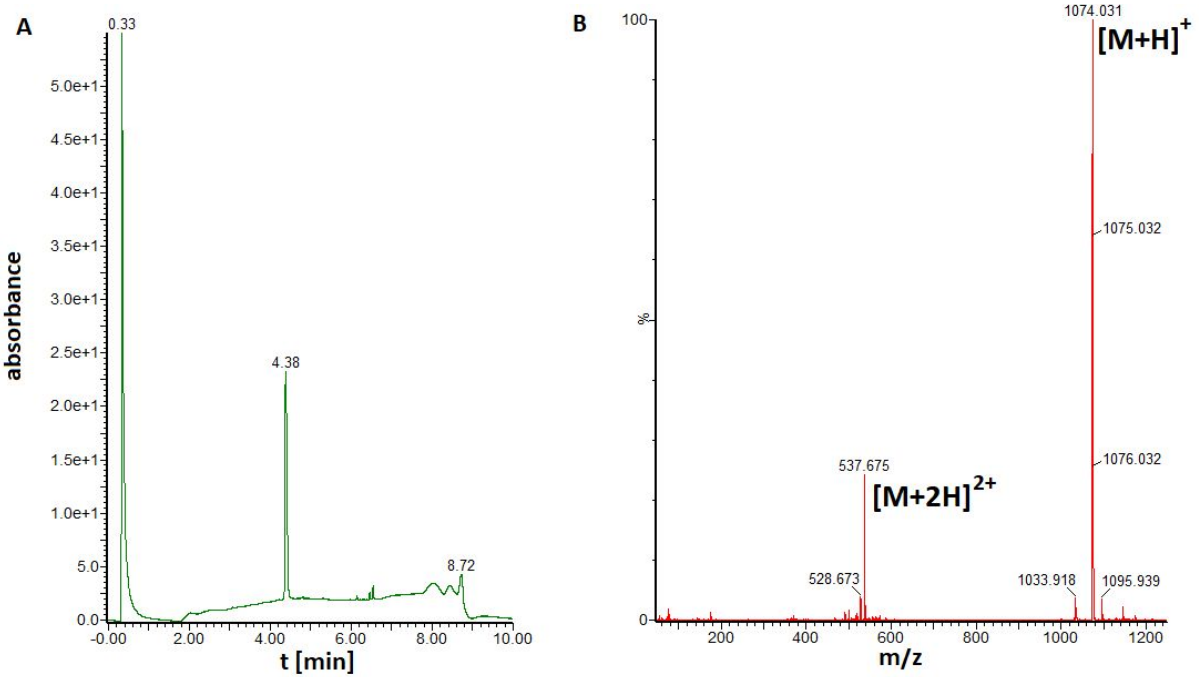

Figure S 2: UPLC-MS Spectra of compound ***1***. ***A***. *The photometric analysis was done between 200 to 400 nm.* ***B***. *Mass spectra of the peak at 4.38 min*

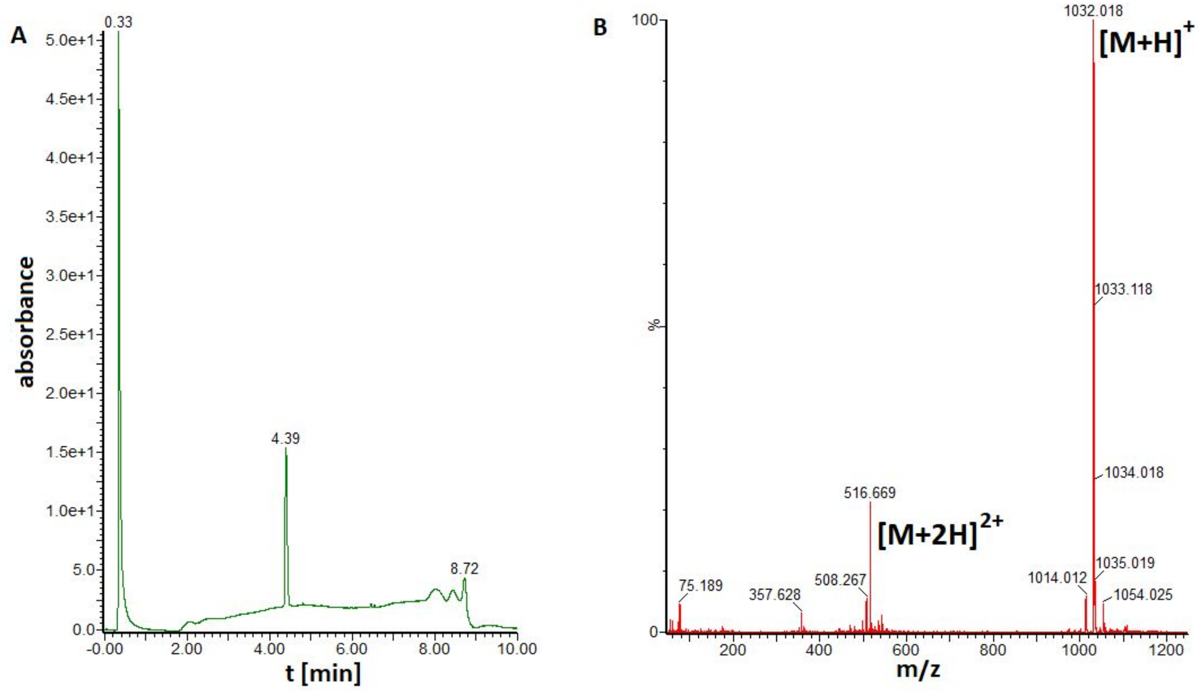

Figure S 3: UPLC-MS Spectra of compound ***2***. ***A***. *The photometric analysis was done between 200 to 400 nm.* ***B***. *Mass spectra of the peak at 4.39 min*

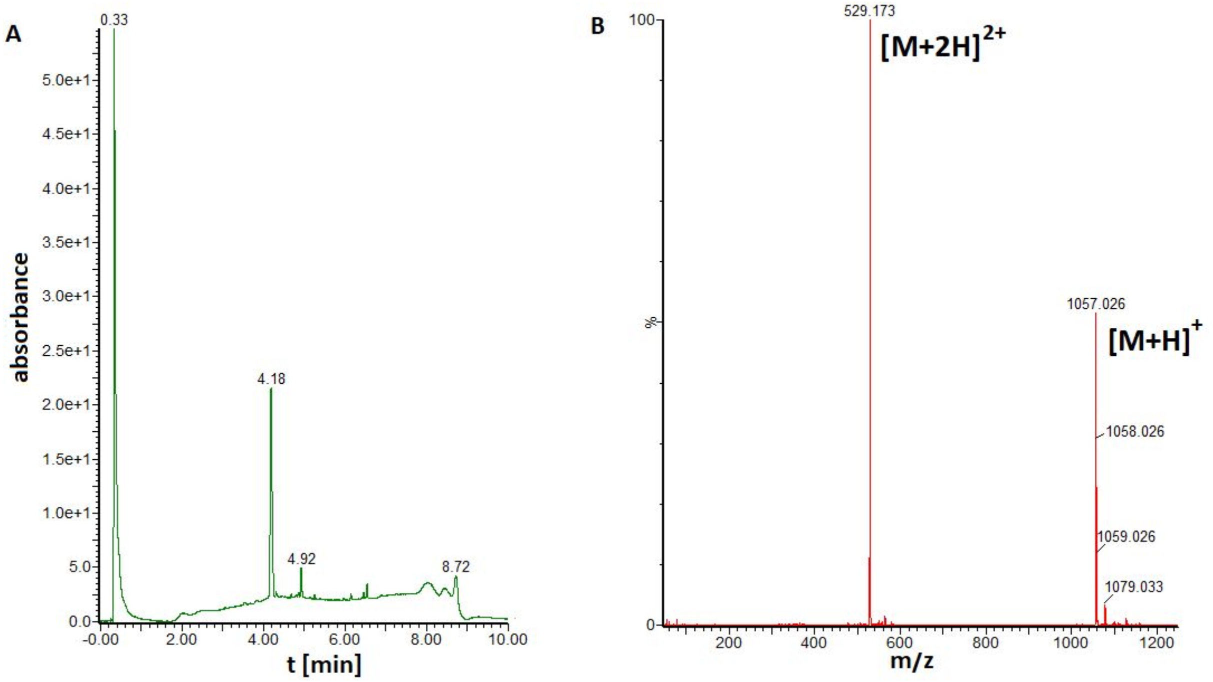

*Figure S 4: UPLC-MS Spectra of compound **3**. **A**. The photometric analysis was done between 200 to 400 nm. **B**. Mass spectra of the peak at 4.18 min*

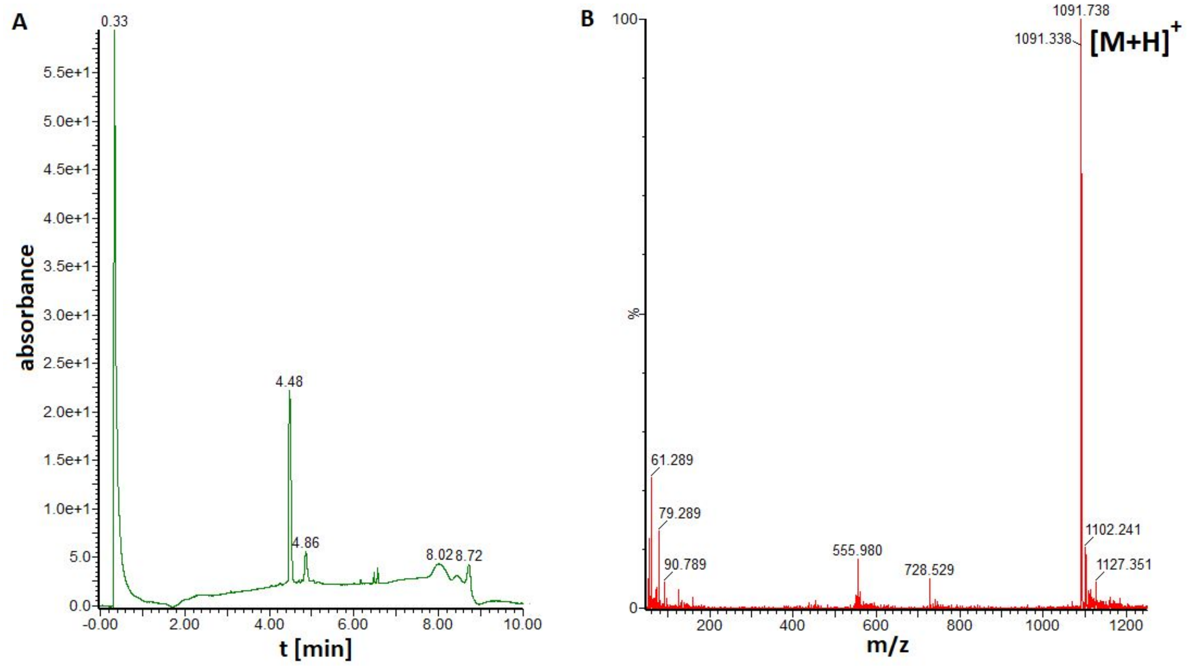

*Figure S 5: UPLC-MS Spectra of compound **4**. **A**. The photometric analysis was done between 200 to 400 nm. B. Mass spectra of the peak at 4.18 min*

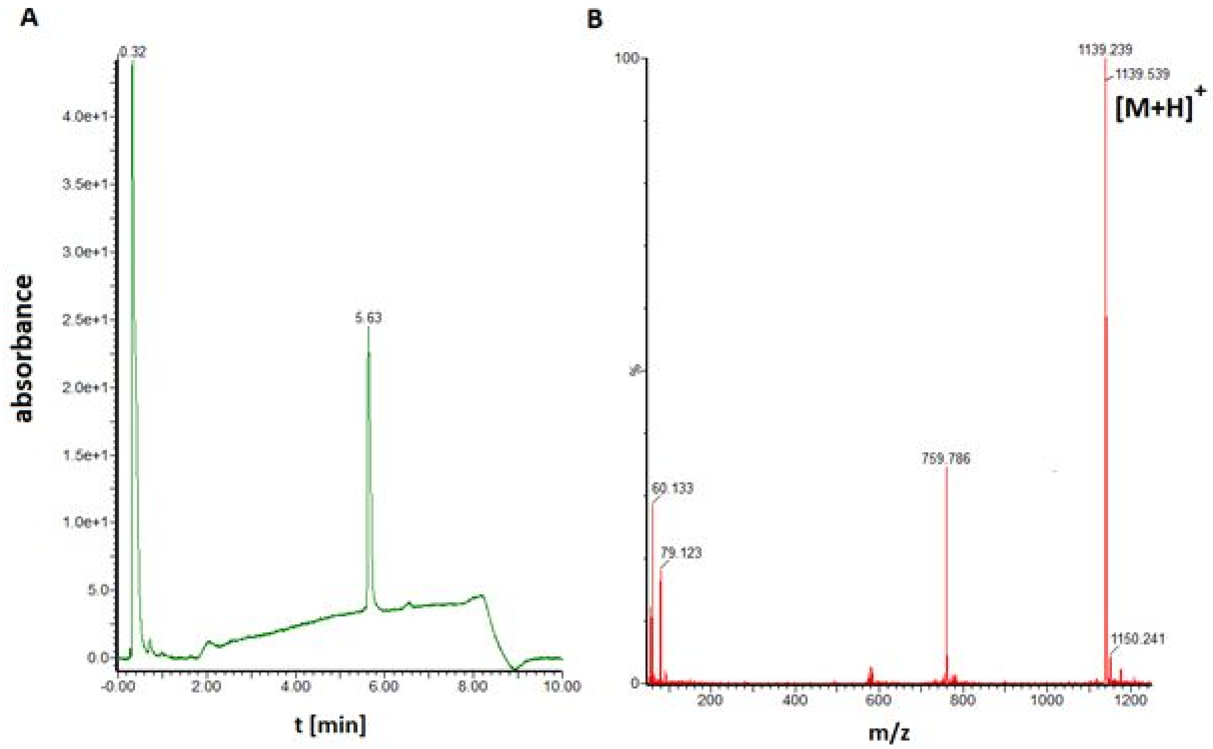

Figure S 6: UPLC-MS Spectra of compound ***5****.* ***A****. The photometric analysis was done between 200 to 400 nm.* ***B****. Mass spectra of the peak at 5.63 min.*

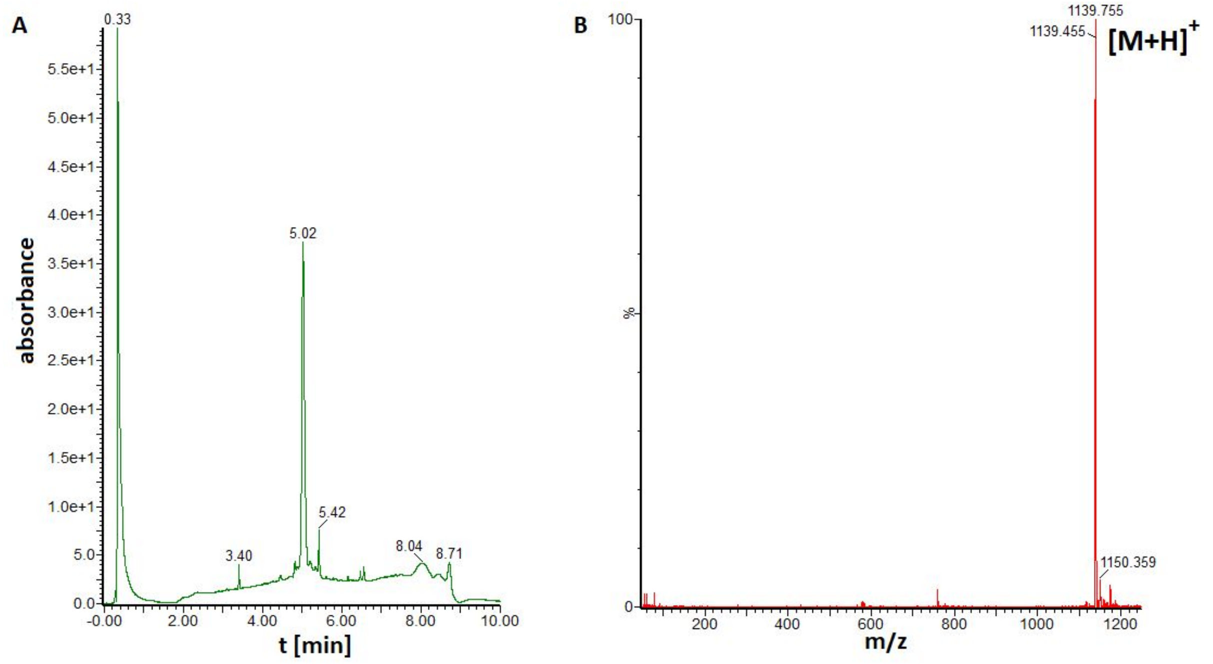

Figure S 7: UPLC-MS Spectra of compound ***6****.* ***A****. The photometric analysis was done between 200 to 400 nm.* ***B****. Mass spectra of the peak at 5.02 min*

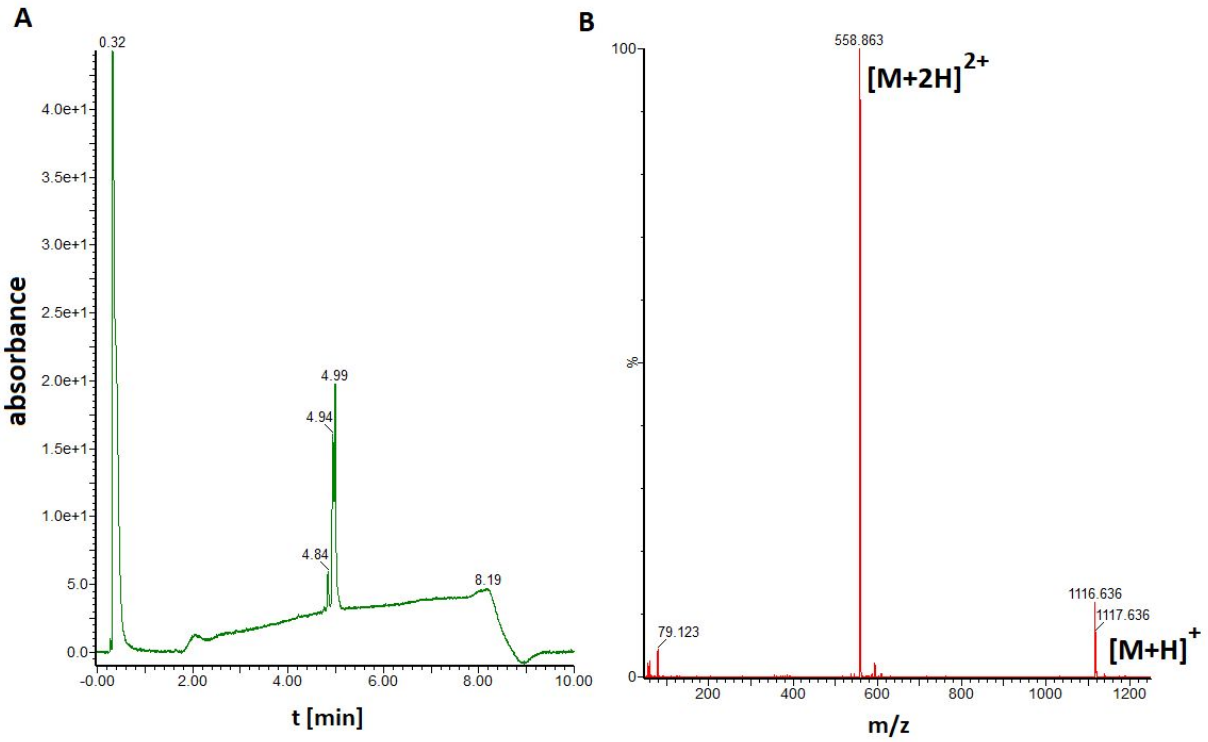

*Figure S 8: UPLC-MS Spectra of compound **7**. **A**. The photometric analysis was done between 200 to 400 nm. **B**. Mass spectra of the peak at 4.94 min.*

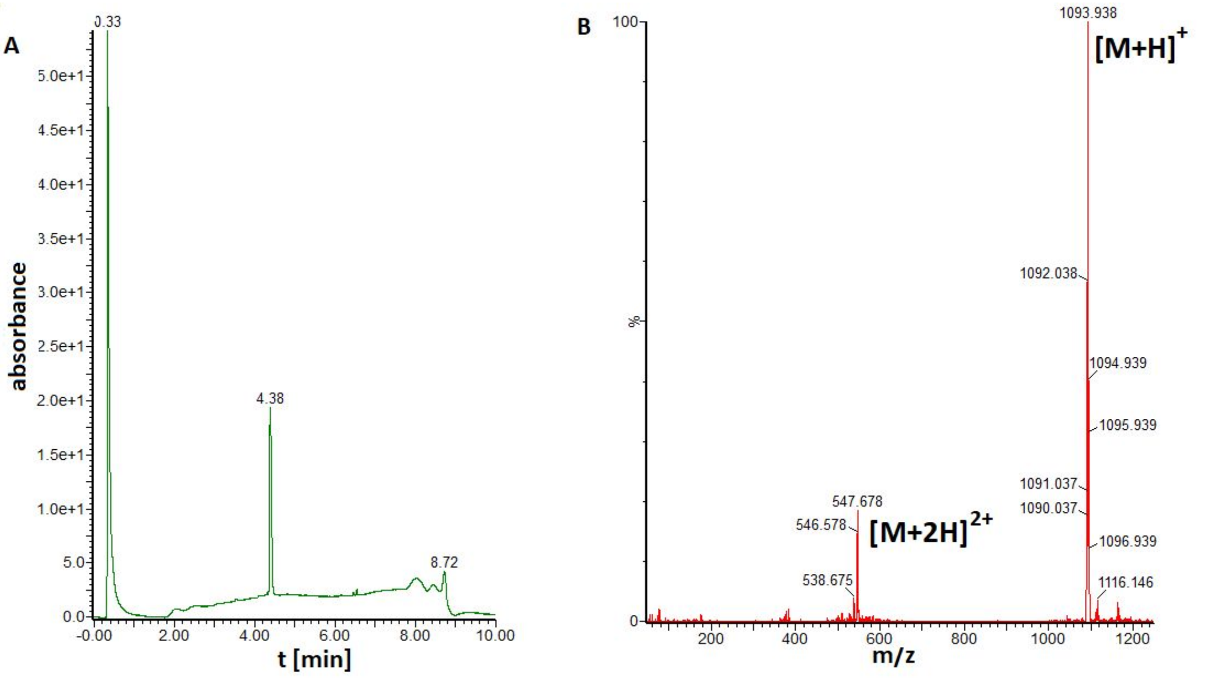

*Figure S 9: UPLC-MS Spectra of compound **8**. **A**. The photometric analysis was done between 200 to 400 nm. **B**. Mass spectra of the peak at 4.48 min.*

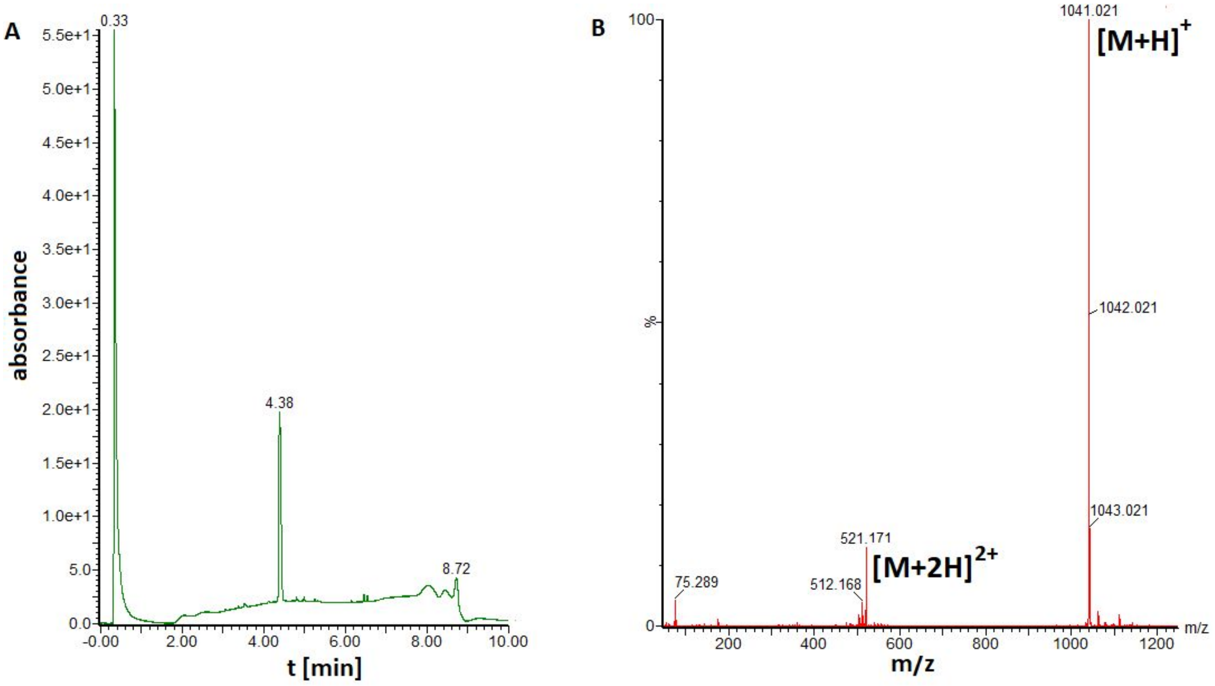

*Figure S 10: UPLC-MS Spectra of compound **9**. **A**. The photometric analysis was done between 200 to 400 nm. **B**. Mass spectra of the peak at 4.38 min.*

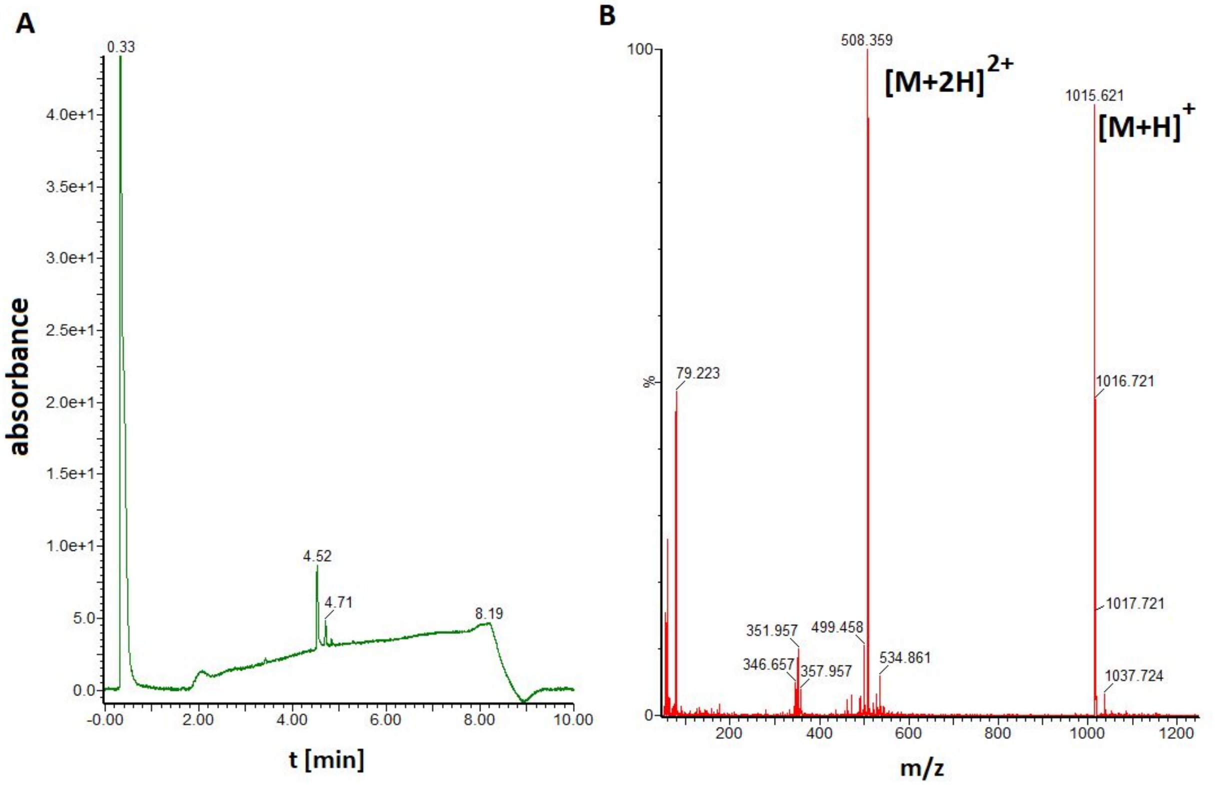

*Figure S 11: UPLC-MS Spectra of compound **10**. **A**. The photometric analysis was done between 200 to 400 nm. **B**. Mass spectra of the peak at 3.49 min.*

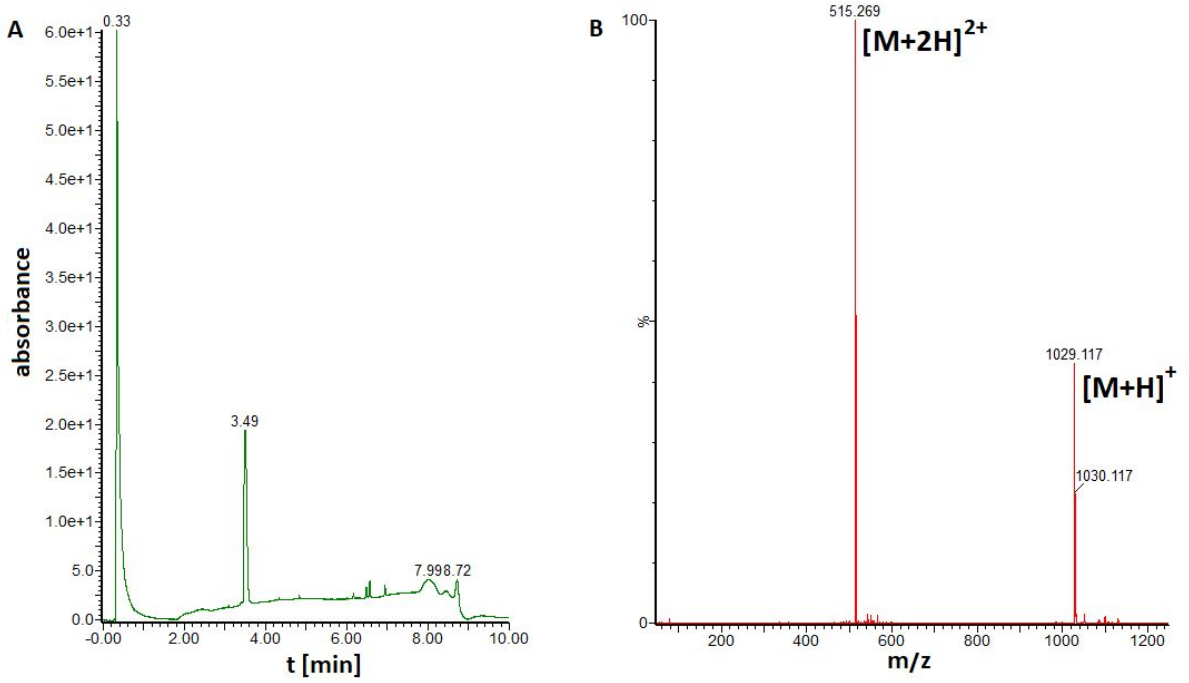

*Figure S 12: UPLC-MS Spectra of compound **11**. **A**. The photometric analysis was done between 200 to 400 nm. **B**. Mass spectra of the peak at 3.49 min.*

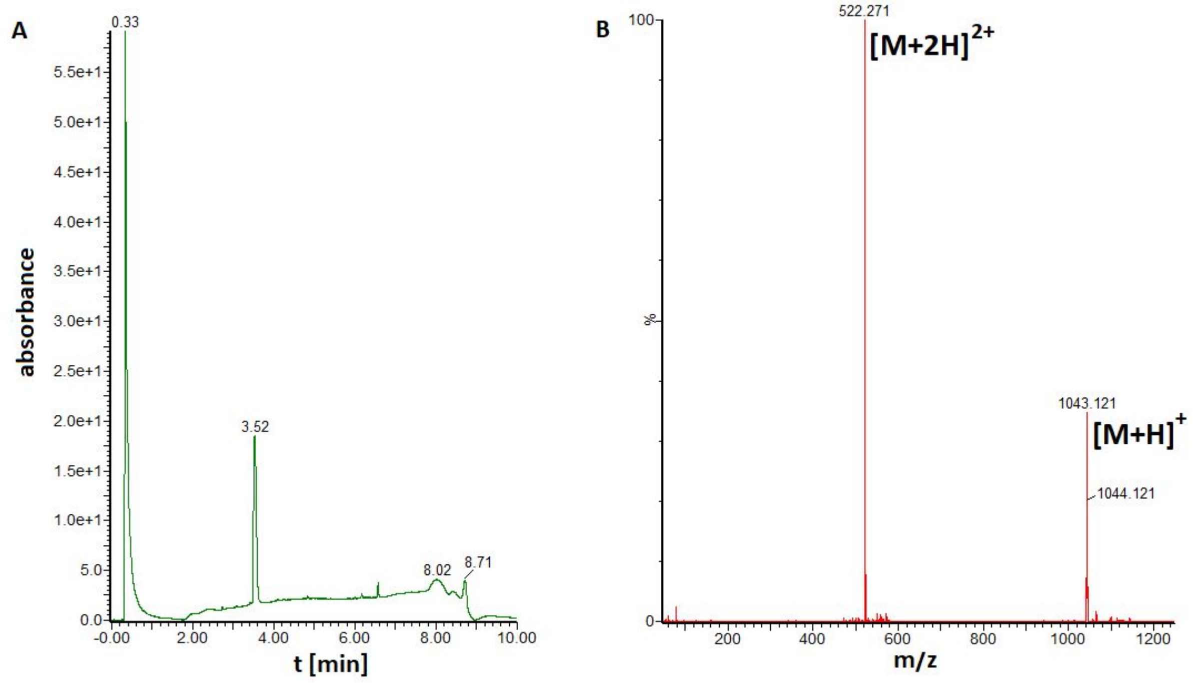

*Figure S 13: UPLC-MS Spectra of compound **12**. **A**. The photometric analysis was done between 200 to 400 nm. **B**. Mass spectra of the peak at 3.52 min.*

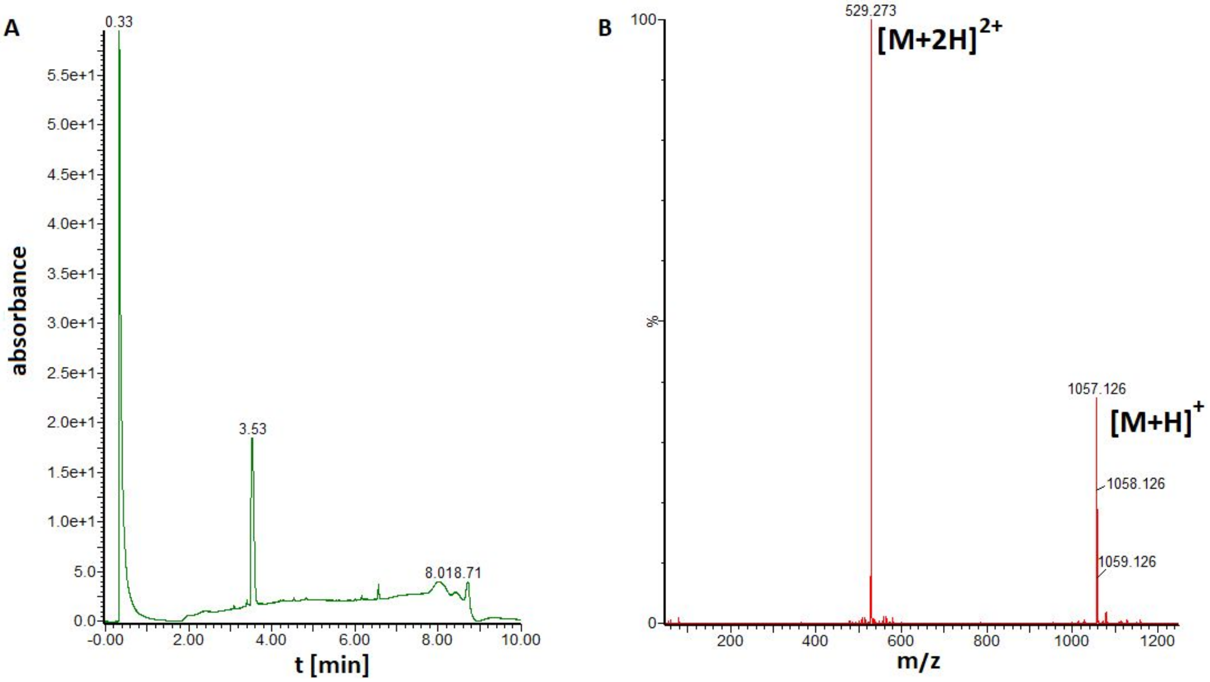

Figure S 14: UPLC-MS Spectra of compound ***13****.* ***A****. The photometric analysis was done between 200 to 400 nm.* ***B****. Mass spectra of the peak at 3.53 min.*

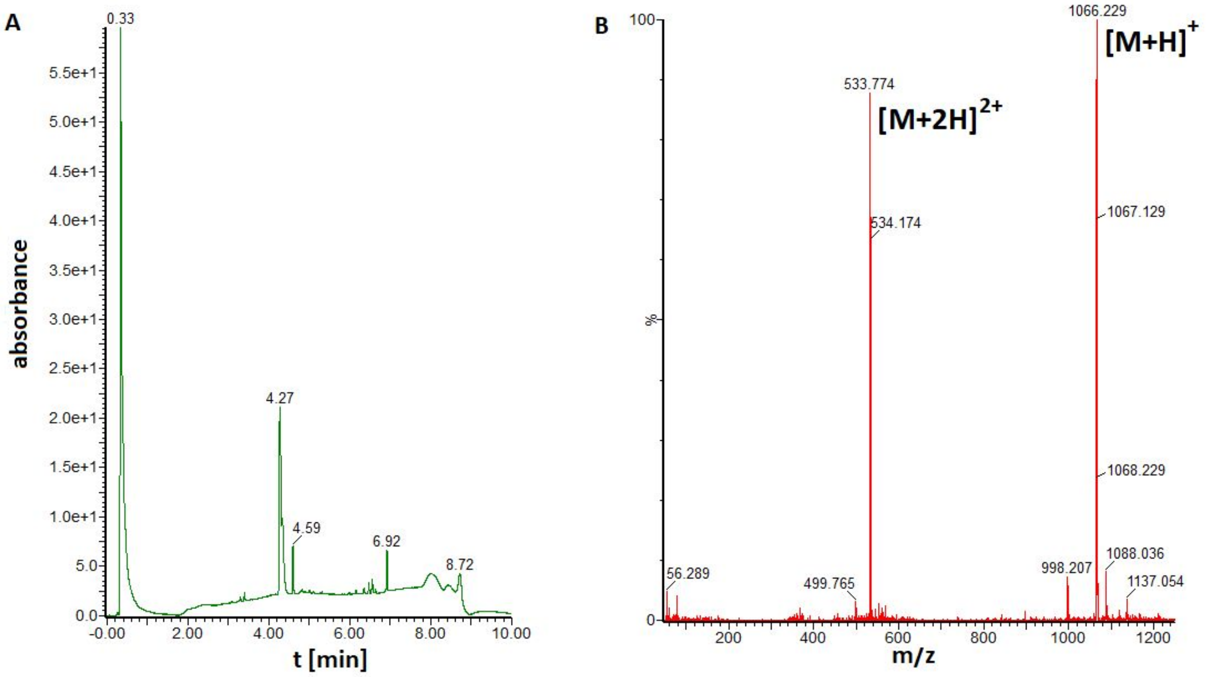

Figure S 15: UPLC-MS Spectra of compound ***14****.* ***A****. The photometric analysis was done between 200 to 400 nm.* ***B****. Mass spectra of the peak at 4.27 min.*

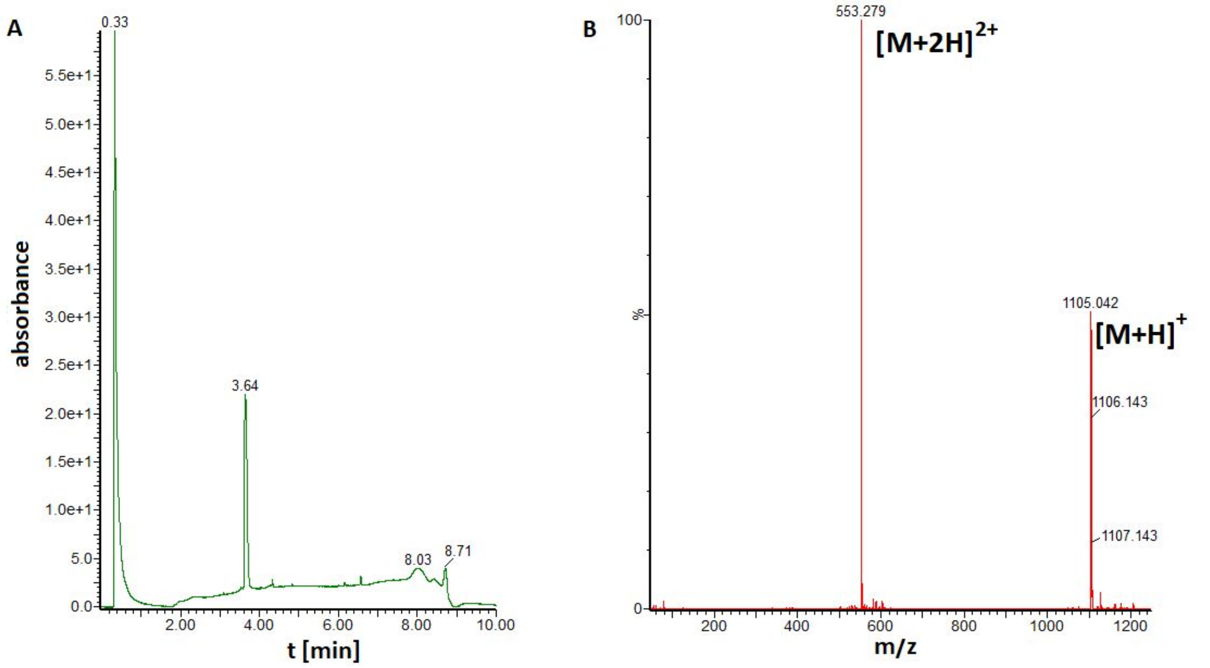

*Figure S 16: UPLC-MS Spectra of compound **15**. **A**. The photometric analysis was done between 200 to 400 nm. **B**. Mass spectra of the peak at 3.64 min.*

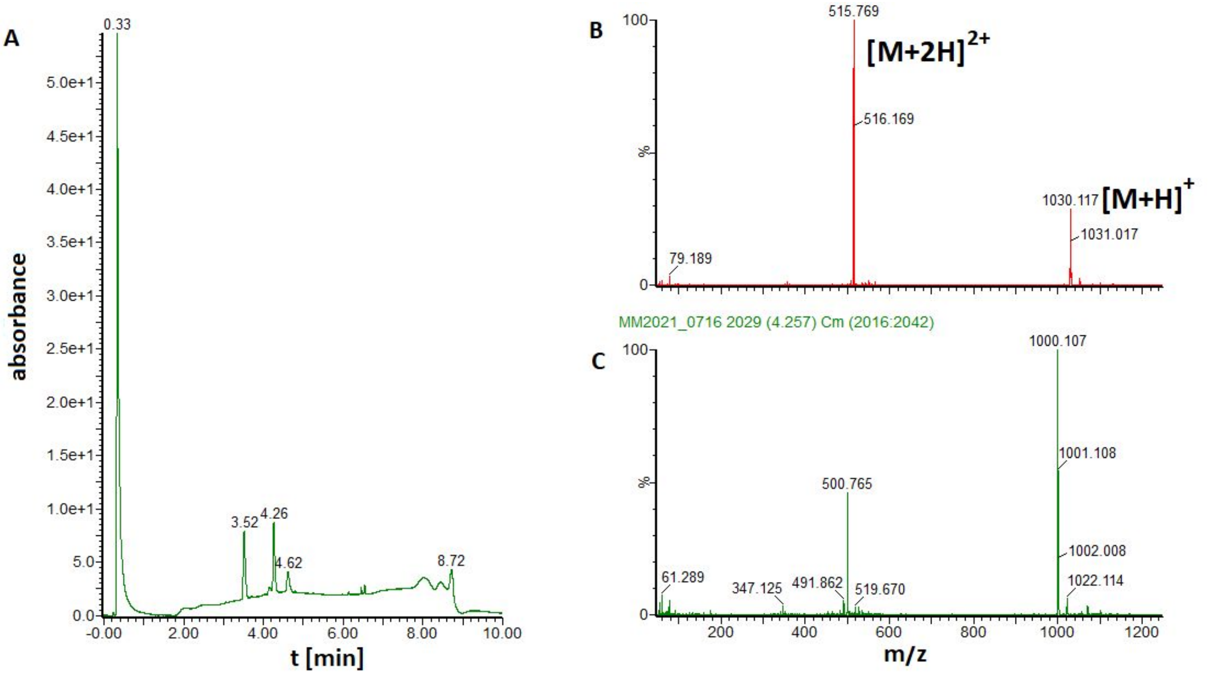

*Figure S 17: UPLC-MS Spectra of compound **16**. **A**. The photometric analysis was done between 200 to 400 nm. **B**. Mass spectra of the peak at 3.52 min. **C**. Mass spectra of the peak at 4.26 min.*

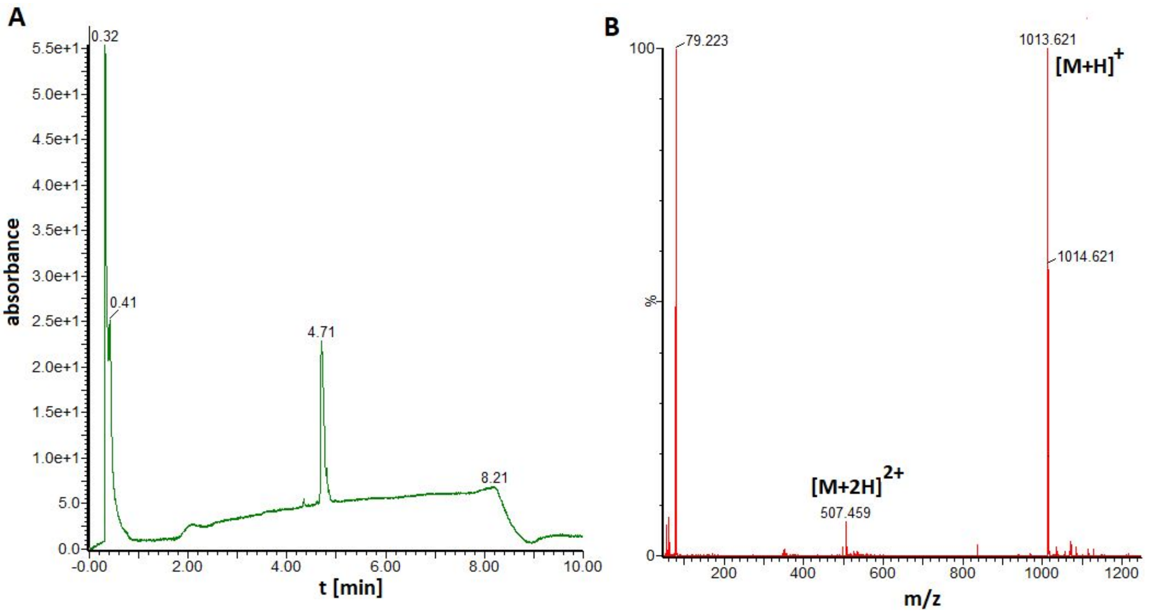

*Figure S 18: UPLC-MS Spectra of compound **17**. **A**. The photometric analysis was done between 200 to 400 nm. **B**. Mass spectra of the peak at 4.71 min.*

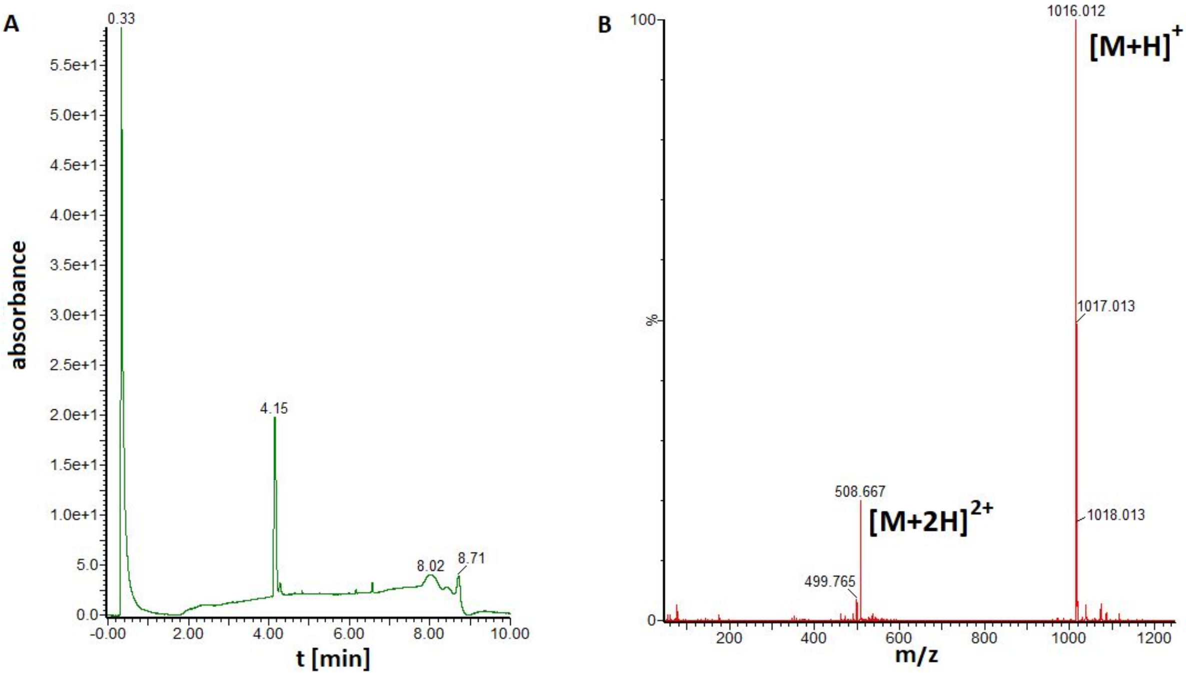

*Figure S 19: UPLC-MS Spectra of compound **18**. **A**. The photometric analysis was done between 200 to 400 nm. **B**. Mass spectra of the peak at 4.15 min.*

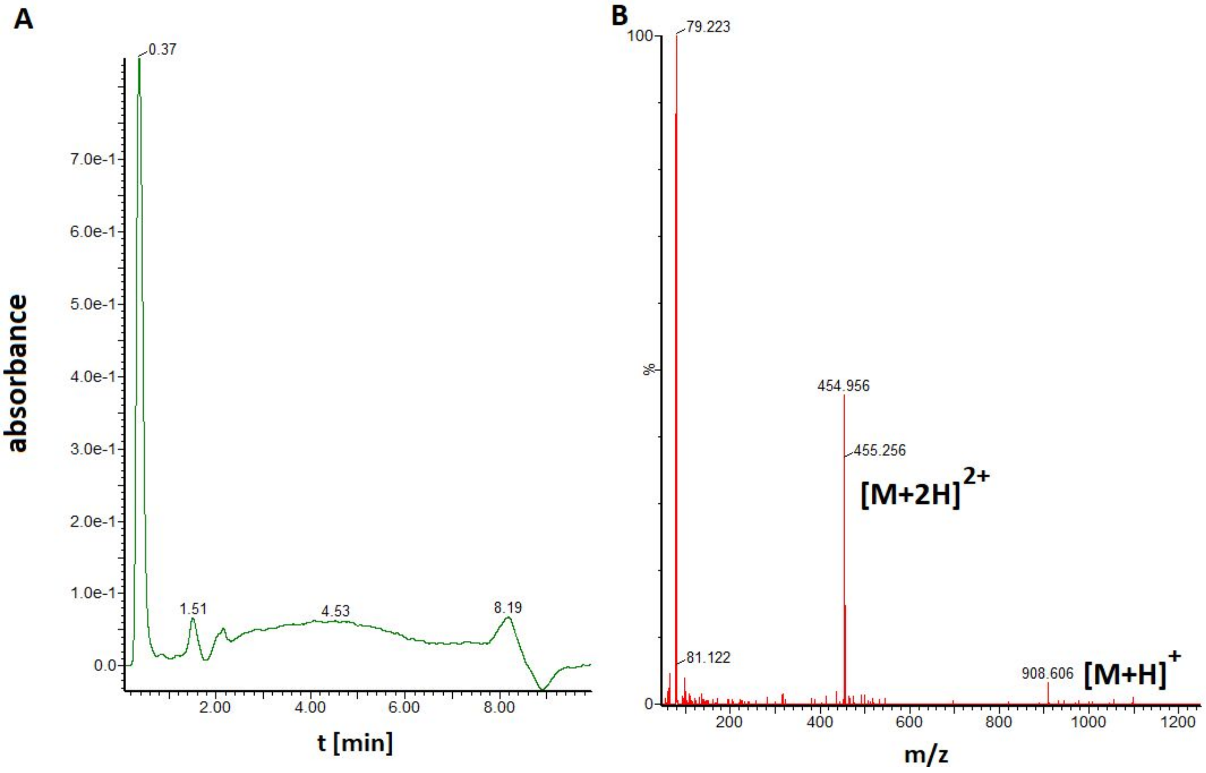

*Figure S 20: UPLC-MS Spectra of compound **19**. **A**. The photometric analysis was done between 200 to 400 nm. **B**. Mass spectra of the peak at 1.51 min.*

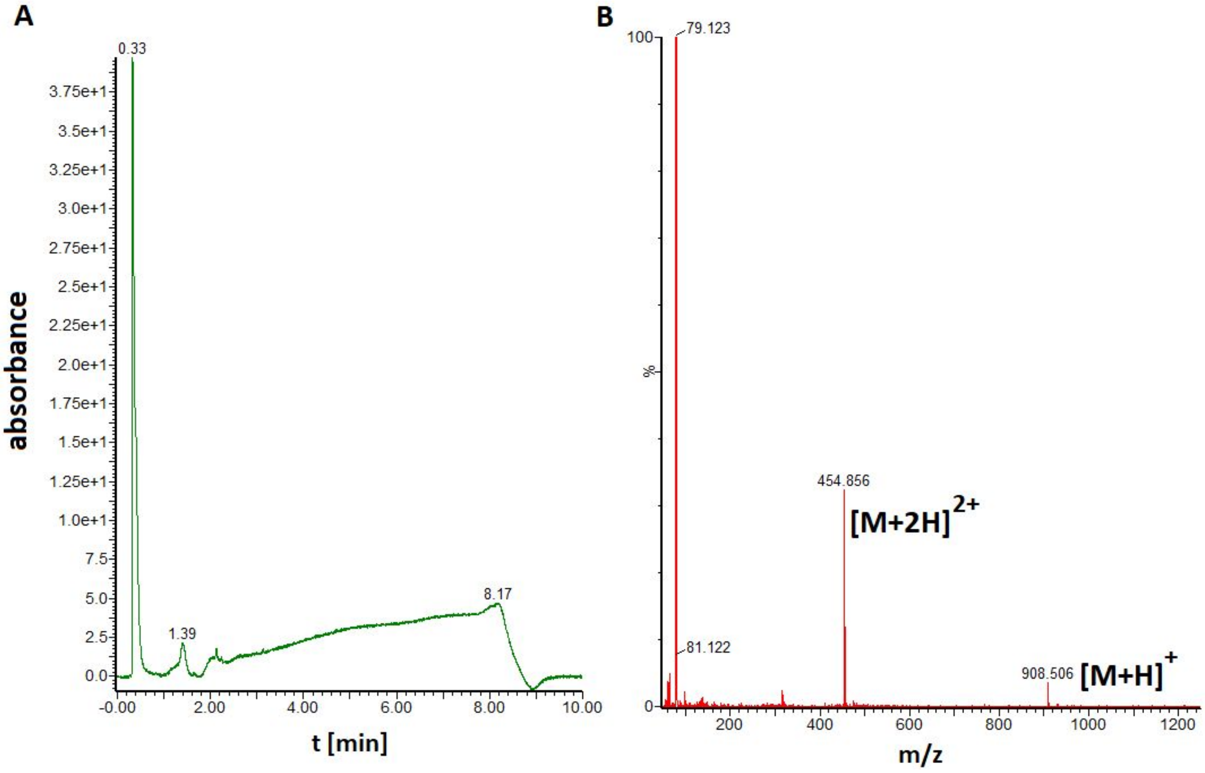

*Figure S 21: UPLC-MS Spectra of compound **20**. **A**. The photometric analysis was done between 200 to 400 nm. **B**. Mass spectra of the peak at 1.39 min.*

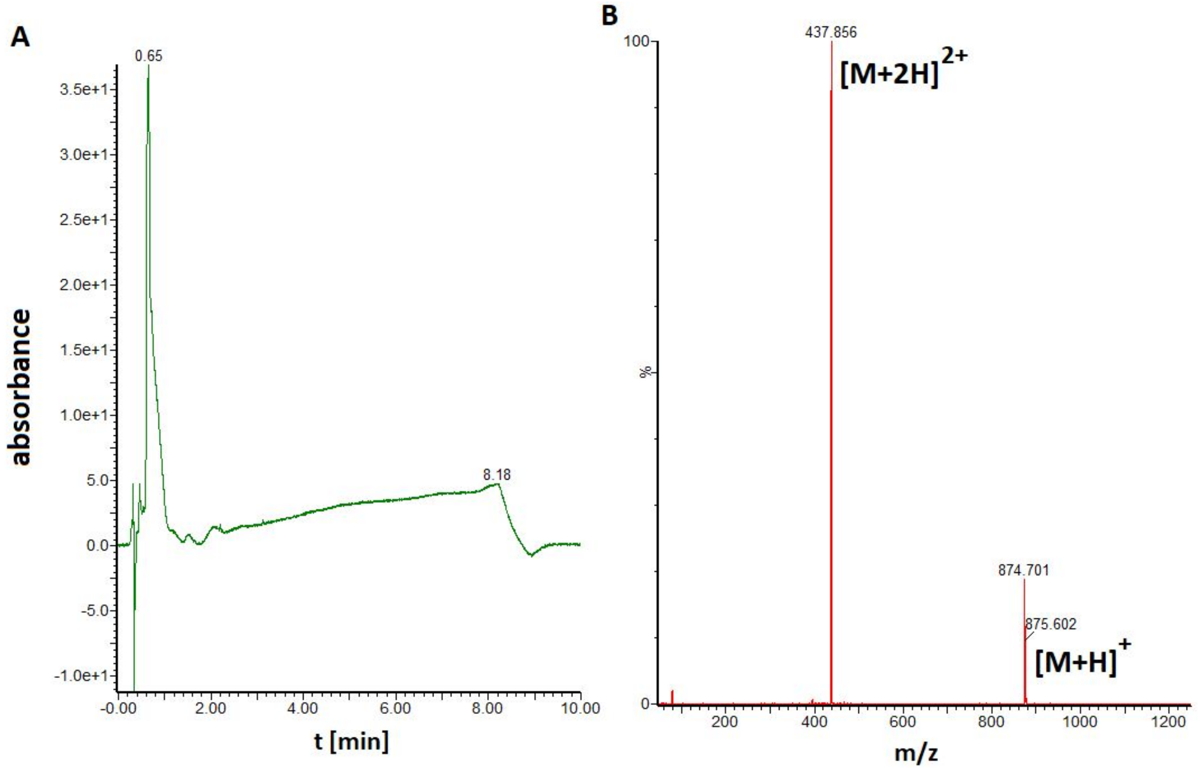

*Figure S 22: UPLC-MS Spectra of compound **21**. **A**. The photometric analysis was done between 200 to 400 nm. **B**. Mass spectra of the peak at 0.65 min.*

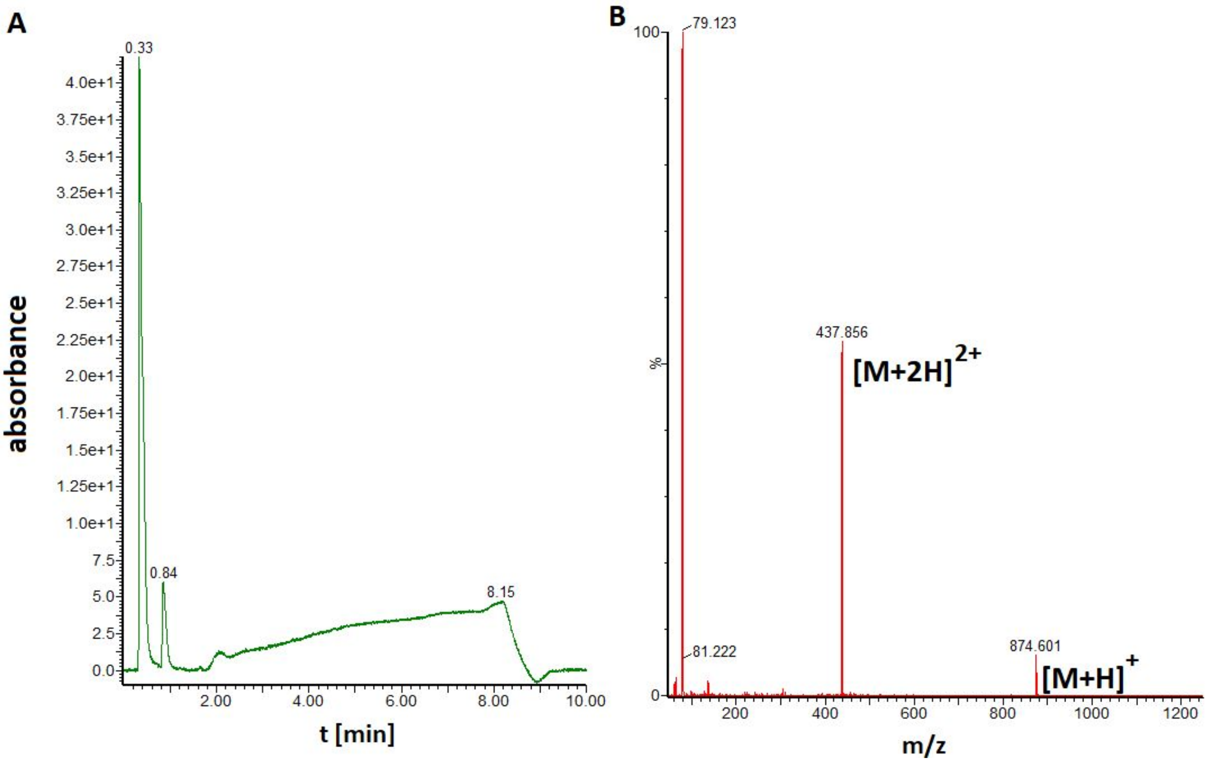

*Figure S 23: UPLC-MS Spectra of compound **22**. **A**. The photometric analysis was done between 200 to 400 nm. **B**. Mass spectra of the peak at 0.84 min.*

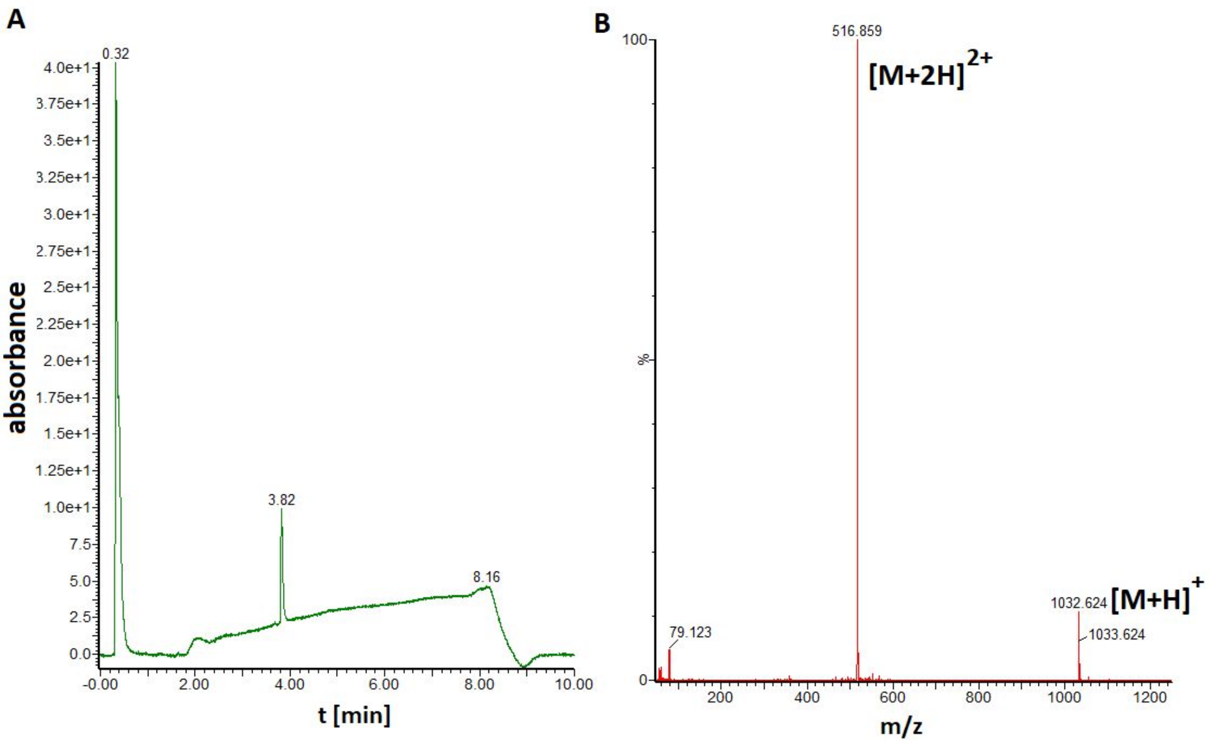

*Figure S 24: UPLC-MS Spectra of compound **23**. **A**. The photometric analysis was done between 200 to 400 nm. **B**. Mass spectra of the peak at 3.82 min.*

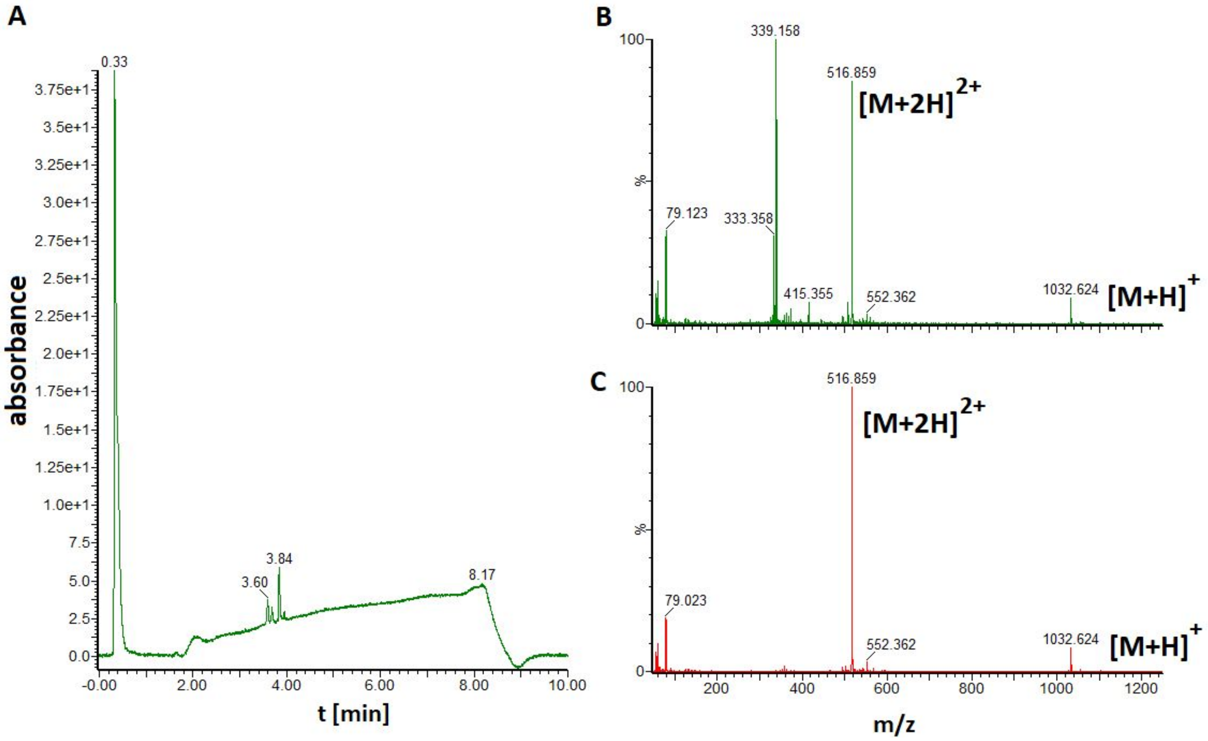

*Figure S 25: UPLC-MS Spectra of compound **24**. **A**. The photometric analysis was done between 200 to 400 nm. **B**. Mass spectra of the peak at 3.60 min. **C**. Mass spectra of the peak at 3.84 min.*

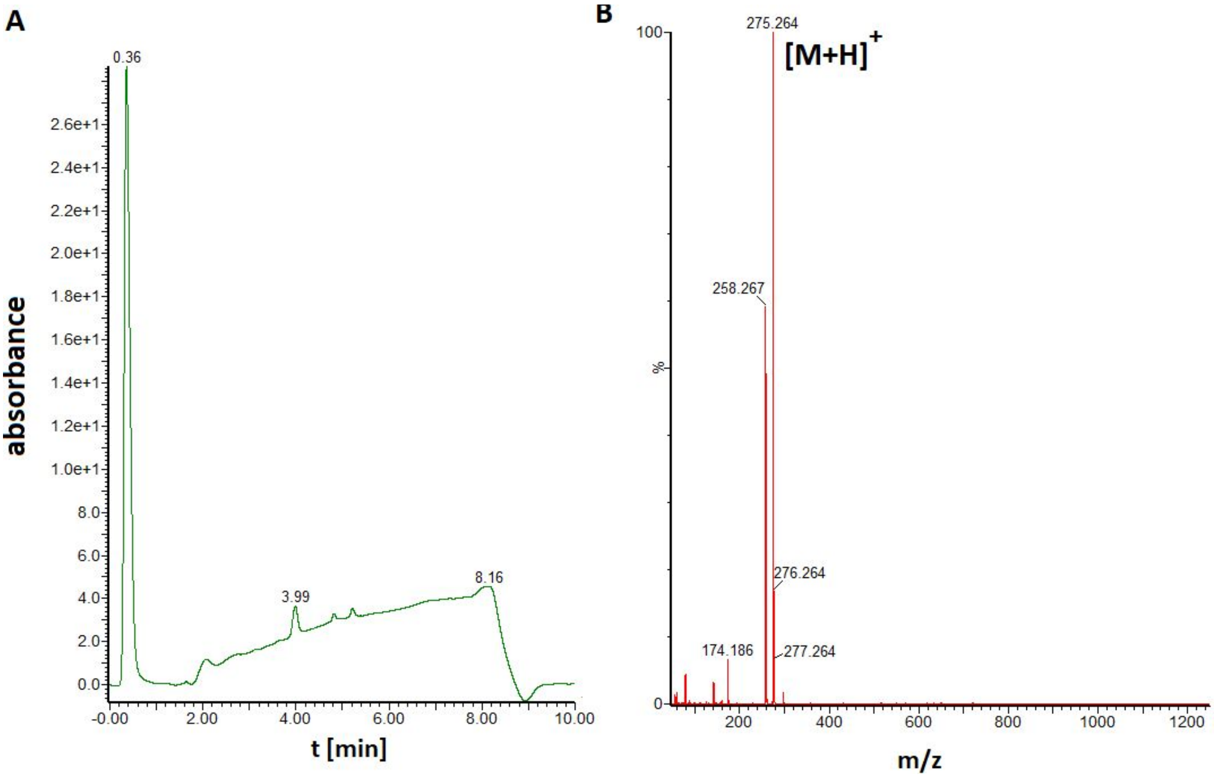

*Figure S 26: UPLC-MS Spectra of compound **25**. **A**. The photometric analysis was done between 200 to 400 nm. **B**. Mass spectra of the peak at 3.99 min.*

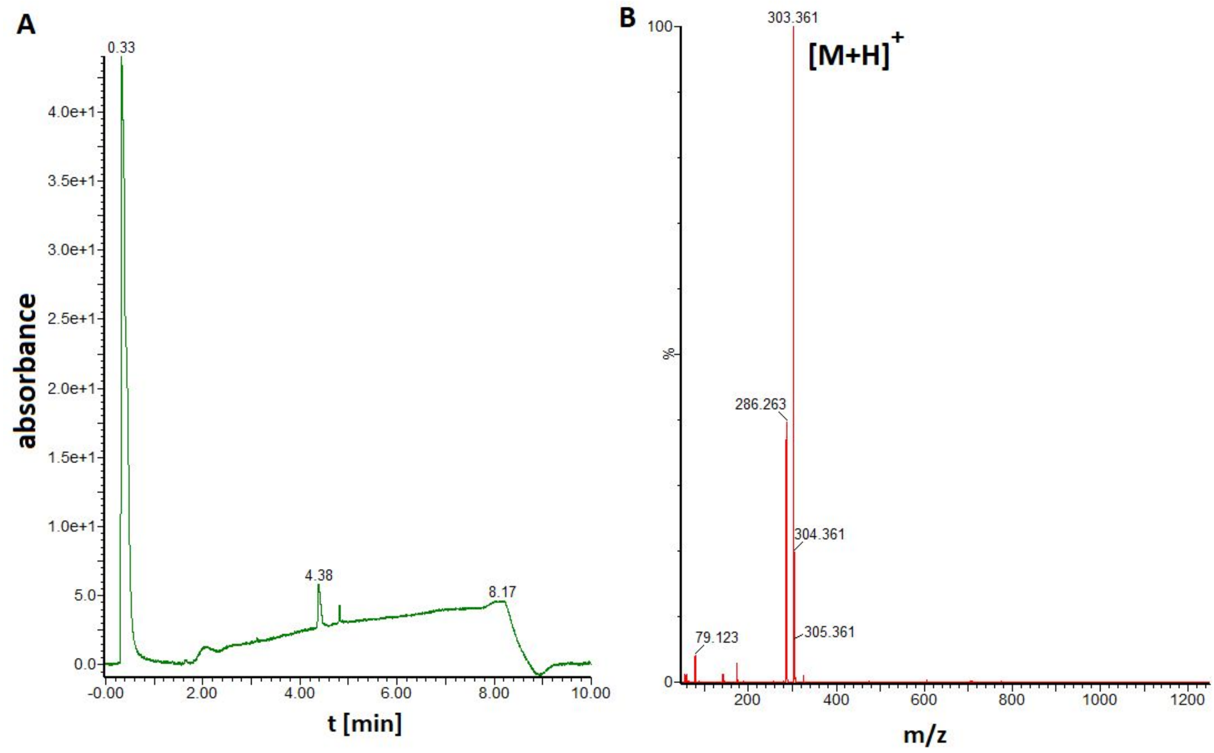

*Figure S 27: UPLC-MS Spectra of compound **26**. **A**. The photometric analysis was done between 200 to 400 nm. **B**. Mass spectra of the peak at 4.38 min.*

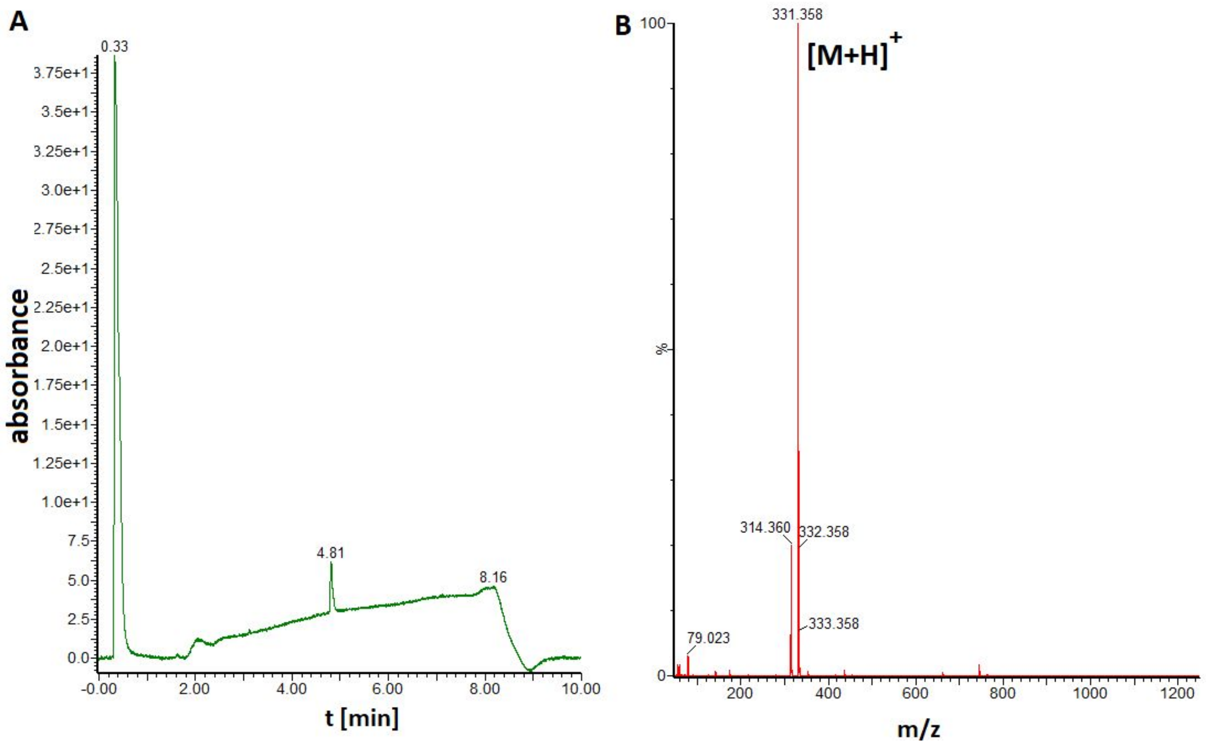

*Figure S 28: UPLC-MS Spectra of compound **27**. **A**. The photometric analysis was done between 200 to 400 nm. **B**. Mass spectra of the peak at 4.81 min.*

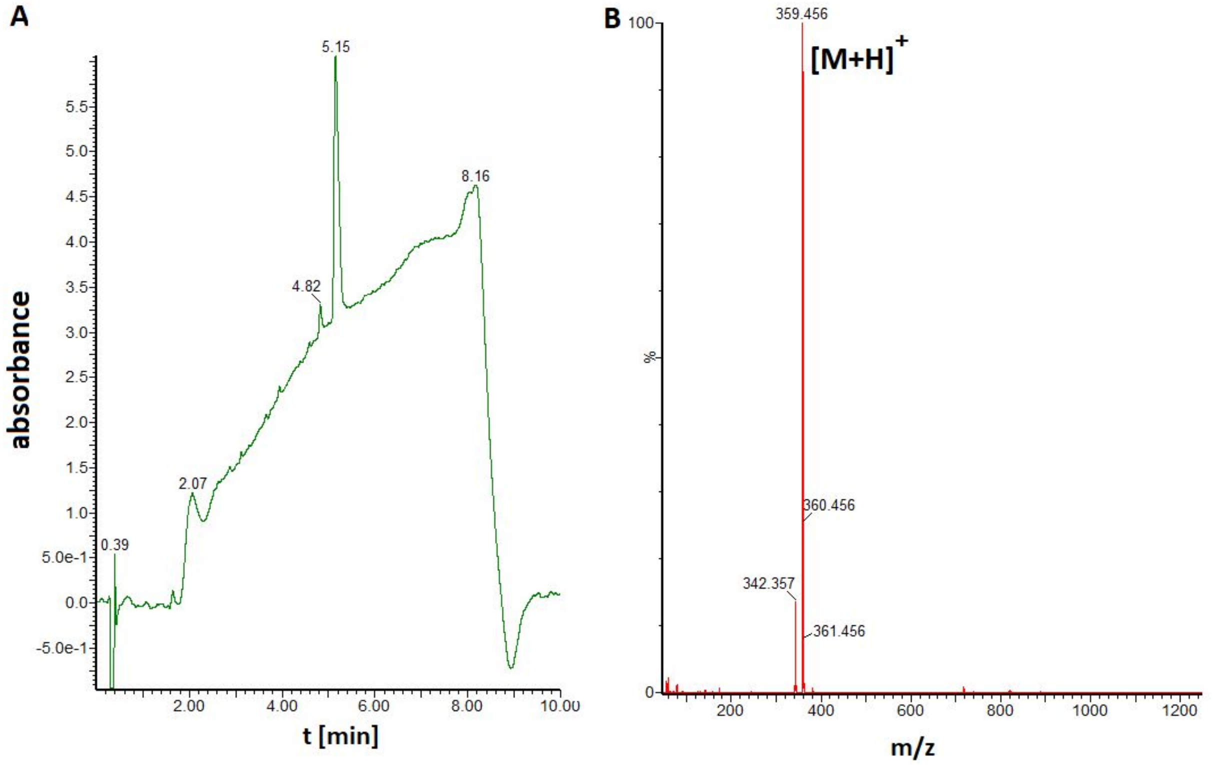

*Figure S 29: UPLC-MS Spectra of compound **28**. **A**. The photometric analysis was done between 200 to 400 nm. **B**. Mass spectra of the peak at 5.15 min.*

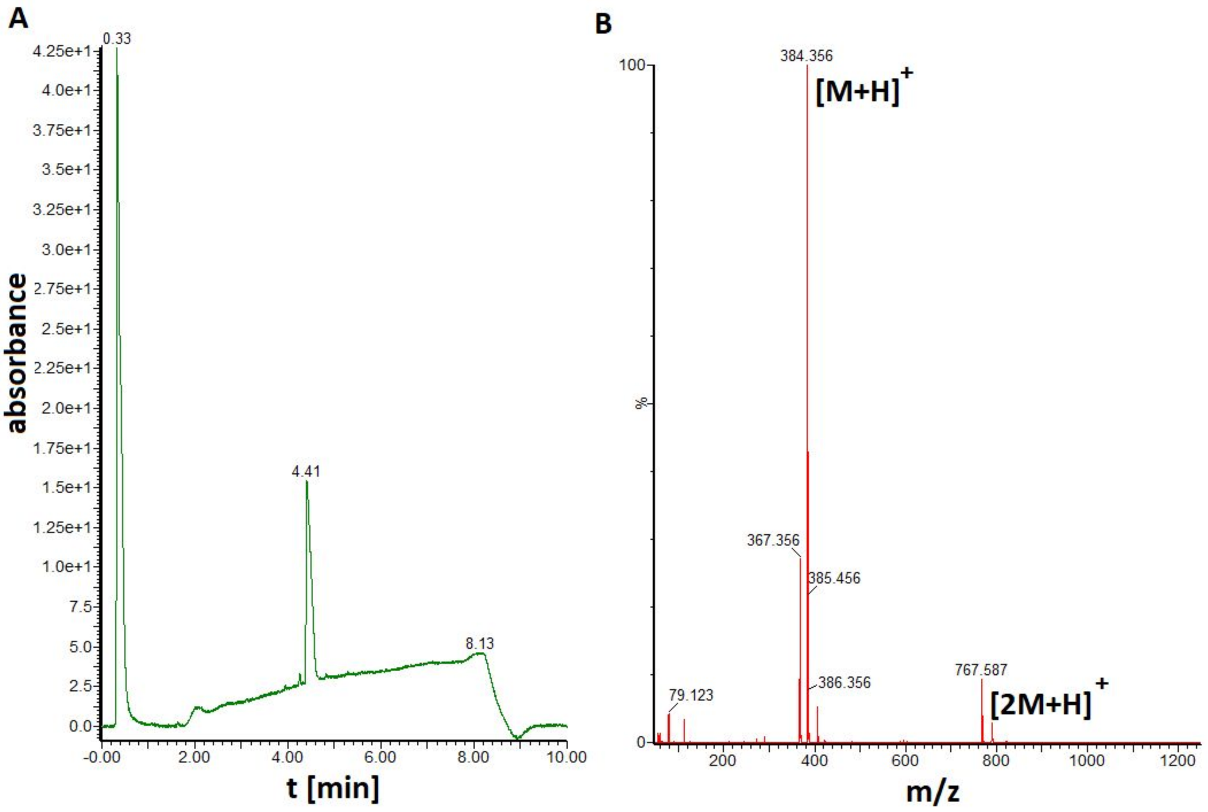

*Figure S 30: UPLC-MS Spectra of compound **29**. **A**. The photometric analysis was done between 200 to 400 nm. **B**. Mass spectra of the peak at 4.41 min.*

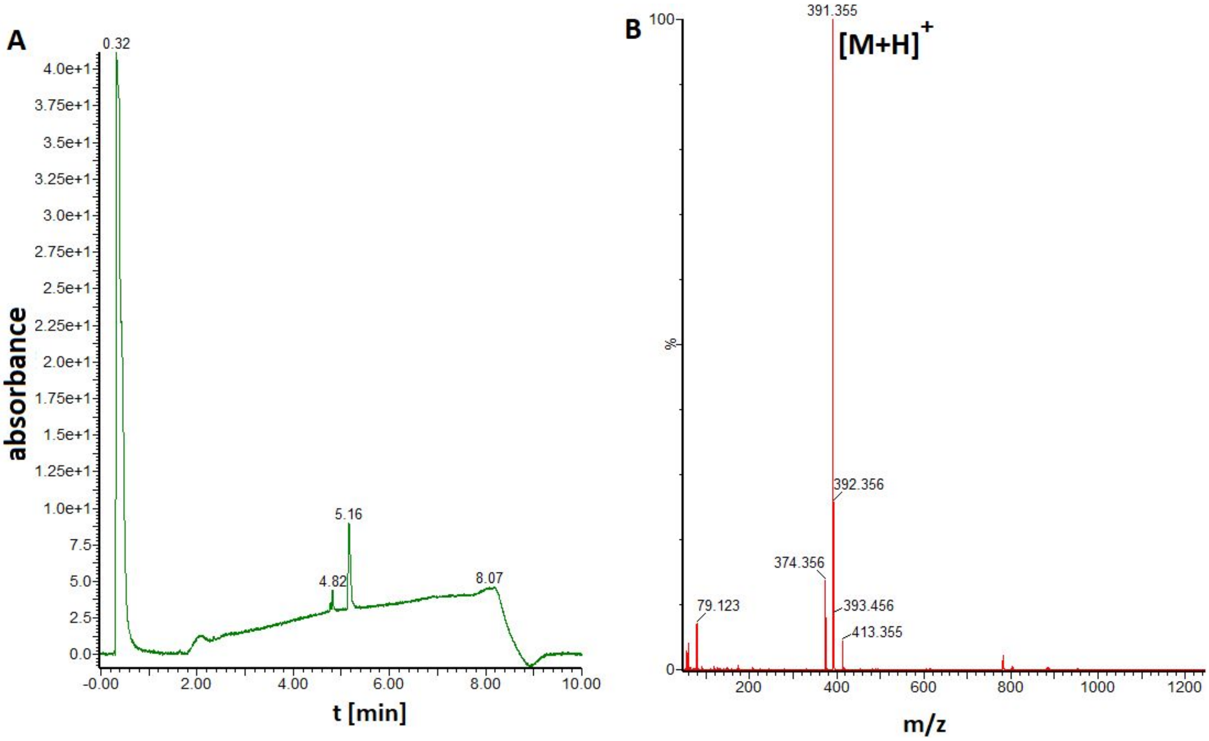

*Figure S 31: UPLC-MS Spectra of compound **30**. **A**. The photometric analysis was done between 200 to 400 nm. **B**. Mass spectra of the peak at 5.16 min.*

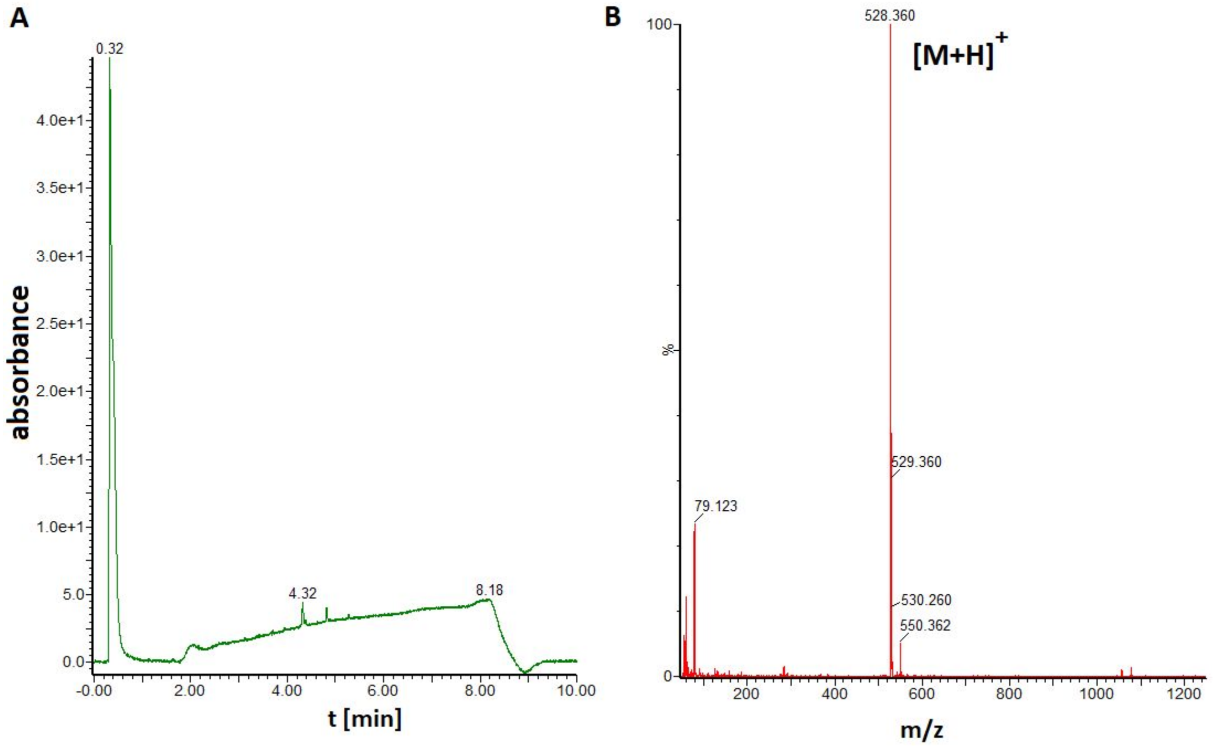

*Figure S 32: UPLC-MS Spectra of compound **31**. **A**. The photometric analysis was done between 200 to 400 nm. **B**. Mass spectra of the peak at 4.32 min.*

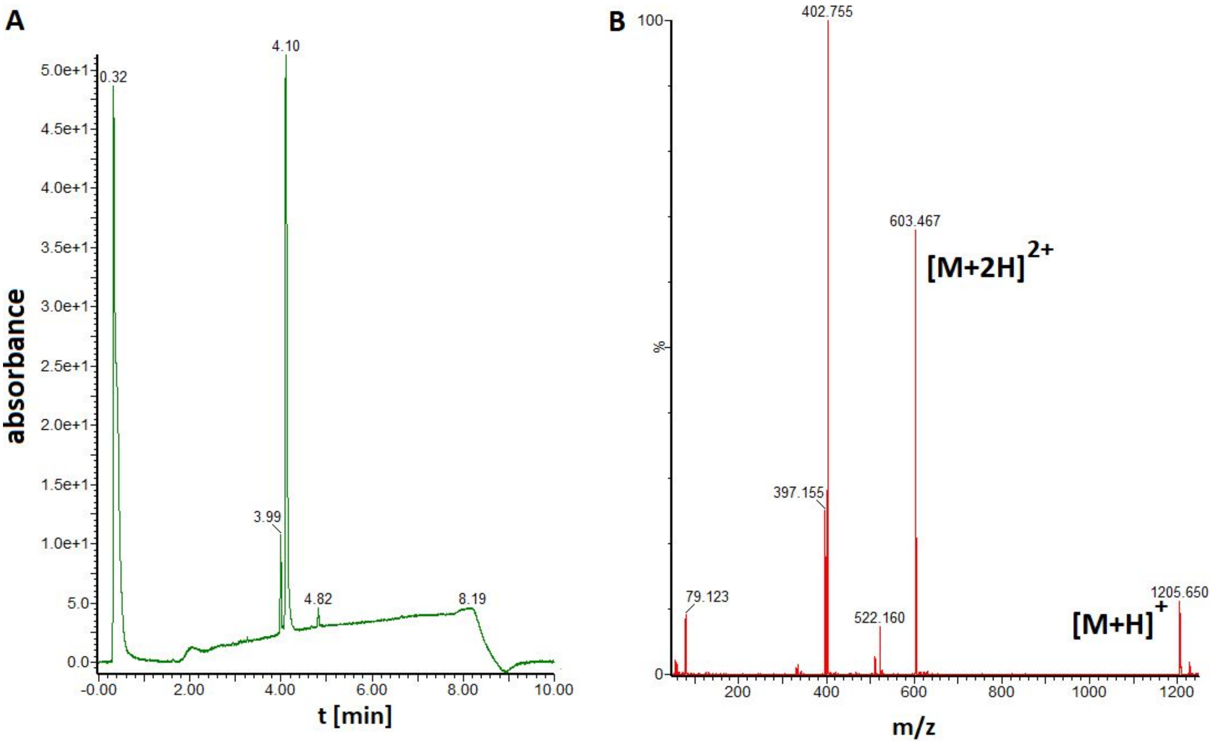

*Figure S 33: UPLC-MS Spectra of compound **32**. **A**. The photometric analysis was done between 200 to 400 nm. **B**. Mass spectra of the peak at 4.10 min.*

F. UPLC-MS Data of the substrate test

In order to ascertain whether peptides **2**, **10**, **17** and **18** are possible substrates of HDAC11, 50 µM of the peptides were incubated with 100 nM HDAC11 for 24 hours at 37°C. Subsequently, a sample of the solutions was subjected to analysis using UPLC-MS. The presence of the double-charged peptide representing the expected cleavage product Ac-TARKSTG-NH_2 of **2**, **10**, **17** and **18** with the m/z ratio of $[M+2H]^{2+}$ (381.45) was taken as an indicator of a HDAC11-catalysed reaction. Figure S34 shows exclusively HDAC11 in the assay buffer. No signal indicative for the product peptide was detected.

Analysis of the expected cleavage product Ac-TARKSTG-NH_2 itself resulted in a m/z chromatogram of the run with a scan for the m/z ratio of $[M+2H]^{2+}$(381.45+/- 0.5) showing a distinct peak at 0.40 min (Figure S35).

Figure S36 shows the m/z chromatograms of the runs containing the peptides of investigation, with a scan for the m/z ratio of the anticipated double-charged product. For peptides **2** (**A**) and **10** (**B**) no single recognisable peak is present at the expected time. However, distinct peaks can be seen around 0.40 min for peptides **17** (**C**) and **18** (**D**). It can therefore be assumed that **17** and **18** exhibit substrate properties for HDAC11.

Buffer with HDAC11 (100 nM) after 24h

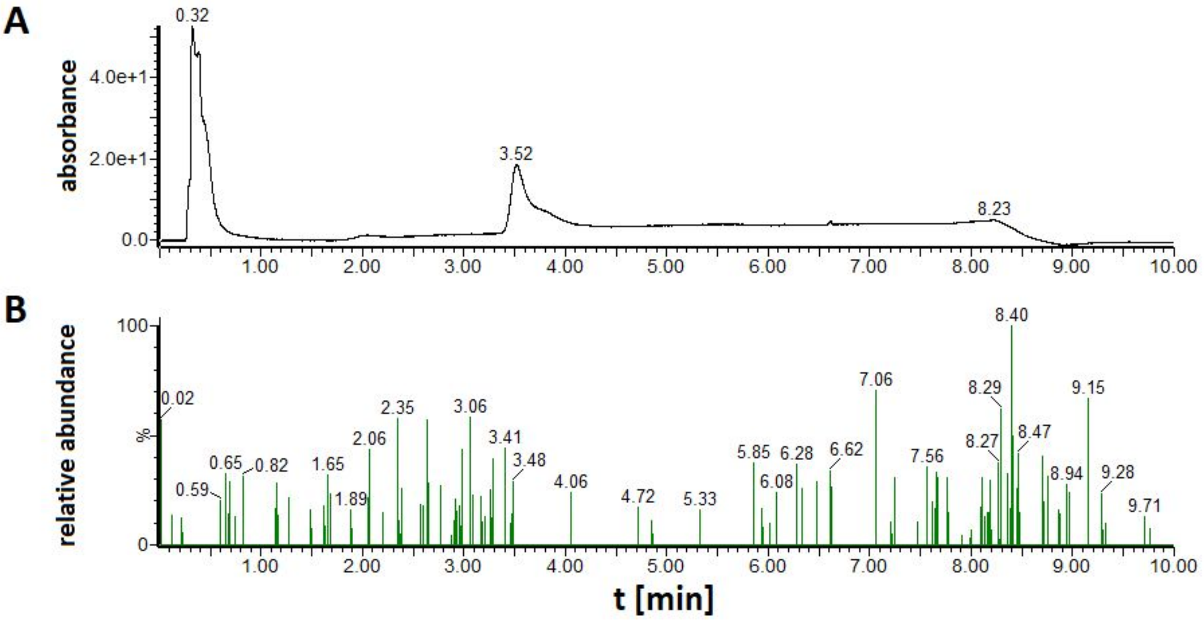

*Figure S 34: **A**. UPLC-MS analysis of HDAC11 in assay buffer. The photometric analysis was done between 200 to 400 nm. Absorbance at 220 nm is shown. The peak at 3.52 min corresponds to the BSA in the buffer. **B**. The mass spectra were scanned for the* expected *cleavage product $[M+2H]^{2+}$ with a m/z of 381.45.*

Buffer with expected cleavage product Ac-TARKSTG-NH_2 (control)

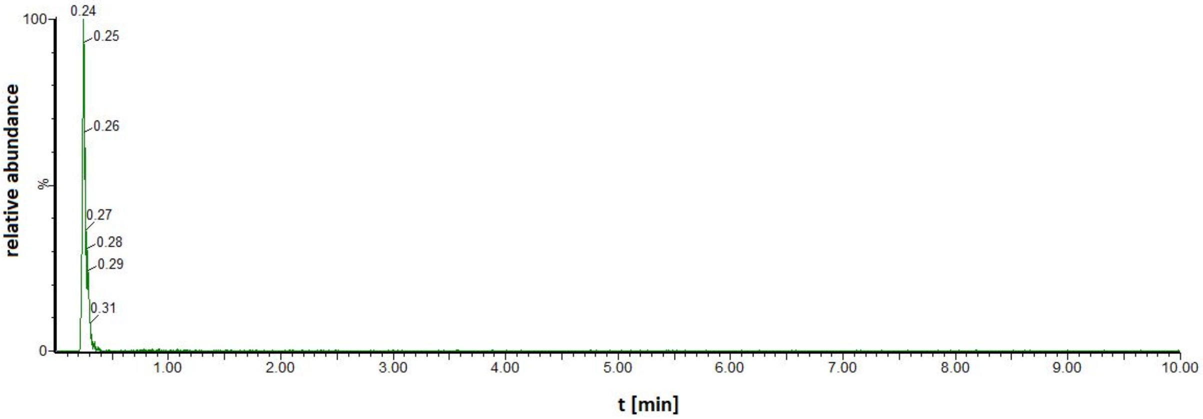

Figure S 35: UPLC-MS analysis of expected cleavage product Ac-TARKSTG-NH_2. The mass spectra were scanned for the expected *cleavage product $[M+2H]^{2+}$ with a m/z of 381.45.*

2, 10, 17 and 18 with HDAC11 (100 nM) after 24h

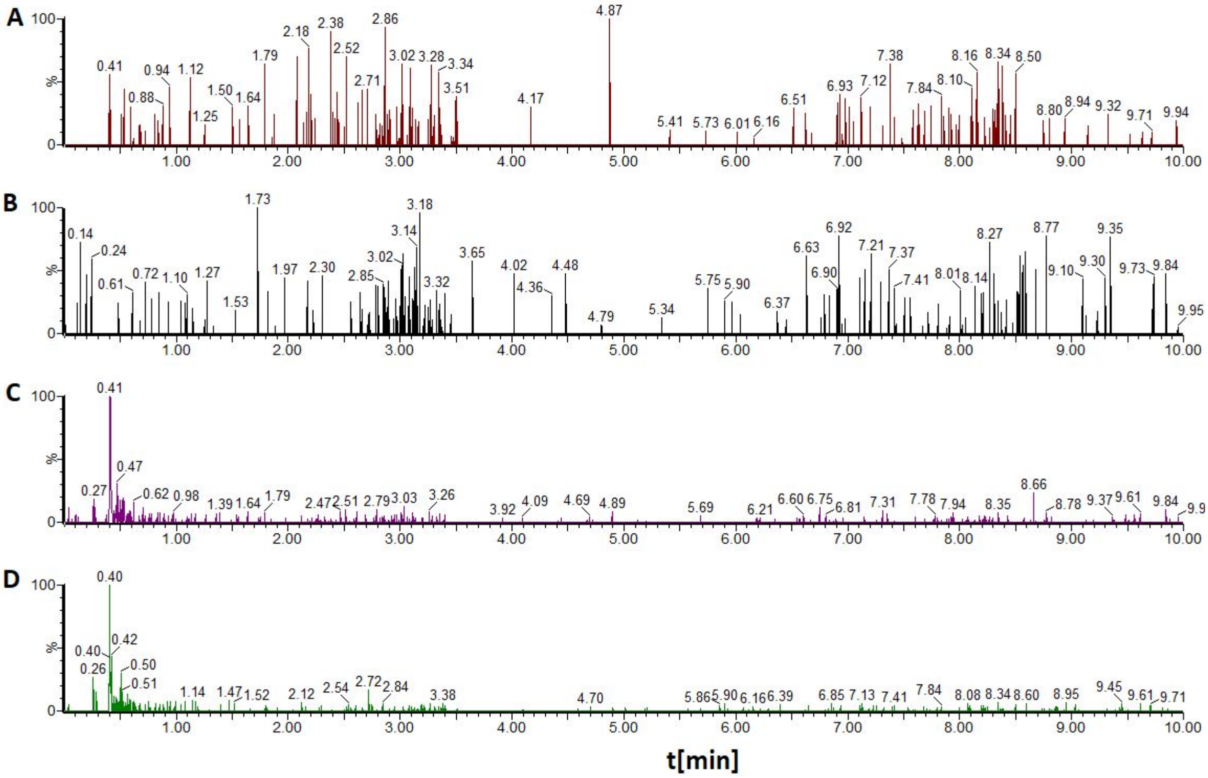

Figure S 36: Mass spectra scan for the expected cleavage product $[M+2H]^{2+}$ with a m/z of *381.45* after incubation of 50 µM of the respective peptide with 100 nM HDAC11 for 24 h at 37 °C. At t = 0.4 min the double charged species $[M+2H]^{2+}$ of the expected cleavage product was found for the reactions of **17** and **18**. **A** 2 **B** 10 **C** 17 **D** 18.

G. Docking of 31 and predicted binding mode

Docking

Schrodinger suite 2019 was used for the docking study. The ligand was prepared using LigPrep[9] panel with OPLS3e force fields [10-13]. The optimized HDAC11 AlphaFold2 model in complex with the alkylhydrazide SIS17 in the vertical pose was used as a receptor structure [14]. Receptor grids were generated using the Receptor Grid Generation panel by utilizing the centroid of the ligand. The ligand length was increased to 25 Å to account for the difference in ligand size. Docking was performed using Glide with standard precision and flexible ligand sampling and using OPLS3e force field [9, 15-17]. The amino function of the inhibitor was considered in the neutral form. Enhanced conformational sampling by four times was applied, while subjecting 250 poses to post docking minimization and reporting 20 poses. The best pose in terms of zinc ion chelation and hydrogen bond formation was then selected for analysis.

Predicted binding mode of compound 31

The binding mode of compound **31** was studied by molecular docking using an AlphaFold2 model of HDAC11 that was previously validated through molecular docking of reported selective HDAC11 ligands bearing diverse scaffolds and zinc-binding groups, augmented by molecular dynamics simulations of the apo and holo forms of the model [14, 18, 19]. In the obtained docking pose, the long alkyl chain of **31** was suitably accommodated in the foot pocket of HDAC11 along loop 3 and loop7, while forming hydrophobic interactions with Phe37, Trp42, Val45, Val137, Phe141, Phe152 and Cys153. The foot pocket believed to be an exit tunnel for the free acetate in class I HDAC is considered to harbour the acyl moieties of substrates [20]. **31** showed a bidentate chelation of the Zn^{2+} ion through the amino and the carbonyl group of the α-amino amide moiety formed by cysteine with distances of 2.38 Å and 2.34 Å, respectively. The cysteine moiety of **31** also formed two hydrogen bonds with His142 and Tyr304 through its amino and carbonyl group, respectively. Furthermore, another hydrogen bond was observed between the NH of the amide linkage and His143. Finally, one NH group of the DKP capping group formed a hydrogen bond with Glu94. A similar unusual interaction between an amino function in neutral form and the Zn^{2+} ion in HDAC has been shown for the related subtype HDAC8 in complex with an amino acid-based inhibitor (PDB ID 3SFF). However, it is worth noting that the amino acid based HDAC8 inhibitor is coordinating the zinc mainly through the NH_2 group of the amino acid with distance of 2.05 Å, while the carbonyl group is interacting with a water molecule bound to the flipped-out Tyr306 in HDAC8. This flipped-out conformation of Tyr306 in HDAC8 is a consequence of the bulky aromatic amino acid residue in 3SFF. In the case of the HDAC11 inhibitor **31** with its elongated and flexible alkyl chain, this is not necessary to obtain a favoured interaction in the catalytic center.

H Stability and permeability of 31

Stability

Chemical stability of compound **31** was analysed in human serum and cell lysate. Analysis of compound stability was performed as described [21]. Briefly, 400 µl of human serum or cell lysate from HEK293 cells (1 mg/ml) were incubated at 37 °C with 150 µM of **31** with 5% (*v/v*) DMSO. Samples of 45 µl were taken out at different time points and quenched with 50 µl of aqueous urea (6 M) and 100 µl of ACN. Following centrifugation, 2 µl of a 2% (*m/v*) K_2CO_3 solution in H_2O and 1.5 µl of a 0.2 M Fmoc-OSu in ACN were added to the samples, which were then shaken at 1000 rpm for two hours at room temperature. This enabled the quantification of the residual amount of the compound via UPLC-UV/VIS at 260 nm. Compound peak areas were integrated. The experiments were performed in triplicate. More than 20% of compound **31** was present after 6h incubation at 37°C. After 24h, 7% of **31** remained detectable in cell lysate, and 20 % of **31** were still present in human serum (Figure S 38).

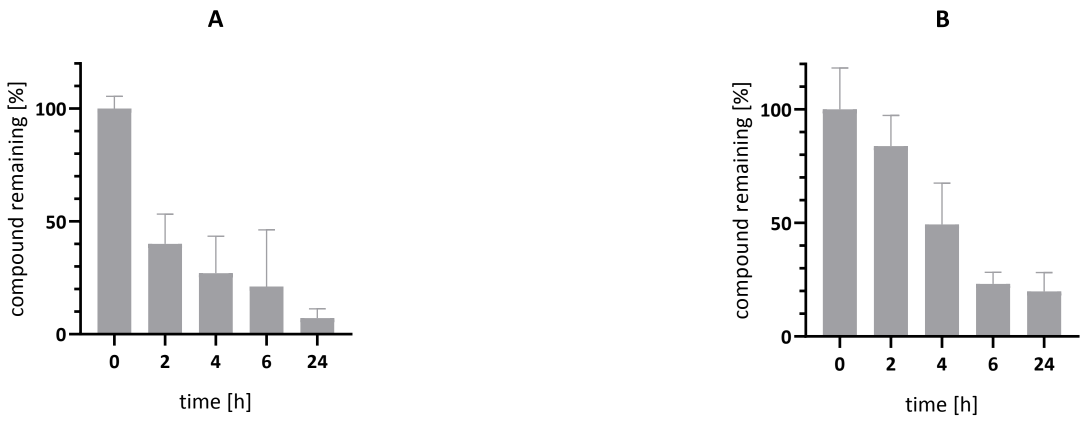

*Figure S 37: Stability analysis of compound **31** in HEK293 cell lysate (A) and human serum (B) after 2, 4, 6 and 24 h. Data are shown as mean values relative to the peak intensity at 0 h. Error bars represent the SD of three replicates.*

Cell permeability

For cell permeability studies, 40 µM of **31** were incubated with HEK293 cells in DMEM medium supplemented with 10 % FCS at 37°C overnight. The cells were harvested, washed extensively with ice-cold PBS and lysed in RIPA buffer. The sample was diluted 1:1 (*v/v*) with a solution of ACN : H_2O (1:1 (*v/v*)) before analyzing via UPLC-MS. UPLC-MS analysis was performed using a Waters Acquity UPLC-MS system (Milford, USA) with a Waters Acquity UPLC-MS-BEH C18 column (1.7 µM, 2.1 × 50 mm; 30 Å). Data analysis was performed using Waters MassLynx software. Elution was performed at a flow rate of 0.3 ml/min at room temperature using as mobile phase a mixture of 0.1% (*v/v*) formic acid in deionized water (solvent A) and a mixture of 0.1% (*v/v*) formic acid in ACN (solvent B). The gradient of B was from 5 to 100% in 6 min.

Presence of compound **31** in the cell lysate from HEK293 cells incubated with the compound detected by mass spectrometry inducates uptake of compound **31** by HEK293 cells (Figure S 38).

A

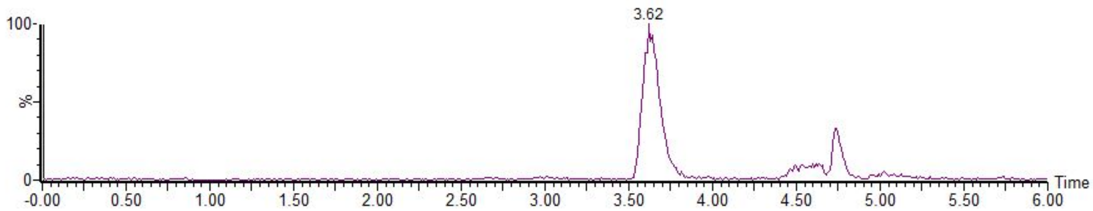

B

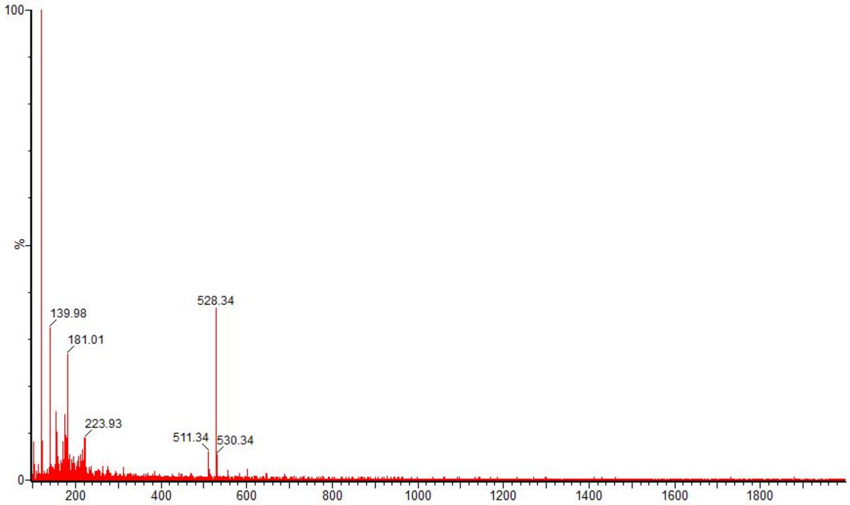

Figure S 38: Permeability analysis of compound ***31*** *in HEK293 cells using mass spectrometry. A Chromatogram of the mass spectrum at an m/z of 528, which corresponds to the [M+H]$^+$ of compound* ***31****. B Mass spectrum of the peak detected at 3.62 min.*

References

1. Schuster, S.; Roessler, C.; Meleshin, M.; Zimmermann, P.; Simic, Z.; Kambach, C.; Schiene-Fischer, C.; Steegborn, C.; Hottiger, M. O.; Schutkowski, M. A continuous sirtuin activity assay without any coupling to enzymatic or chemical reactions. *Sci Rep* **2016**, *6*, 22643, DOI: 10.1038/srep22643.
2. Kutil, Z.; Mikesova, J.; Zessin, M.; Meleshin, M.; Novakova, Z.; Alquicer, G.; Kozikowski, A.; Sippl, W.; Barinka, C.; Schutkowski, M. Continuous Activity Assay for HDAC11 Enabling Reevaluation of HDAC Inhibitors. *ACS Omega* **2019**, *4* (22), 19895-19904, DOI: 10.1021/acsomega.9b02808.
3. Zessin, M.; Meleshin, M.; Hilscher, S.; Schiene-Fischer, C.; Barinka, C.; Jung, M.; Schutkowski, M. Continuous Fluorescent Sirtuin Activity Assay Based on Fatty Acylated Lysines. *Int J Mol Sci* **2023**, *24* (8), DOI: 10.3390/ijms24087416.
4. Noritsugu, K.; Suzuki, T.; Dodo, K.; Ohgane, K.; Ichikawa, Y.; Koike, K.; Morita, S.; Umehara, T.; Ogawa, K.; Sodeoka, M.; Dohmae, N.; Yoshida, M.; Ito, A. Lysine long-chain fatty acylation regulates the TEAD transcription factor. *Cell Rep* **2023**, *42* (4), 112388, DOI: 10.1016/j.celrep.2023.112388.
5. Ignatova, Y. L.; Karimova, N. M.; Rozhkov, I. N. Nucleophilic-Substitution of Bromine in Vicinal Bromotrifluoroalkylamines. *Russian Chemical Bulletin* **1994**, *43* (5), 900-903, DOI: Doi 10.1007/Bf00717368.
6. Arrica, M. A.; Wirth, T. Fluorinations of α-Seleno Carboxylic Acid Derivatives with Hypervalent (Difluoroiodo)toluene. *European Journal of Organic Chemistry* **2005**, *2005* (2), 395-403, DOI: 10.1002/ejoc.200400659.
7. Koppitz, M.; Huenges, M.; Gratias, R.; Kessler, H.; Goodman, S. L.; Jonczyk, A. Synthesis of Unnatural Lipophilic N-(9H-Fluoren-9-ylmethoxy)carbonyl-Substituted α-Amino Acids and Their Incorporation into Cyclic RGD-Peptides: A structure-activity study. *Helvetica Chimica Acta* **2004**, *80* (4), 1280-1300, DOI: 10.1002/hlca.19970800423.
8. Nishimura, O.; Suenaga, M.; Ohmae, H.; Tsuji, S.; Suenaga, M.; Fujino, M. An efficient chemical method for removing N-terminal extra methionine from recombinant methionylated human growth hormone. *Chemical Communications* **1998**, (10), 1135-1136, DOI: 10.1039/a801297k.
9. Schrödinger Release 2019-1: LigPrep, Schrödinger, LLC, New York, NY, . **2019**, DOI.
10. Harder, E.; Damm, W.; Maple, J.; Wu, C.; Reboul, M.; Xiang, J. Y.; Wang, L.; Lupyan, D.; Dahlgren, M. K.; Knight, J. L.; Kaus, J. W.; Cerutti, D. S.; Krilov, G.; Jorgensen, W. L.; Abel, R.; Friesner, R. A. OPLS3: A Force Field Providing Broad Coverage of Drug-like Small Molecules and Proteins. *J Chem Theory Comput* **2016**, *12* (1), 281-96, DOI: 10.1021/acs.jctc.5b00864.
11. Jorgensen, W. L.; Maxwell, D. S.; Tirado-Rives, J. Development and Testing of the OPLS All-Atom Force Field on Conformational Energetics and Properties of Organic Liquids. *Journal of the American Chemical Society* **1996**, *118* (45), 11225-11236, DOI: 10.1021/ja9621760.
12. Jorgensen, W. L.; Tirado-Rives, J. The OPLS [optimized potentials for liquid simulations] potential functions for proteins, energy minimizations for crystals of cyclic peptides and crambin. *J Am Chem Soc* **1988**, *110* (6), 1657-66, DOI: 10.1021/ja00214a001.
13. Shivakumar, D.; Williams, J.; Wu, Y.; Damm, W.; Shelley, J.; Sherman, W. Prediction of Absolute Solvation Free Energies using Molecular Dynamics Free Energy Perturbation and the OPLS Force Field. *J Chem Theory Comput* **2010**, *6* (5), 1509-19, DOI: 10.1021/ct900587b.
14. Baselious, F.; Robaa, D.; Sippl, W. Utilization of AlphaFold models for drug discovery: Feasibility and challenges. Histone deacetylase 11 as a case study. *Comput Biol Med* **2023**, *167*, 107700, DOI: 10.1016/j.compbiomed.2023.107700.
15. Friesner, R. A.; Banks, J. L.; Murphy, R. B.; Halgren, T. A.; Klicic, J. J.; Mainz, D. T.; Repasky, M. P.; Knoll, E. H.; Shelley, M.; Perry, J. K.; Shaw, D. E.; Francis, P.; Shenkin, P. S. Glide: a new

approach for rapid, accurate docking and scoring. 1. Method and assessment of docking accuracy. *J Med Chem* **2004**, *47* (7), 1739-49, DOI: 10.1021/jm0306430.

16. Friesner, R. A.; Murphy, R. B.; Repasky, M. P.; Frye, L. L.; Greenwood, J. R.; Halgren, T. A.; Sanschagrin, P. C.; Mainz, D. T. Extra precision glide: docking and scoring incorporating a model of hydrophobic enclosure for protein-ligand complexes. *J Med Chem* **2006**, *49* (21), 6177-96, DOI: 10.1021/jm051256o.
17. Halgren, T. A.; Murphy, R. B.; Friesner, R. A.; Beard, H. S.; Frye, L. L.; Pollard, W. T.; Banks, J. L. Glide: a new approach for rapid, accurate docking and scoring. 2. Enrichment factors in database screening. *J Med Chem* **2004**, *47* (7), 1750-9, DOI: 10.1021/jm030644s.
18. Baselious, F.; Hilscher, S.; Hagemann, S.; Tripathee, S.; Robaa, D.; Barinka, C.; Hüttelmaier, S.; Schutkowski, M.; Sippl, W. Utilization of an Optimized AlphaFold Protein Model for Structure-Based Design of a Selective HDAC11 Inhibitor with Anti-neuroblastoma Activity. **2024**, DOI: 10.26434/chemrxiv-2024-6f790.
19. Baselious, F.; Hilscher, S.; Robaa, D.; Barinka, C.; Schutkowski, M.; Sippl, W. Comparative Structure-Based Virtual Screening Utilizing Optimized AlphaFold Model Identifies Selective HDAC11 Inhibitor. *Int J Mol Sci* **2024**, *25* (2), DOI: 10.3390/ijms25021358.
20. Kutil, Z.; Novakova, Z.; Meleshin, M.; Mikesova, J.; Schutkowski, M.; Barinka, C. Histone Deacetylase 11 Is a Fatty-Acid Deacylase. *ACS Chem Biol* **2018**, *13* (3), 685-693, DOI: 10.1021/acschembio.7b00942.
21. Rajabi, N.; Lund Nielsen, A.; Olsen, C. A. Dethioacylation by Sirtuins 1–3: Considerations for Drug Design Using Mechanism-Based Sirtuin Inhibition. *ACS Med. Chem. Lett* **2019**, *11*, 1886– 1892, DOI: 10.1021/acsmedchemlett.9b00580
